# Supplementary material for: A bioinspired and biocompatible ortho-sulfiliminyl phenol synthesis
Source: Nat Commun. 2017 Jun 19;8:15912. doi: 10.1038/ncomms15912 (PMC5481830; doi:10.1038/ncomms15912)
Supplement: Supplementary Information [file ncomms15912-s1.pdf]

Type of file: PDF
Size of file: 0 KB
Title of file for HTML: Supplementary Information
Description: Supplementary figures, supplementary tables, supplementary discussion, supplementary methods and supplementary references.

Type of file: PDF
Size of file: 0 KB
Title of file for HTML: Peer review file
Description:

Supplementary Figures

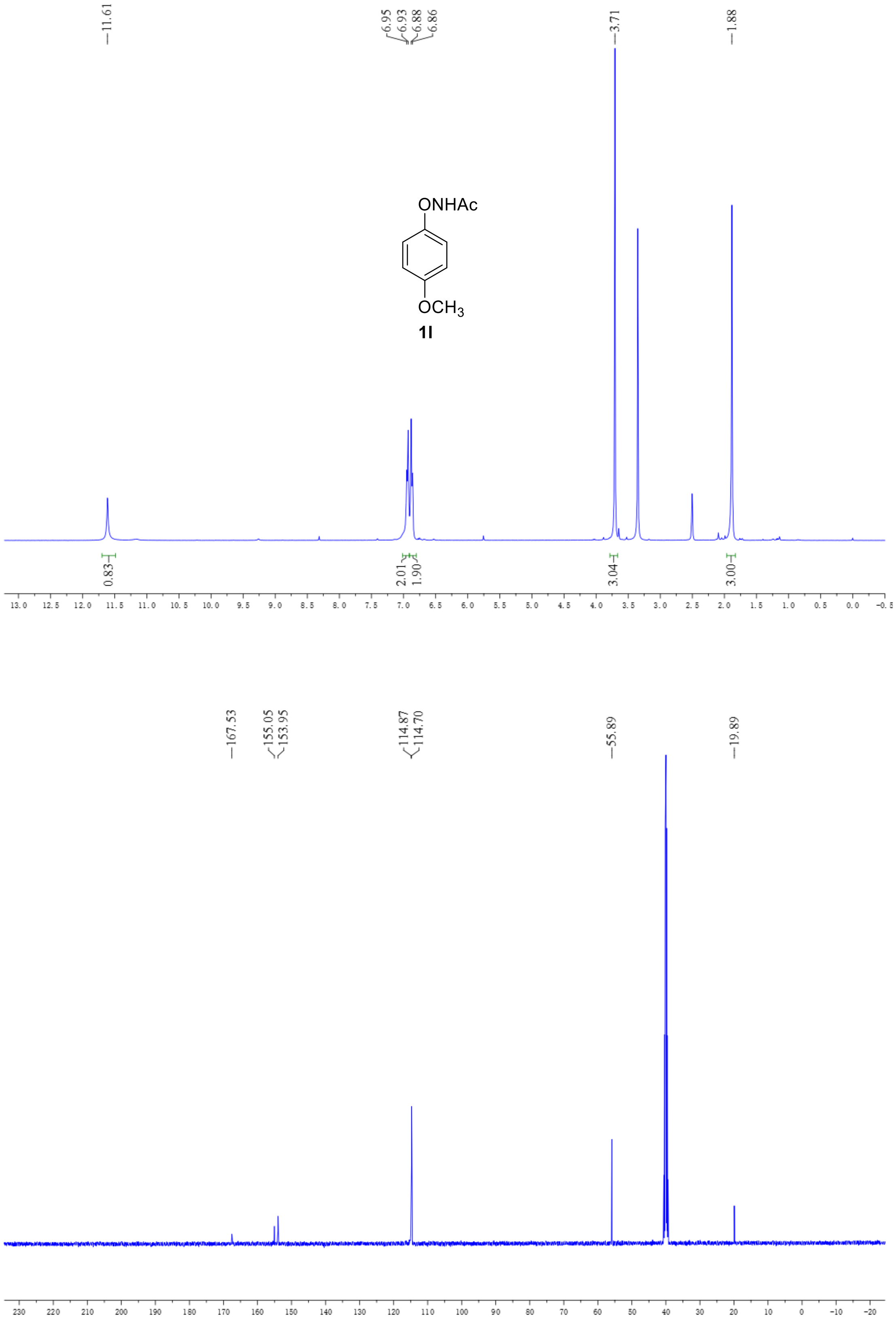

Supplementary Figure 1. ^{1}H and ^{13}C NMR spectra for compound **1l**

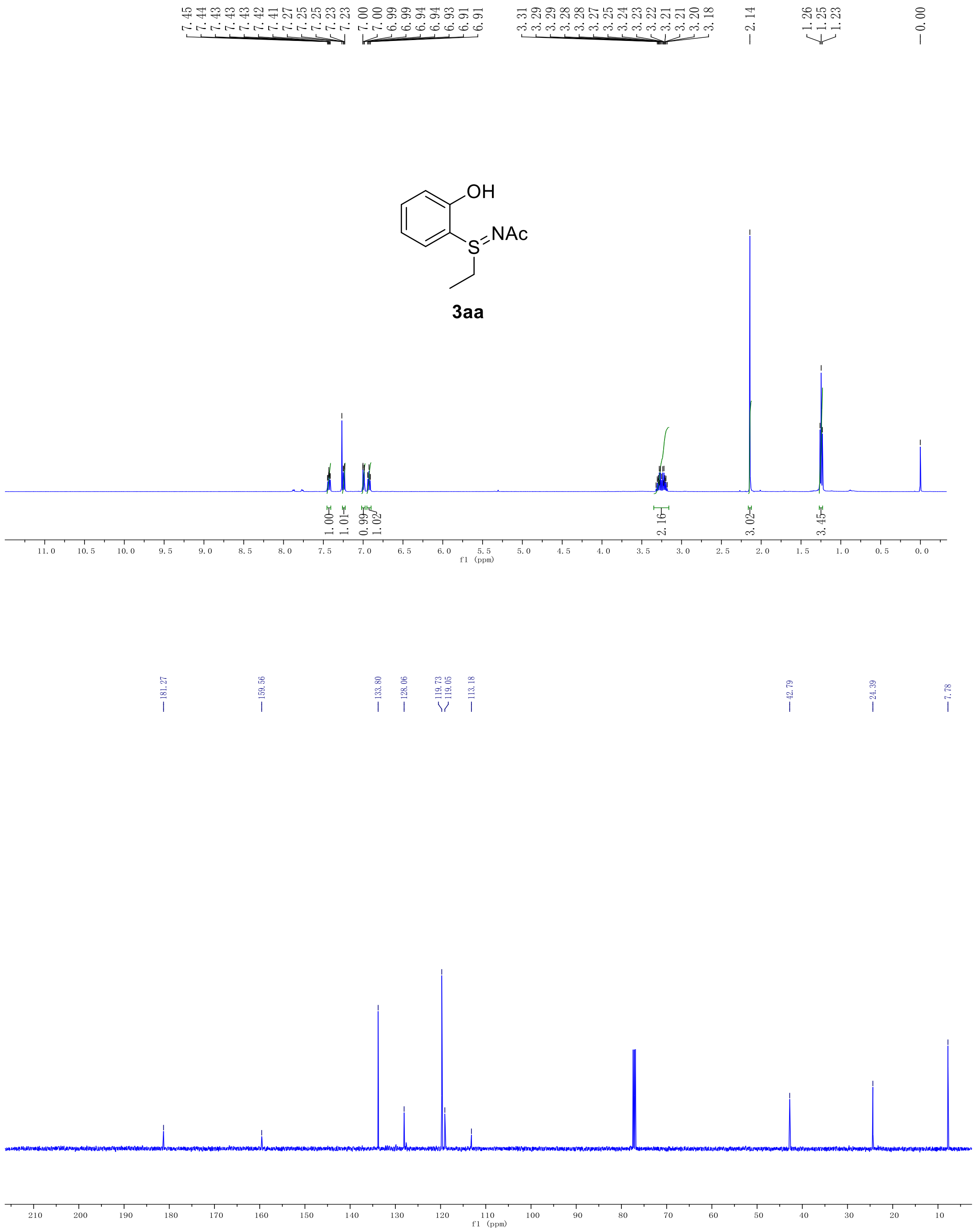

Supplementary Figure 2. ^{1}H and ^{13}C NMR spectra for compound **3aa**

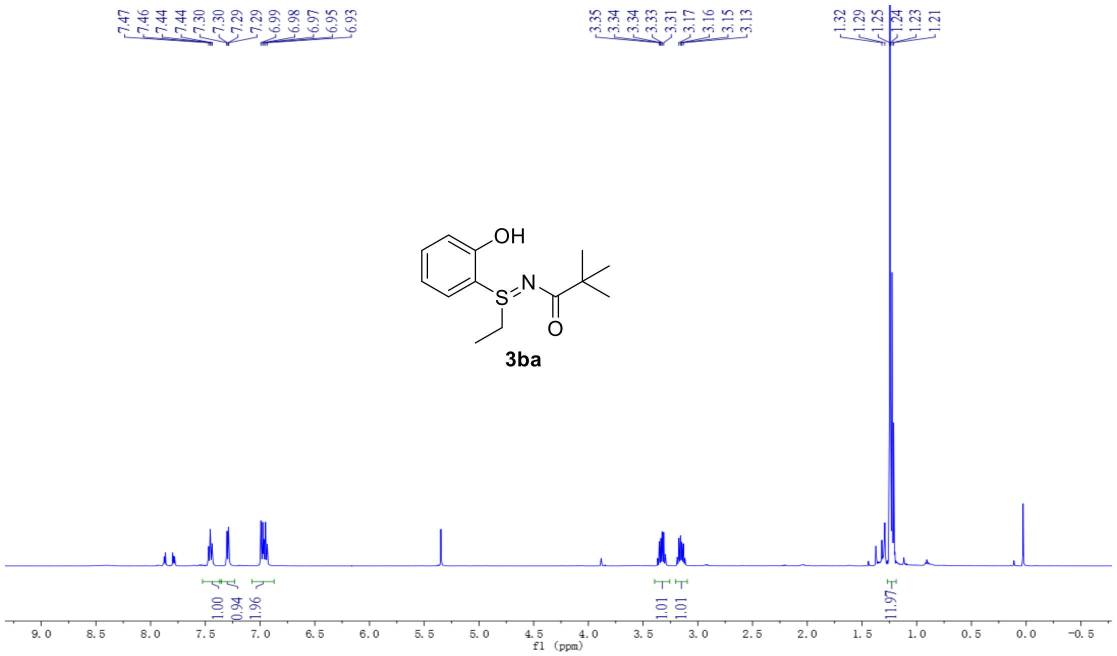

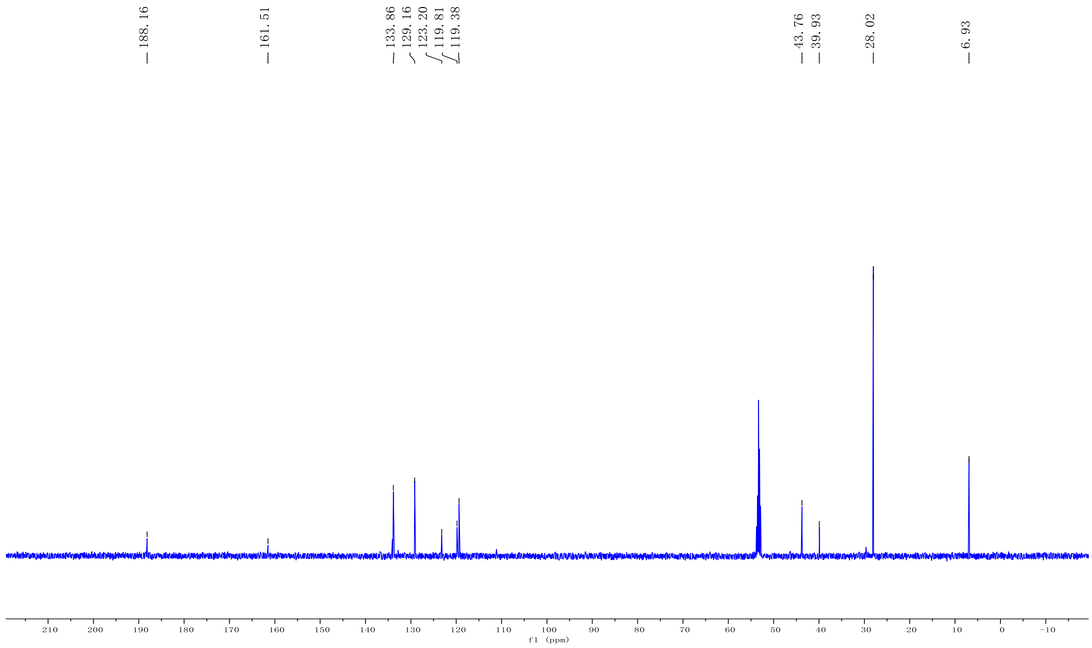

Supplementary Figure 3. ^{1}H and ^{13}C NMR spectra for compound **3ba**

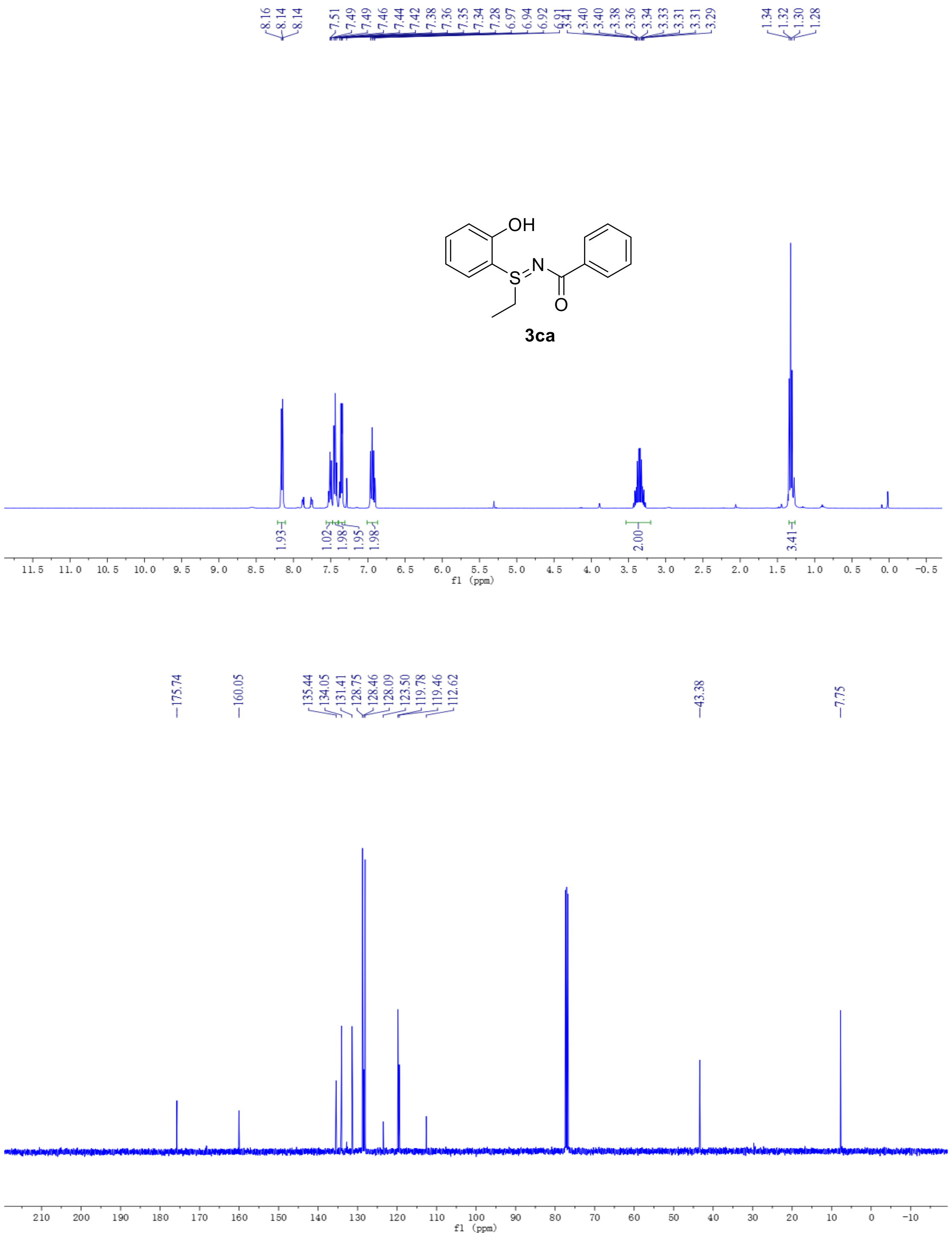

Supplementary Figure 4. ^{1}H and ^{13}C NMR spectra for compound **3ca**

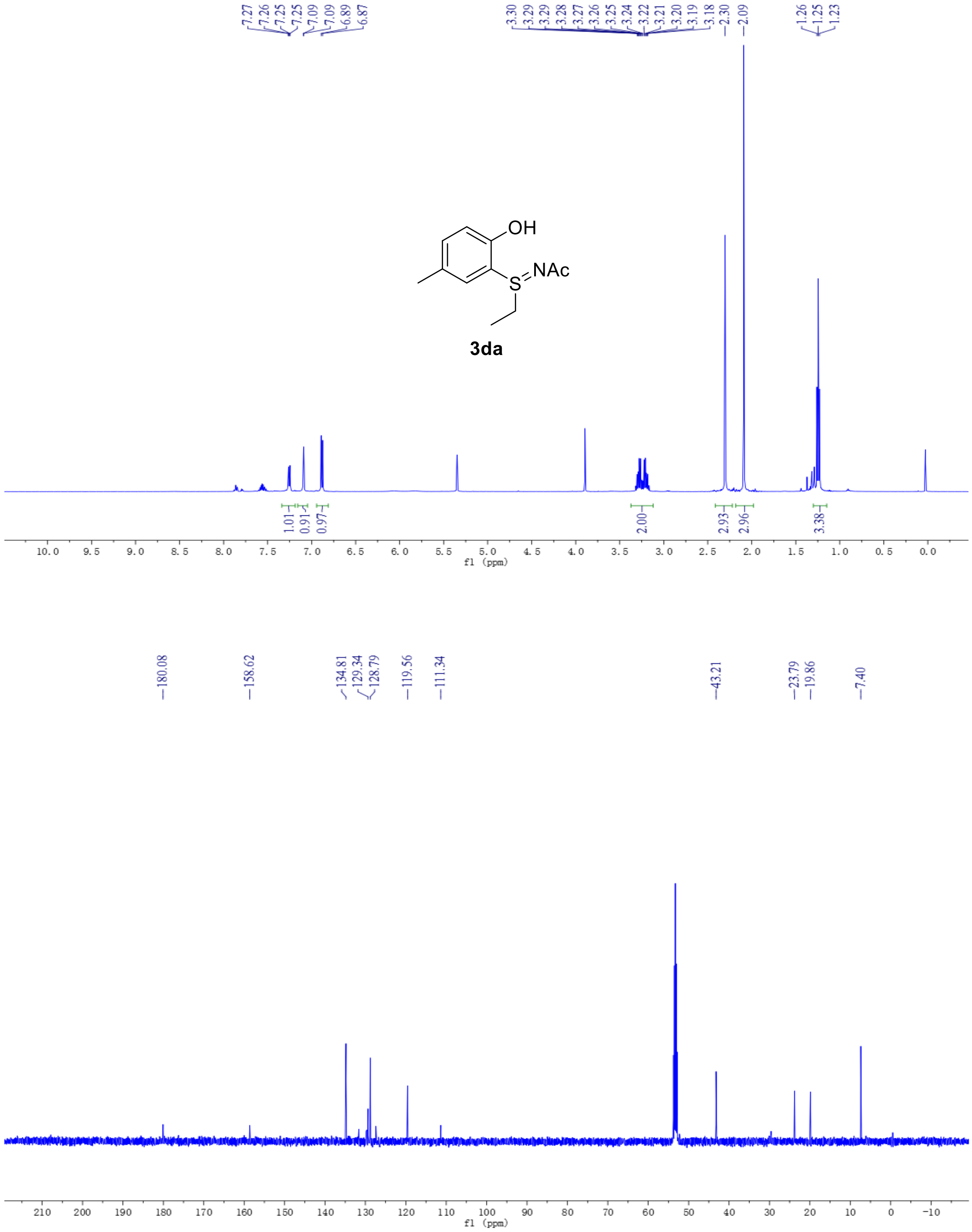

Supplementary Figure 5. ^{1}H and ^{13}C NMR spectra for compound **3da**

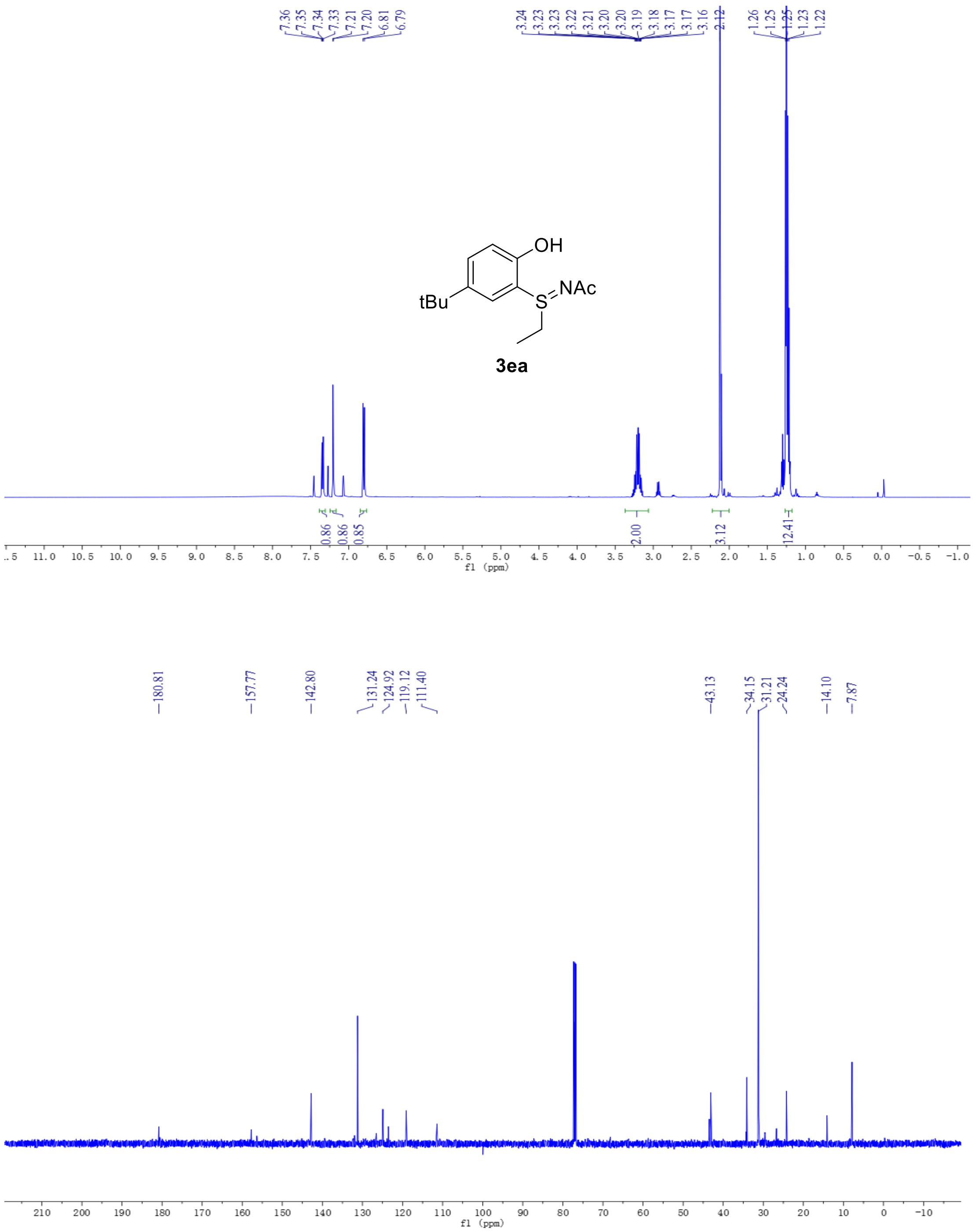

Supplementary Figure 6. ^{1}H and ^{13}C NMR spectra for compound **3ea**

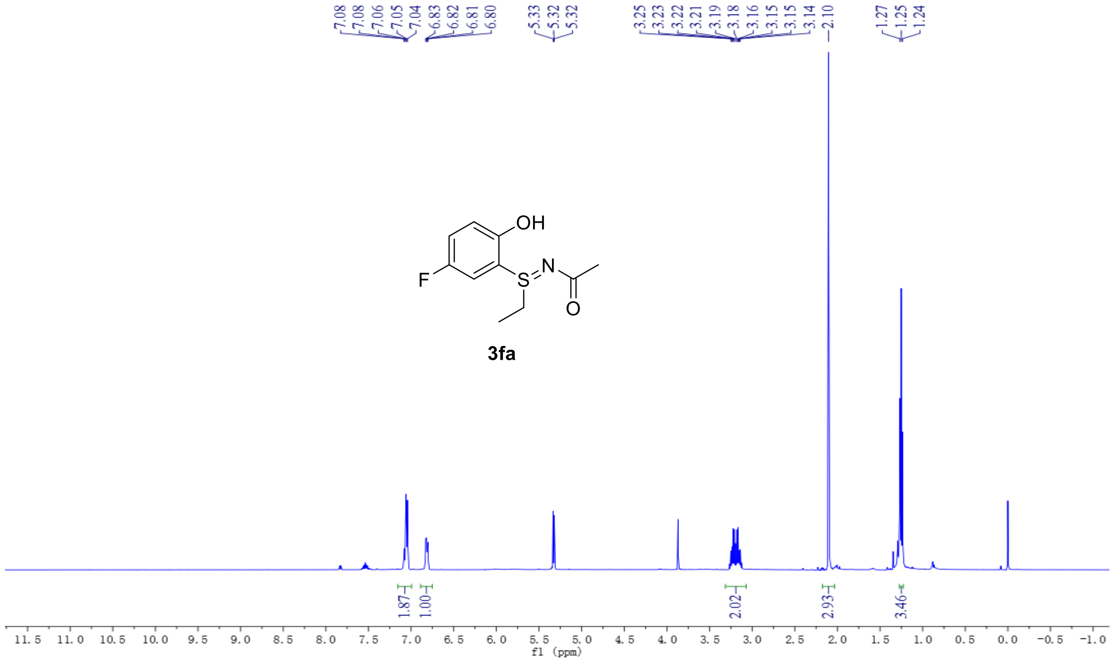

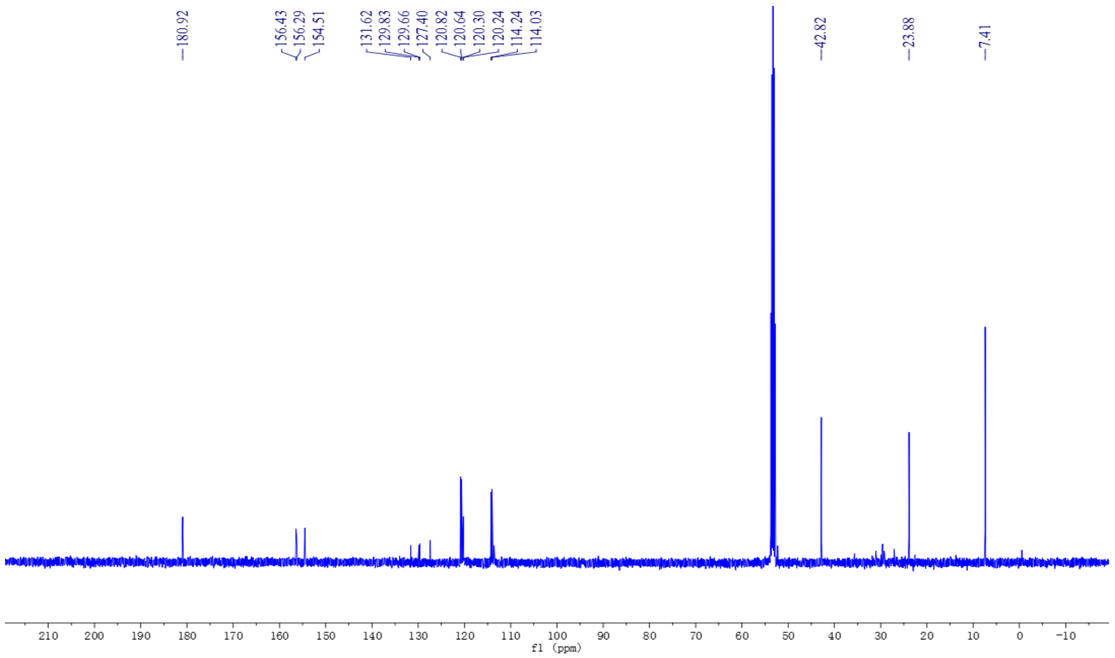

Supplementary Figure 7. ^{1}H and ^{13}C NMR spectra for compound **3fa**

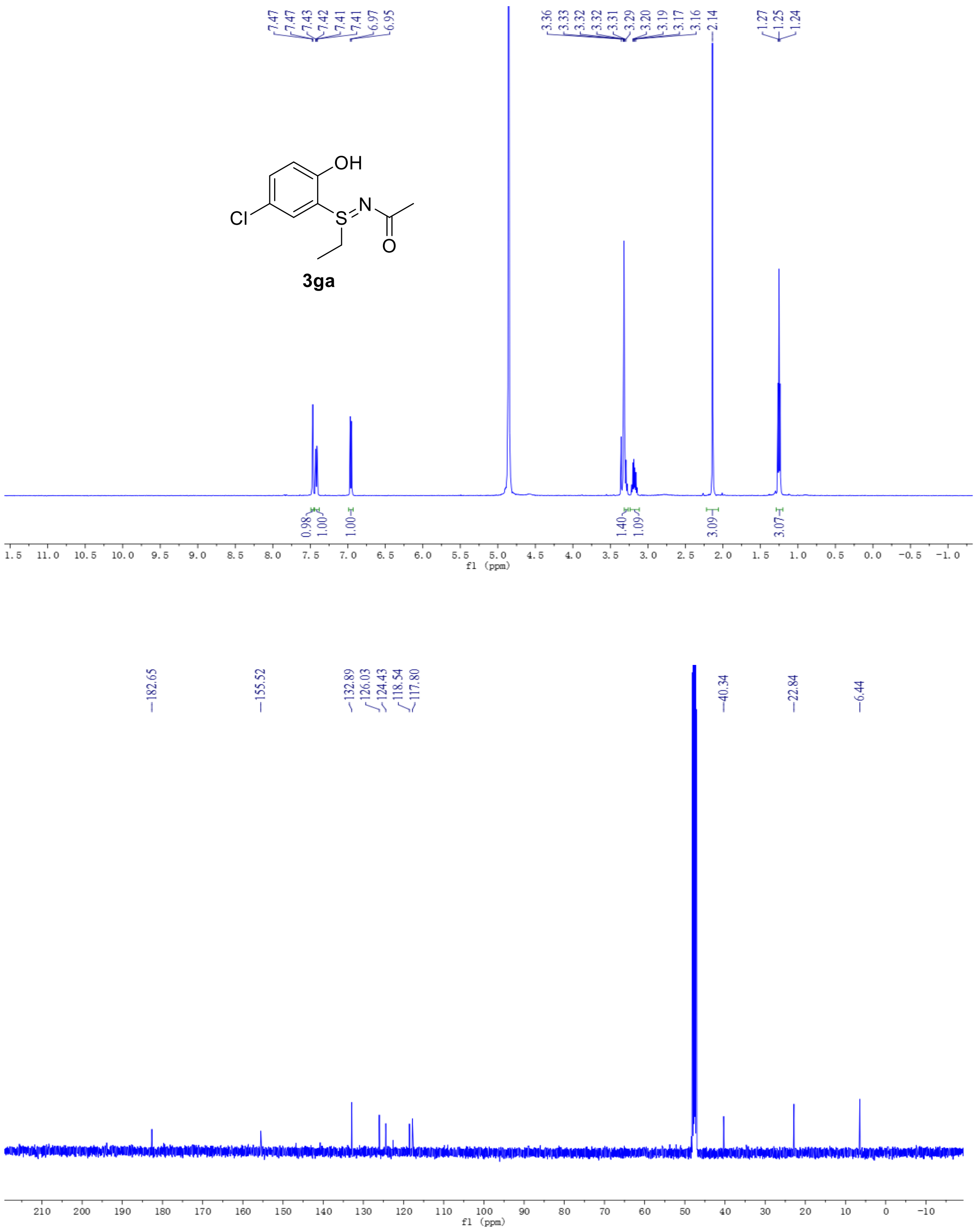

Supplementary Figure 8. ^{1}H and ^{13}C NMR spectra for compound **3ga**

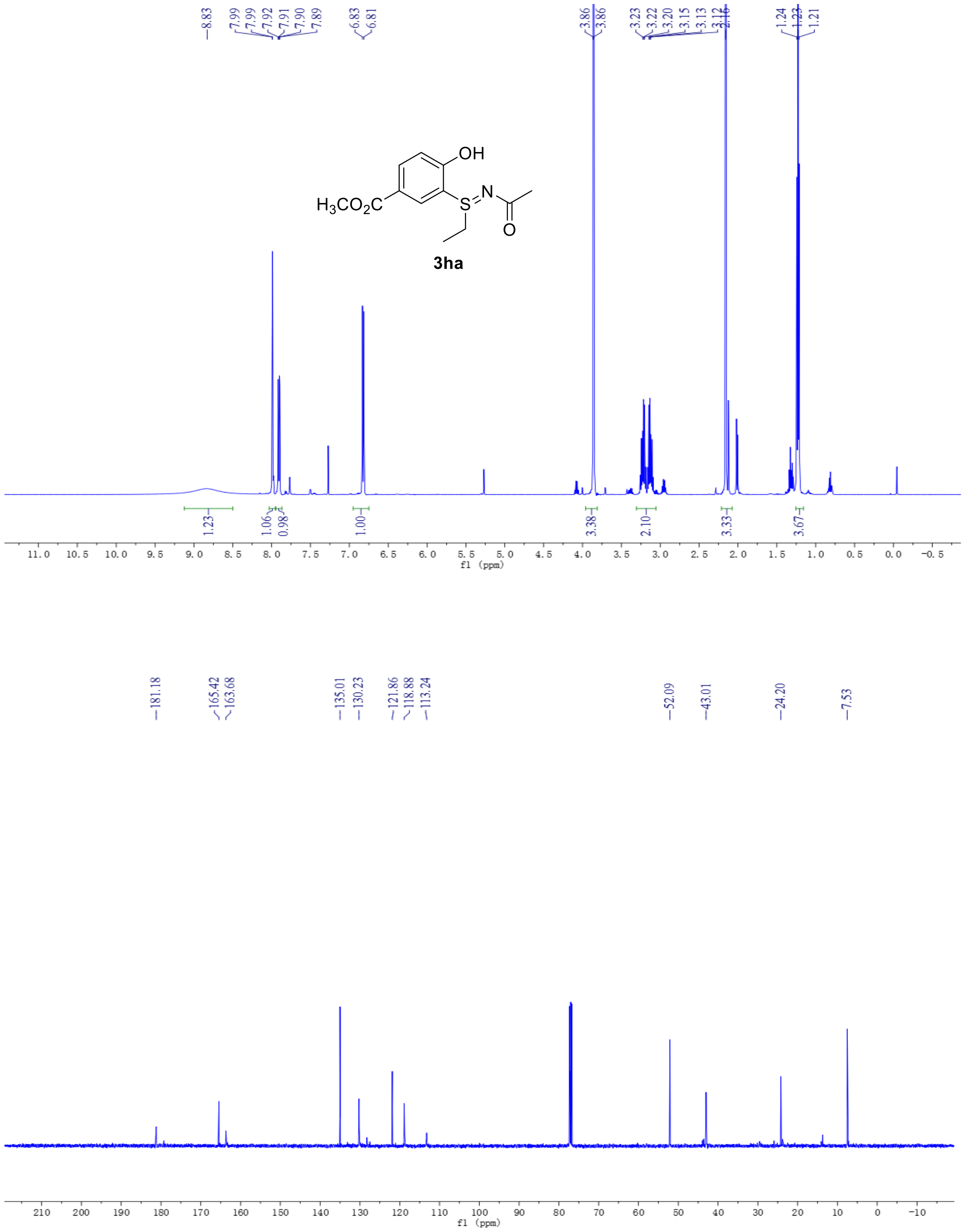

Supplementary Figure 9. ^{1}H and ^{13}C NMR spectra for compound **3ha**

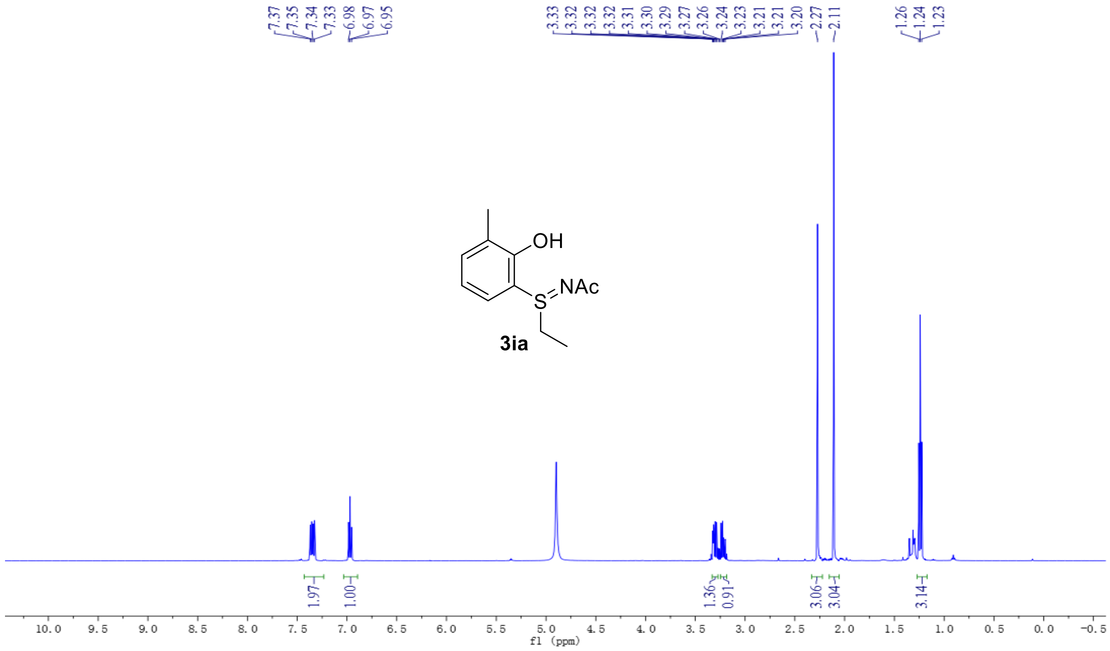

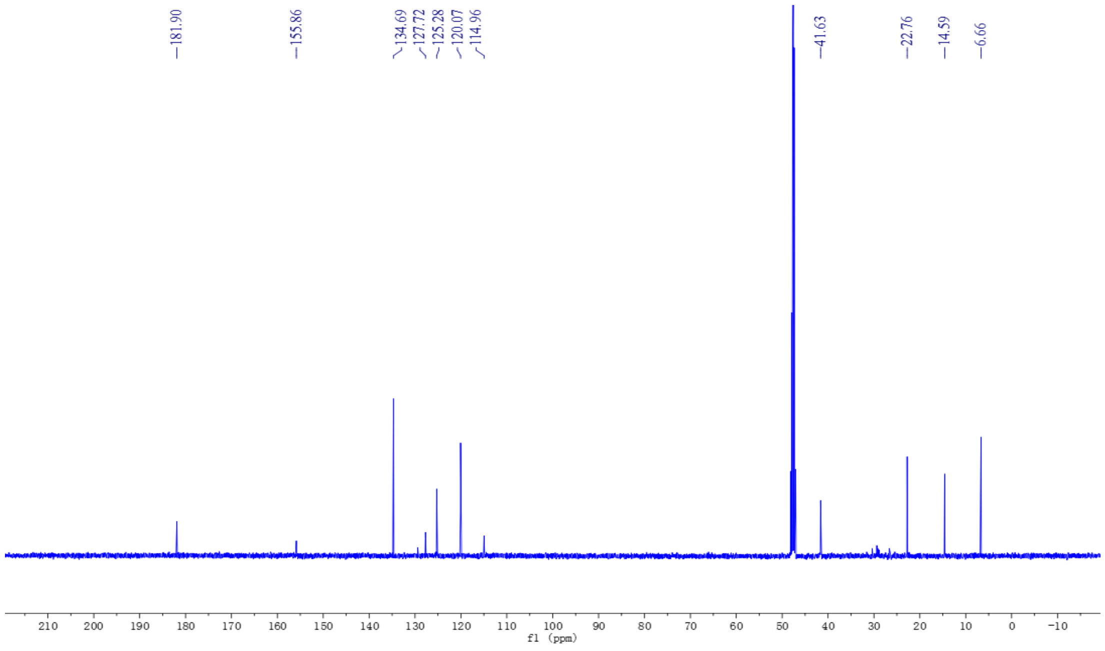

Supplementary Figure 10. ^{1}H and ^{13}C NMR spectra for compound **3ia**

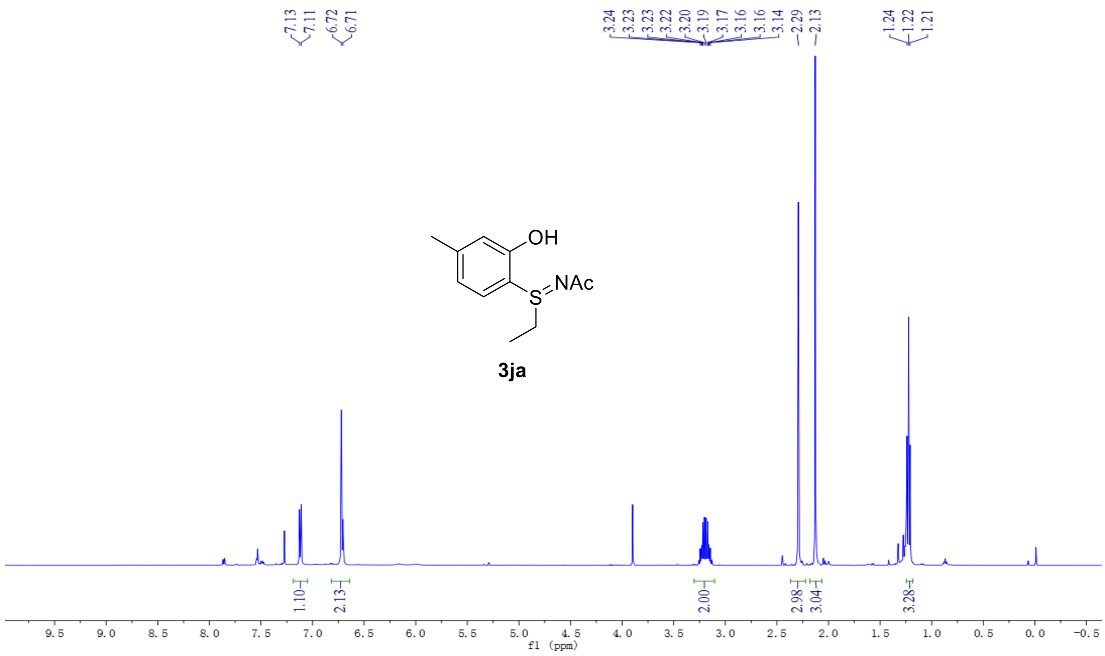

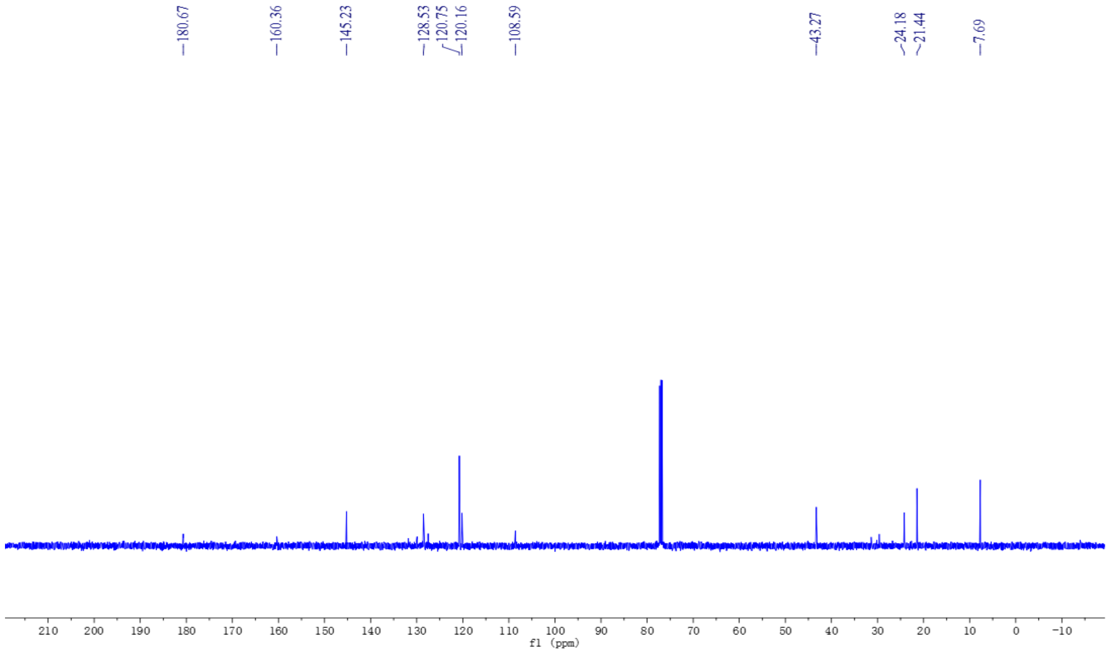

Supplementary Figure 11. ^{1}H and ^{13}C NMR spectra for compound **3ja**

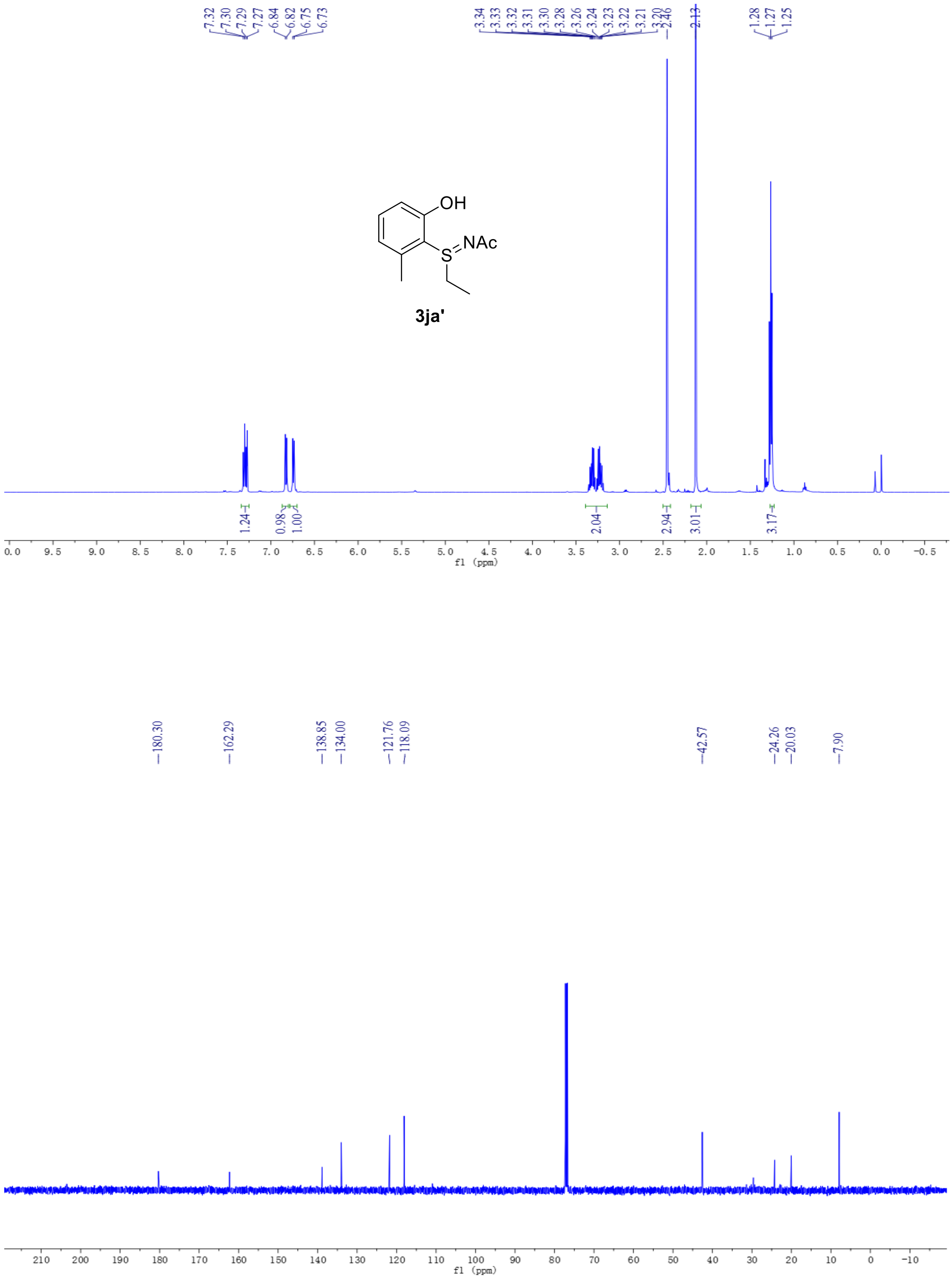

Supplementary Figure 12. ^{1}H and ^{13}C NMR spectra for compound **3ja'**

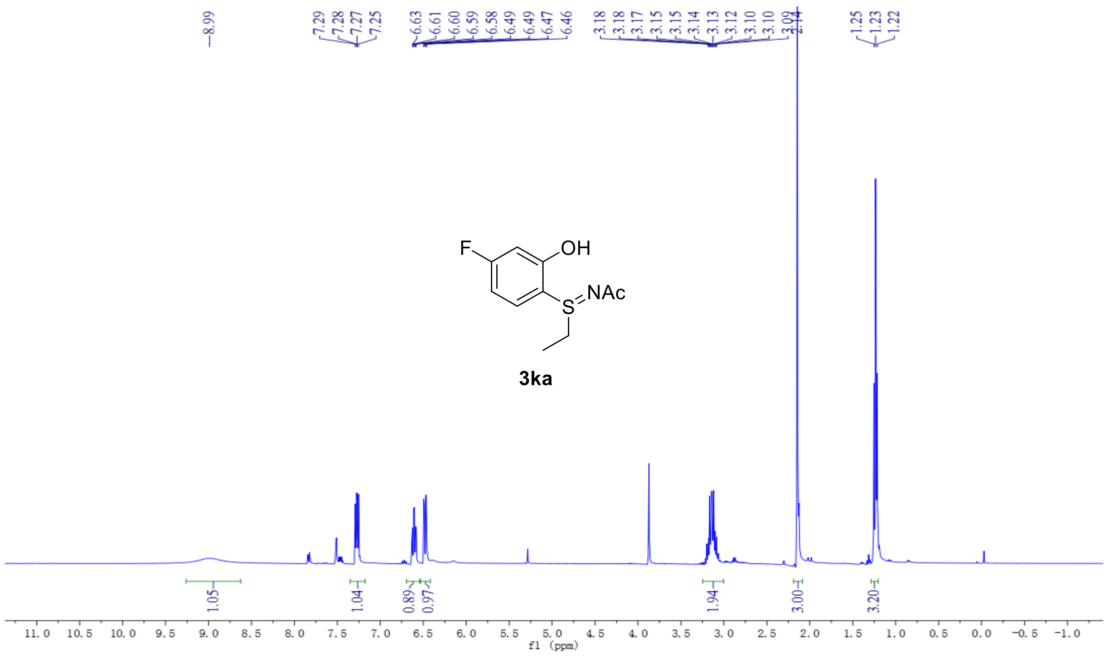

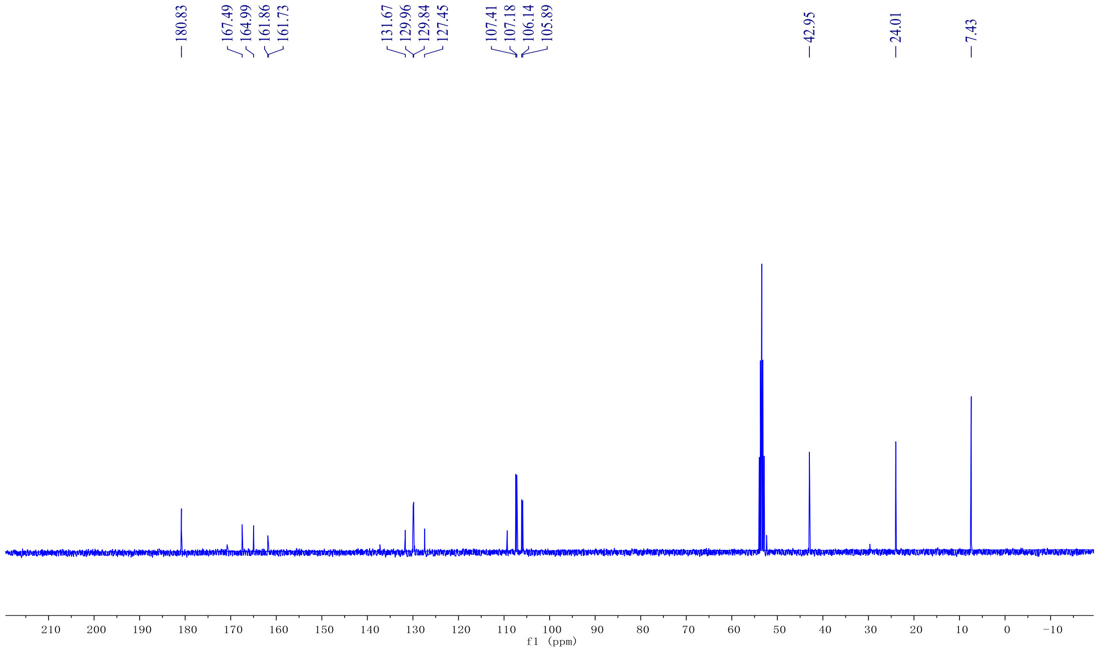

Supplementary Figure 13. ^{1}H and ^{13}C NMR spectra for compound **3ka**

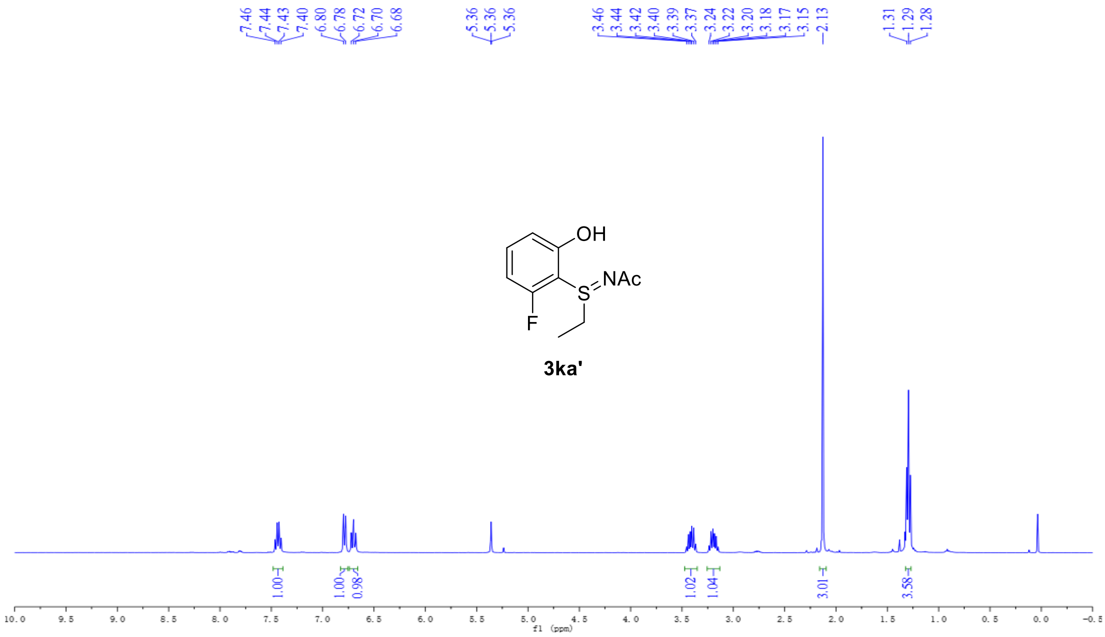

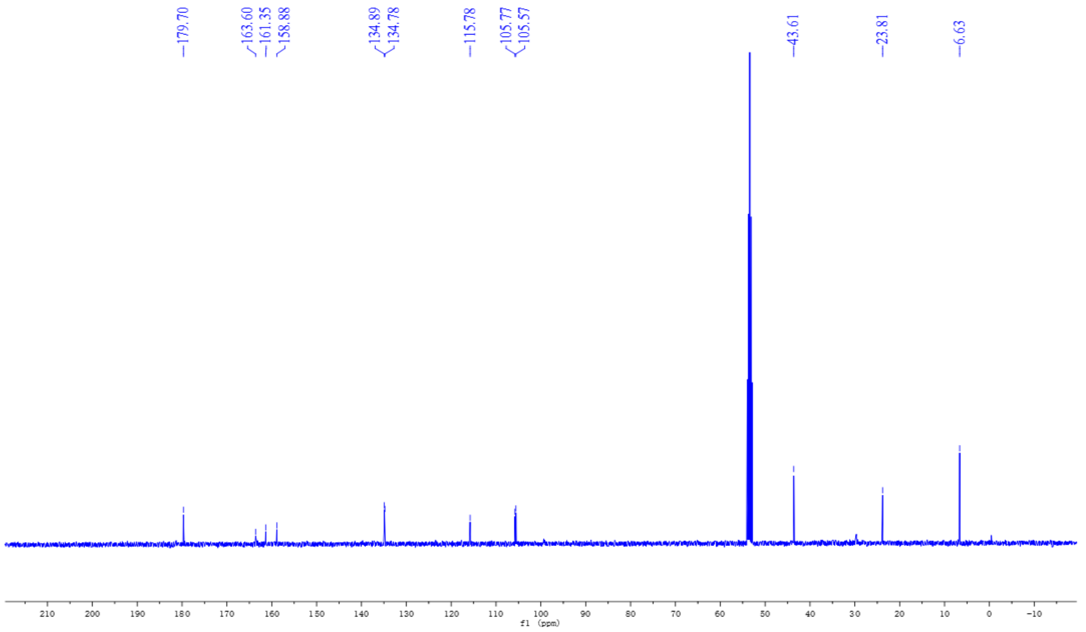

Supplementary Figure 14. ^{1}H and ^{13}C NMR spectra for compound **3ka’**

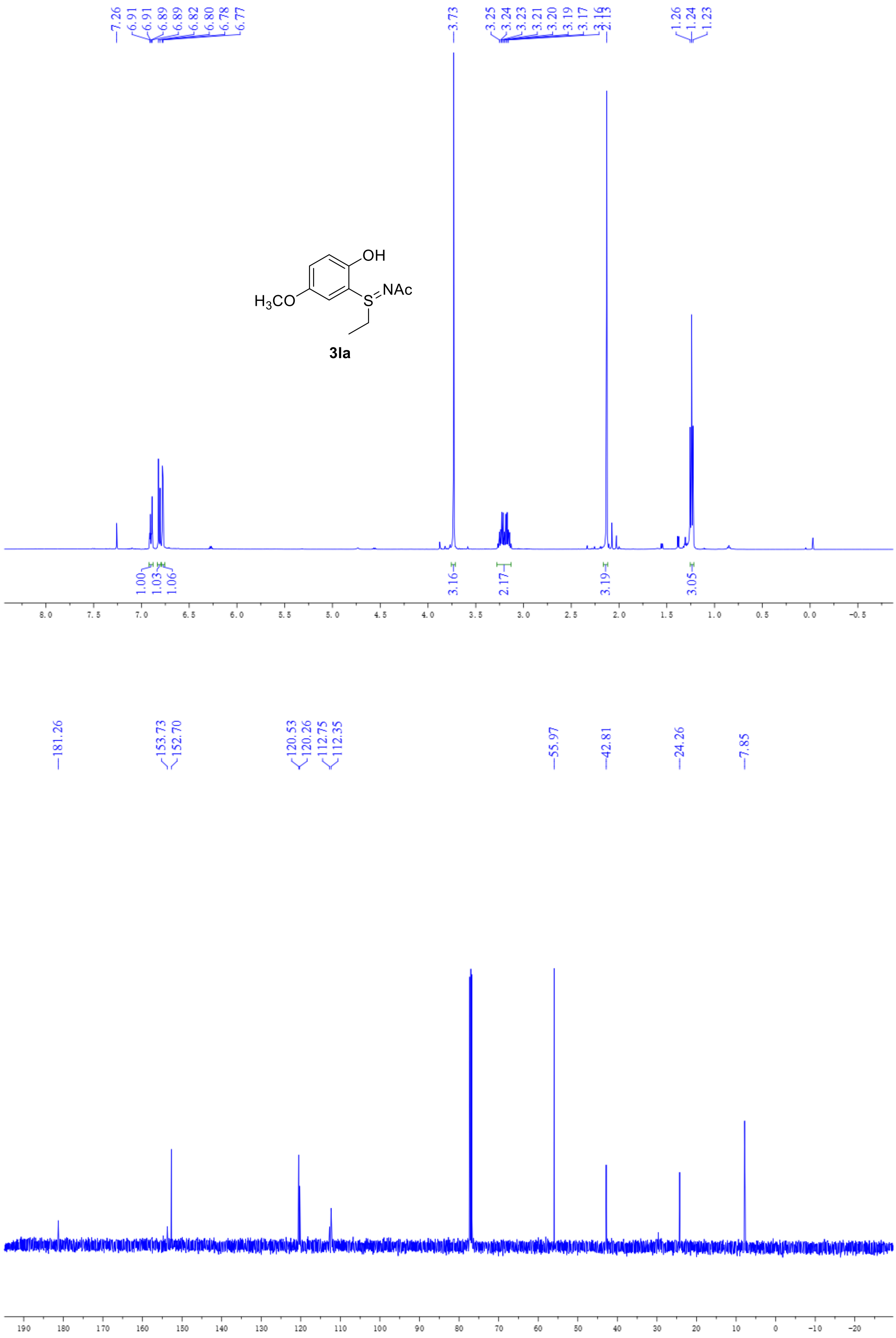

Supplementary Figure 15. ^{1}H and ^{13}C NMR spectra for compound **3la**

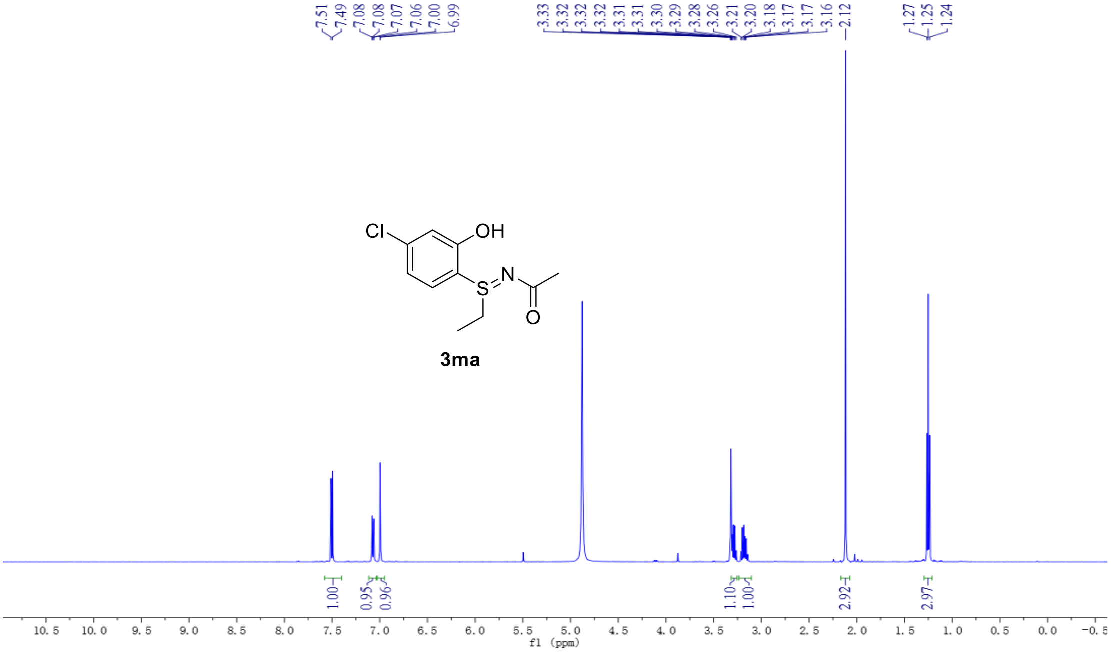

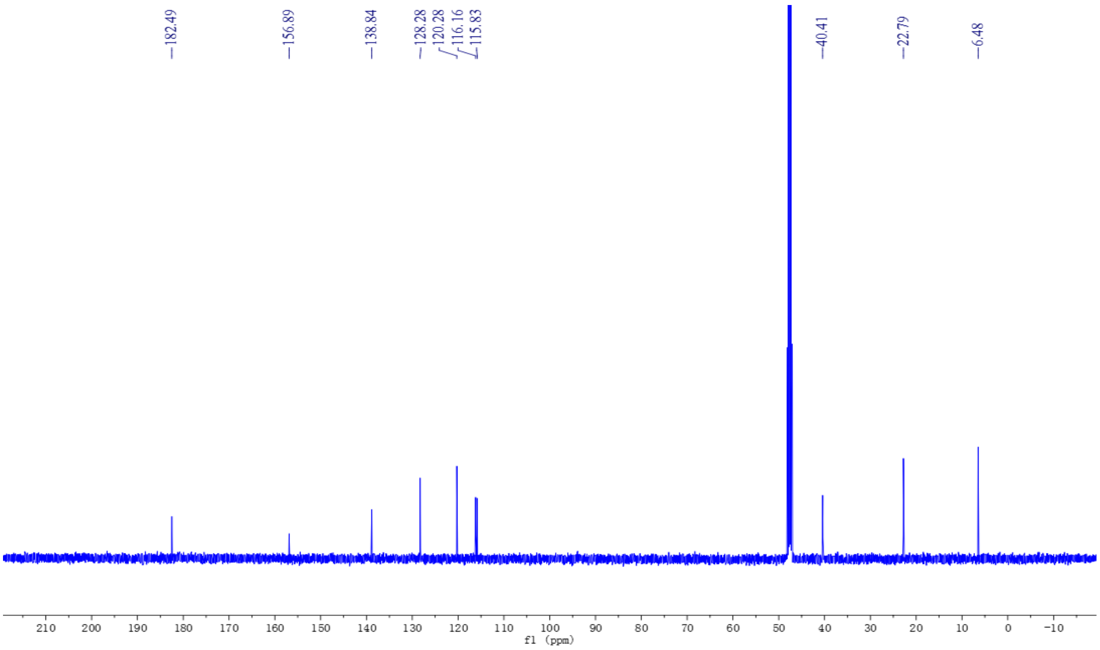

Supplementary Figure 16. ^{1}H and ^{13}C NMR spectra for compound **3ma**

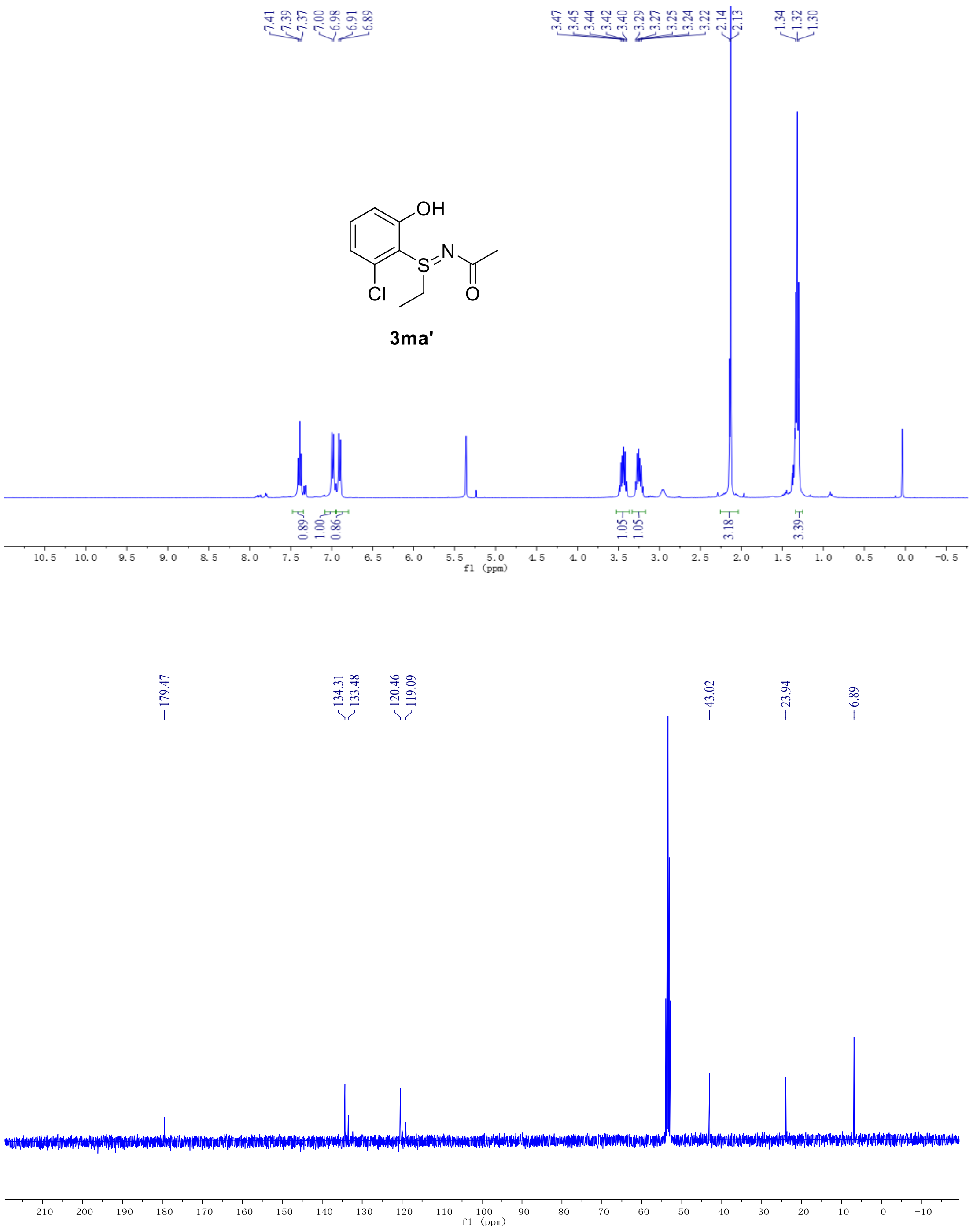

Supplementary Figure 17. ^{1}H and ^{13}C NMR spectra for compound **3ma'**

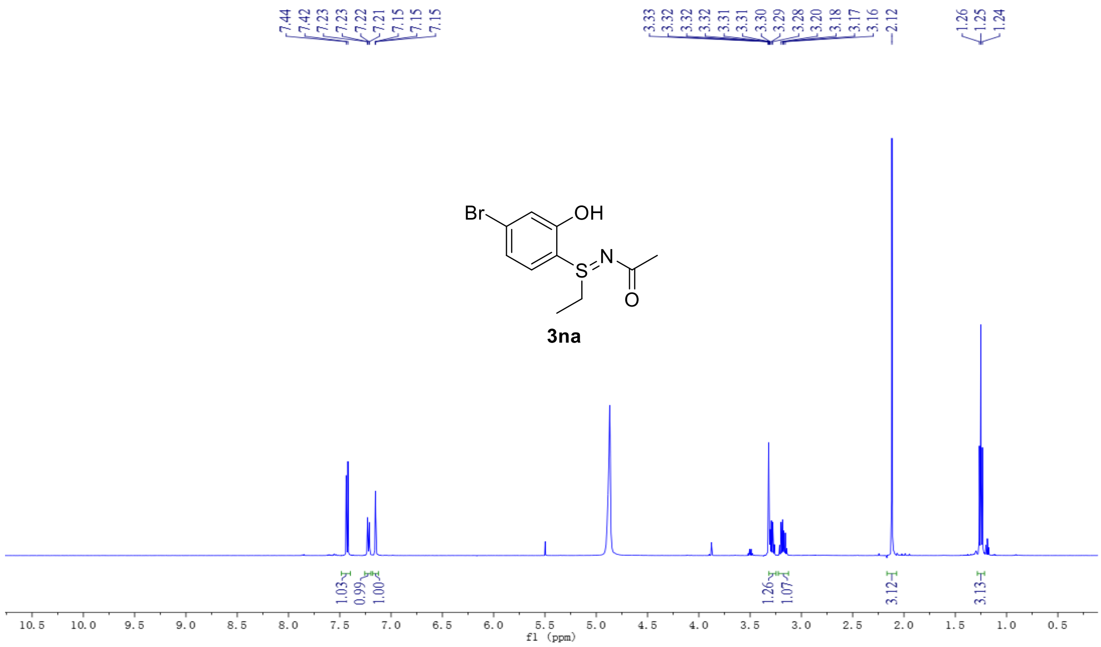

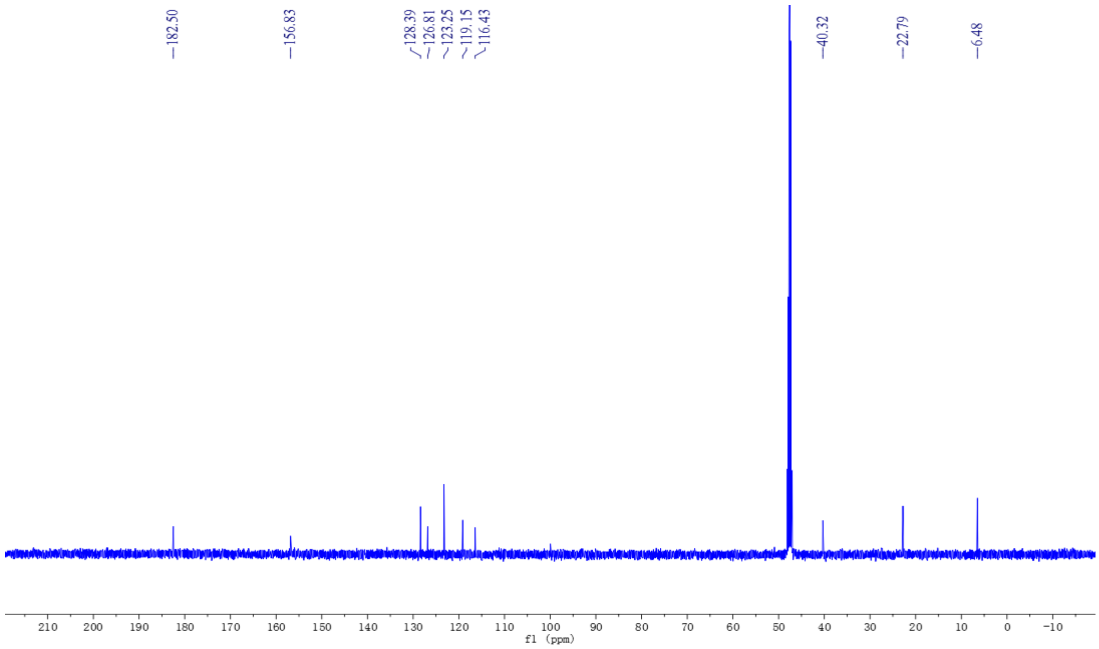

Supplementary Figure 18. ^{1}H and ^{13}C NMR spectra for compound **3na**

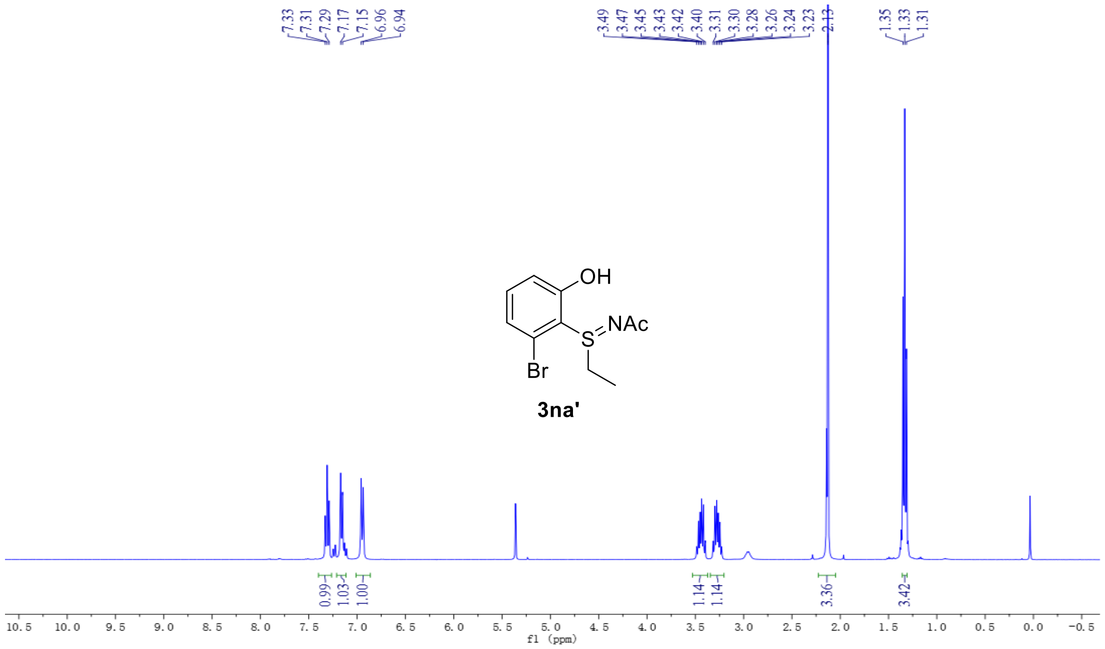

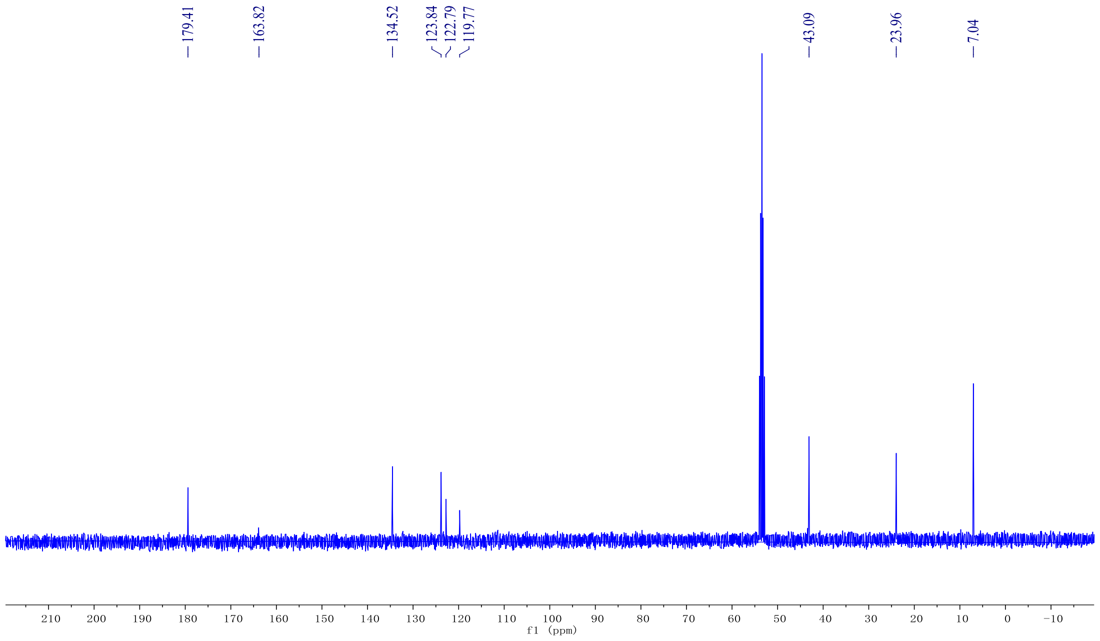

Supplementary Figure 19. ^{1}H and ^{13}C NMR spectra for compound **3na'**

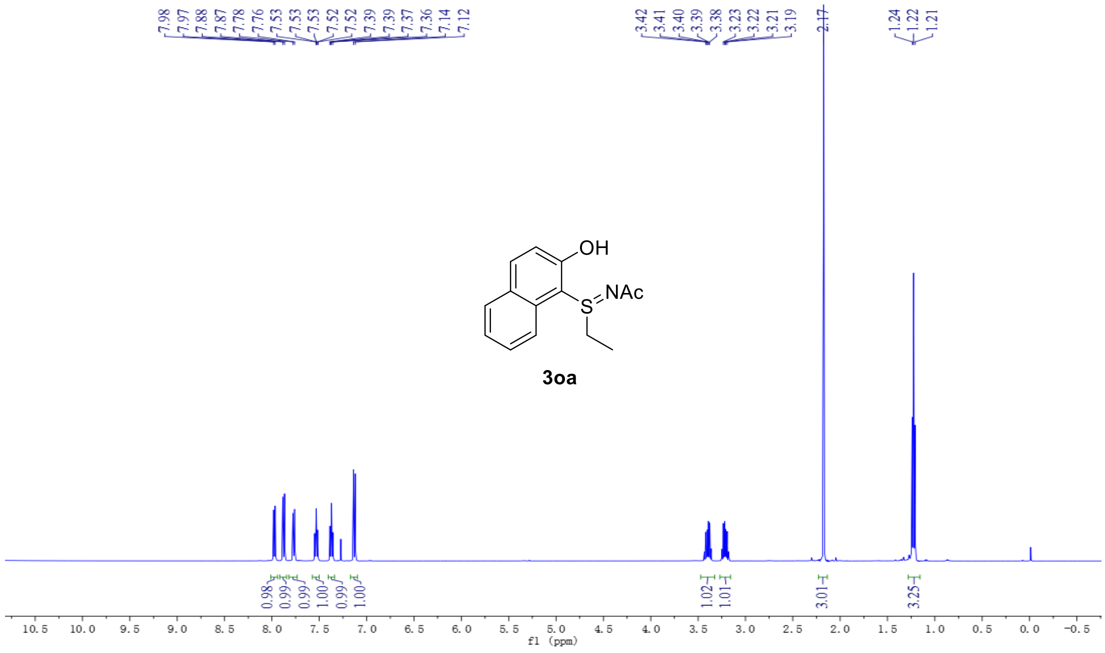

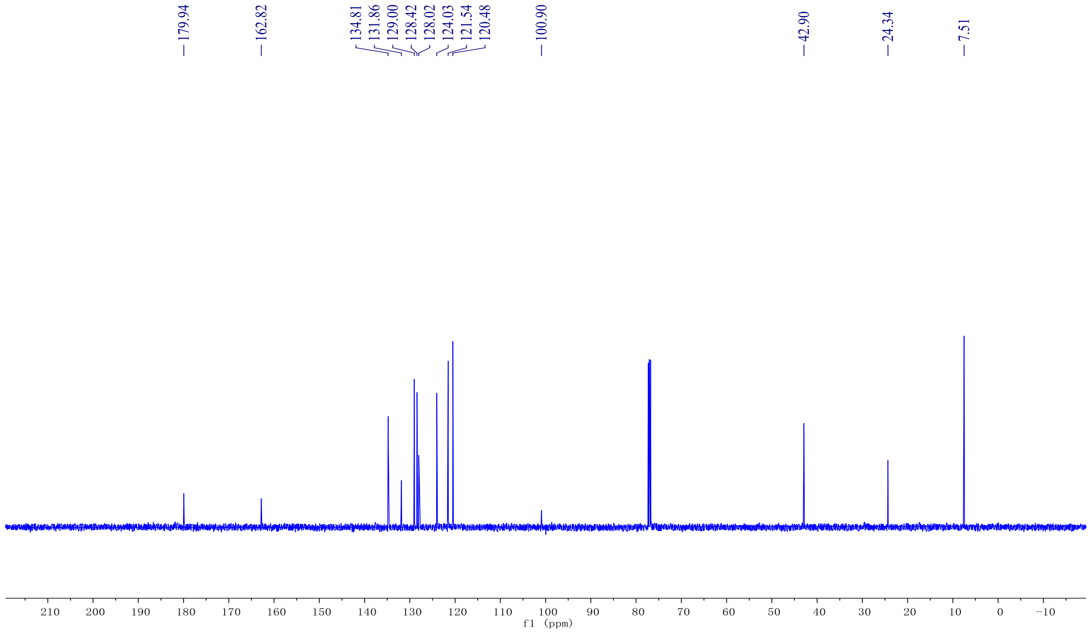

Supplementary Figure 20. ^{1}H and ^{13}C NMR spectra for compound **3oa**

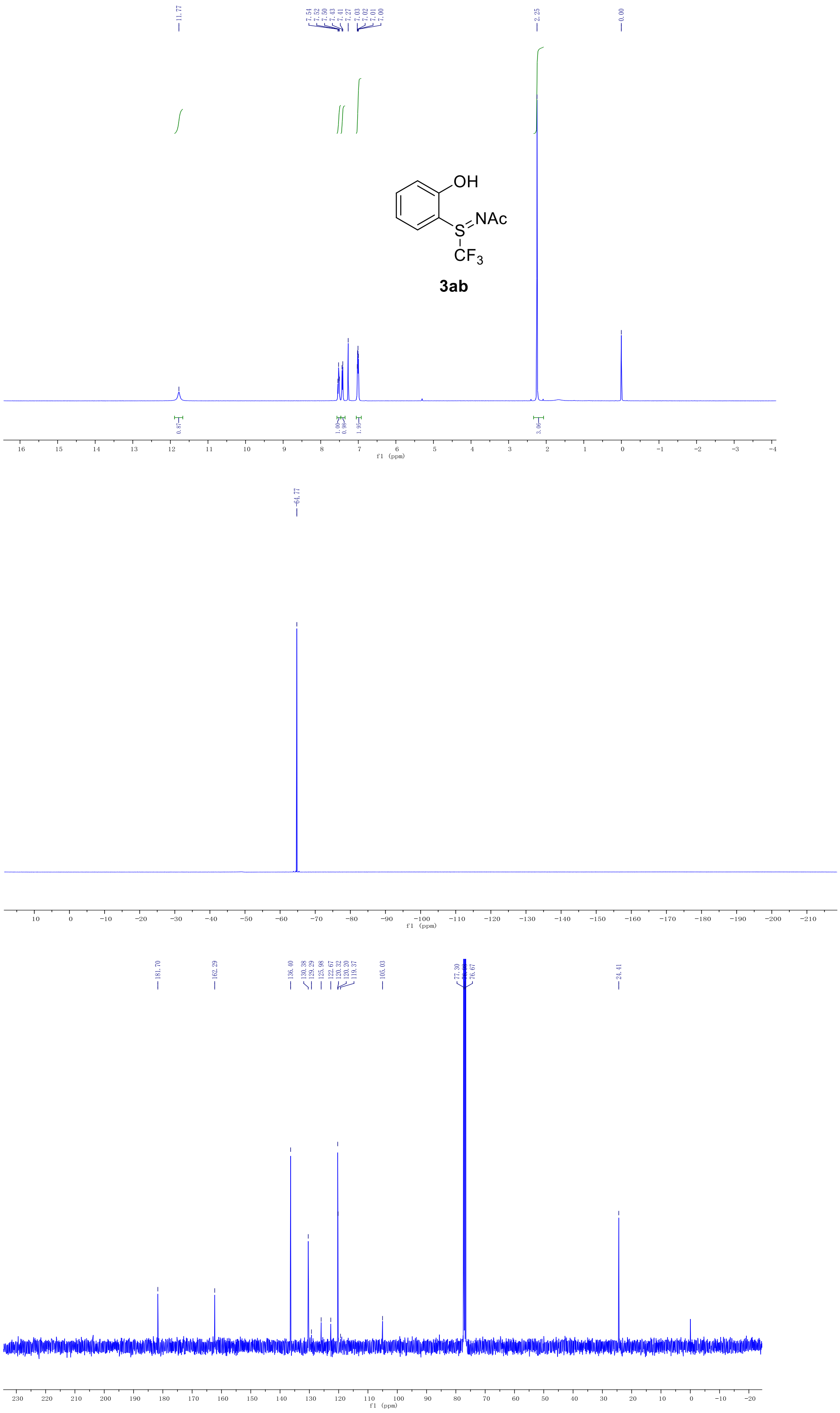

Supplementary Figure 21. ^{1}H, ^{19}F and ^{13}C NMR spectra for compound **3ab**

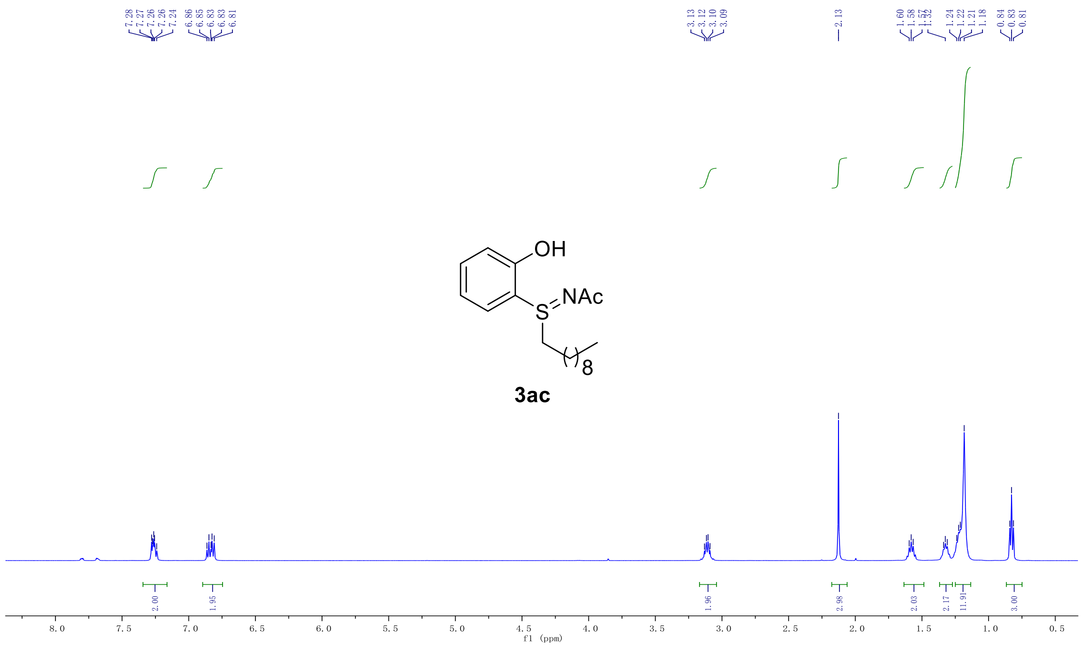

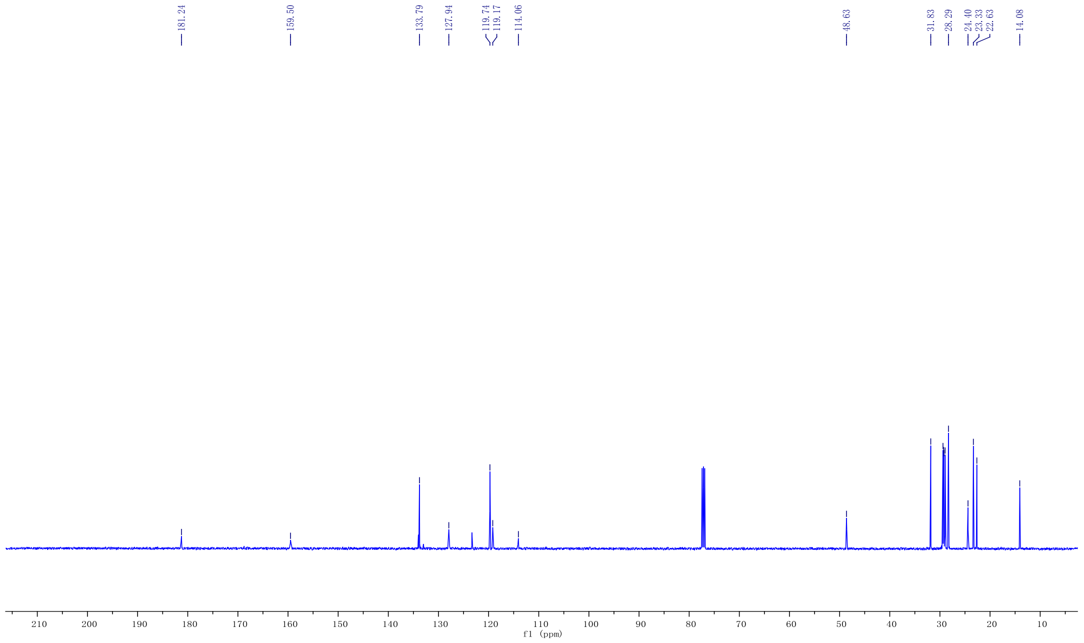

Supplementary Figure 22. ^{1}H and ^{13}C NMR spectra for compound **3ac**

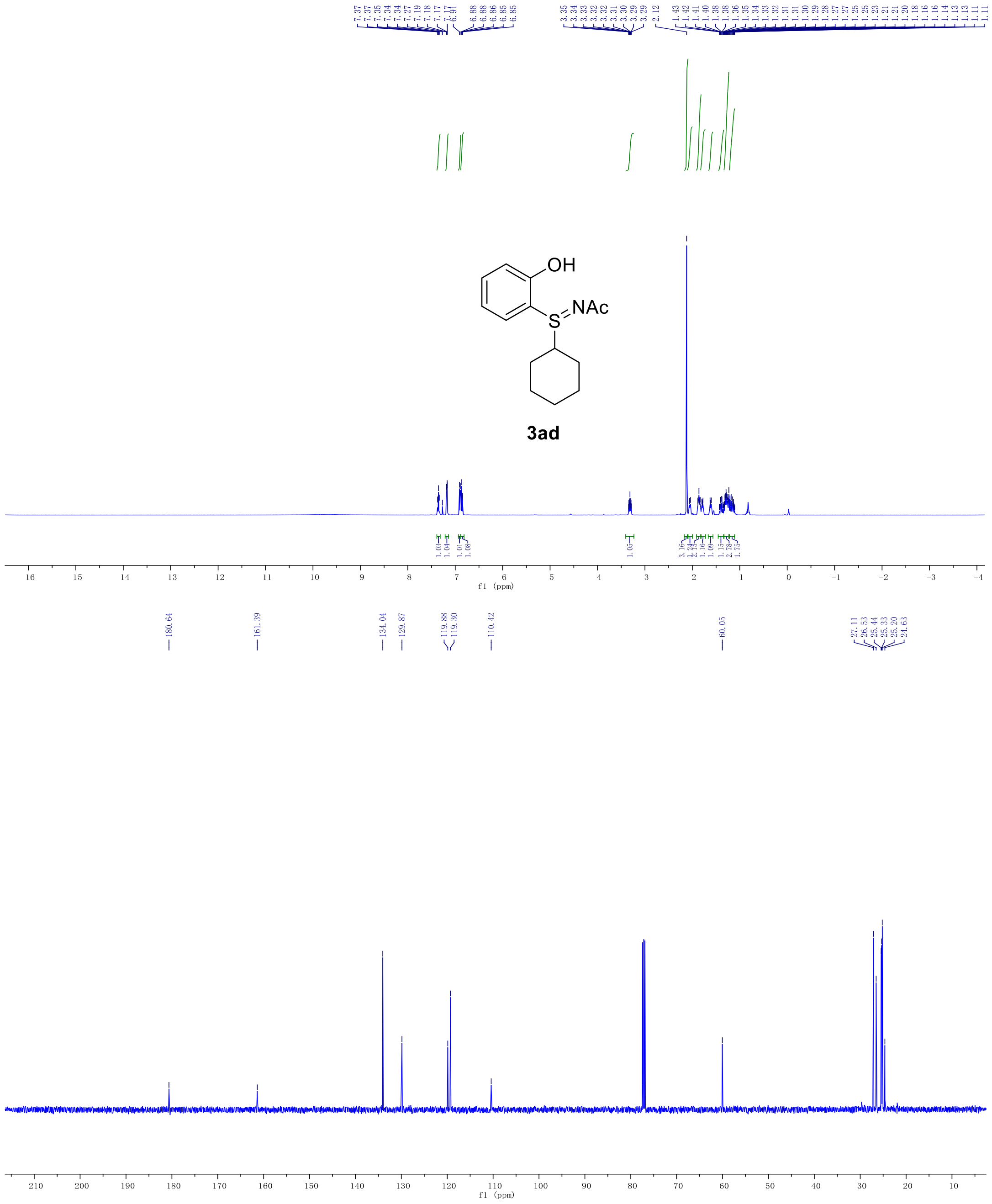

Supplementary Figure 23. ^{1}H and ^{13}C NMR spectra for compound **3ad**

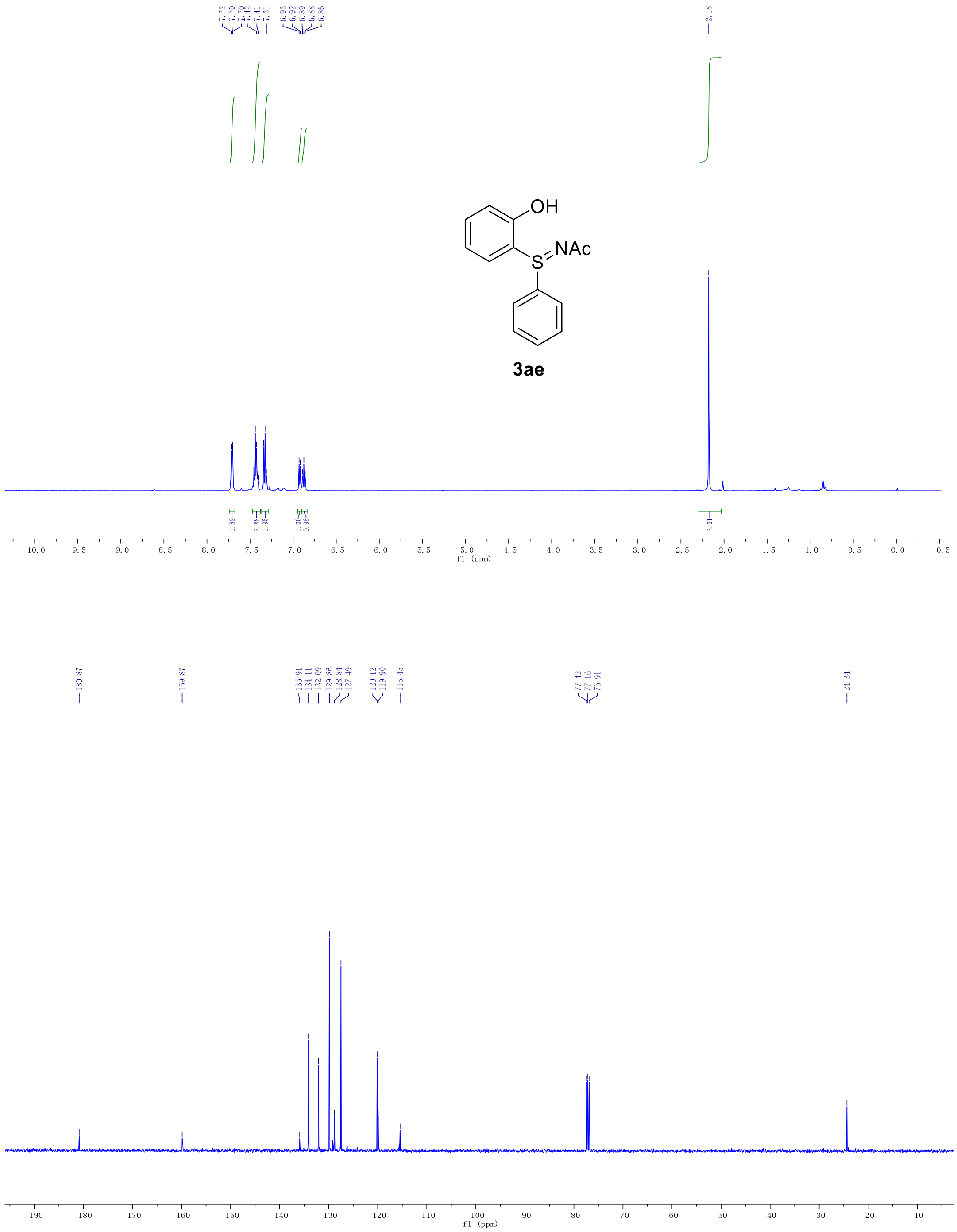

Supplementary Figure 24. ^{1}H and ^{13}C NMR spectra for compound **3ae**

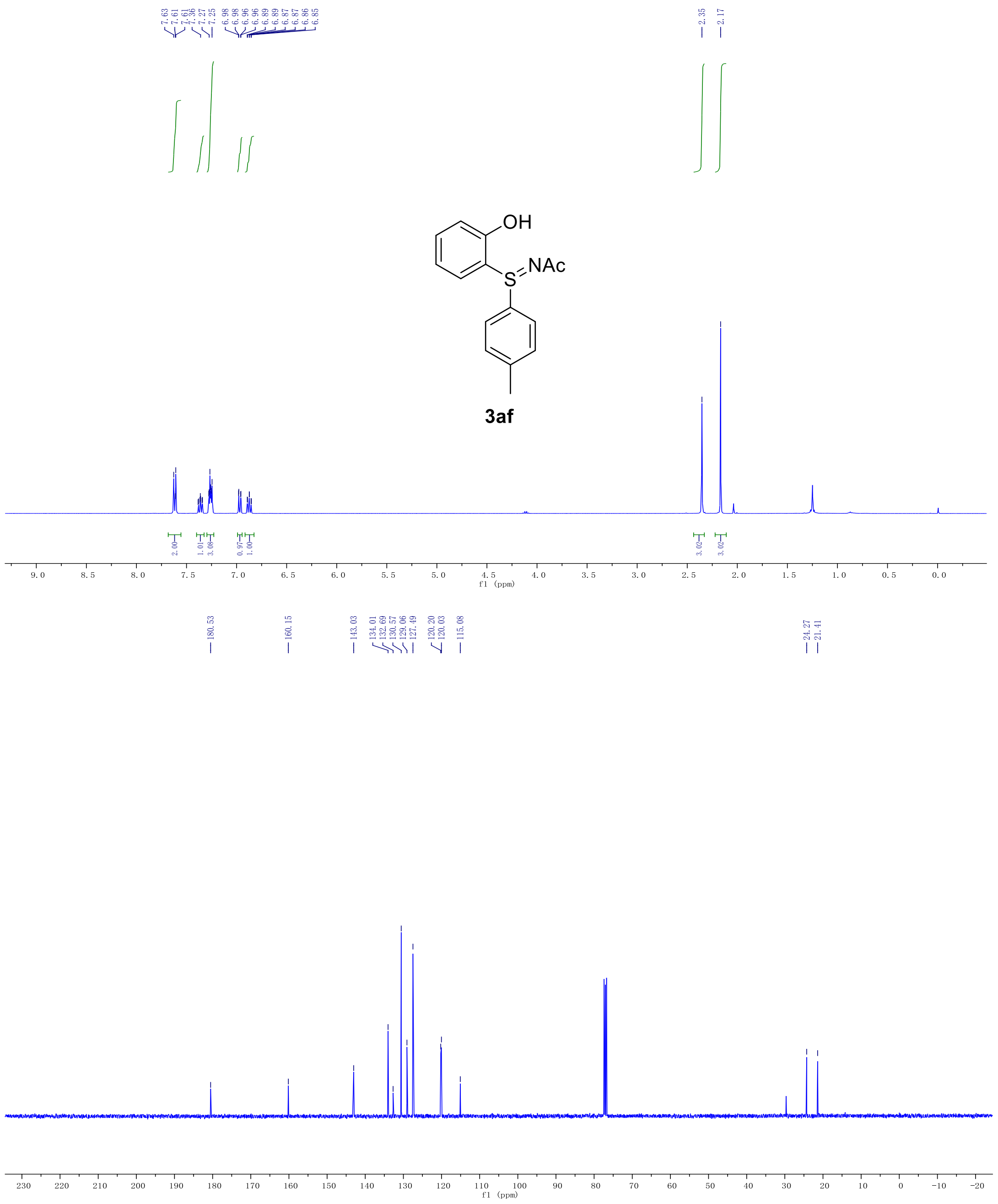

Supplementary Figure 25. ^{1}H and ^{13}C NMR spectra for compound **3af**

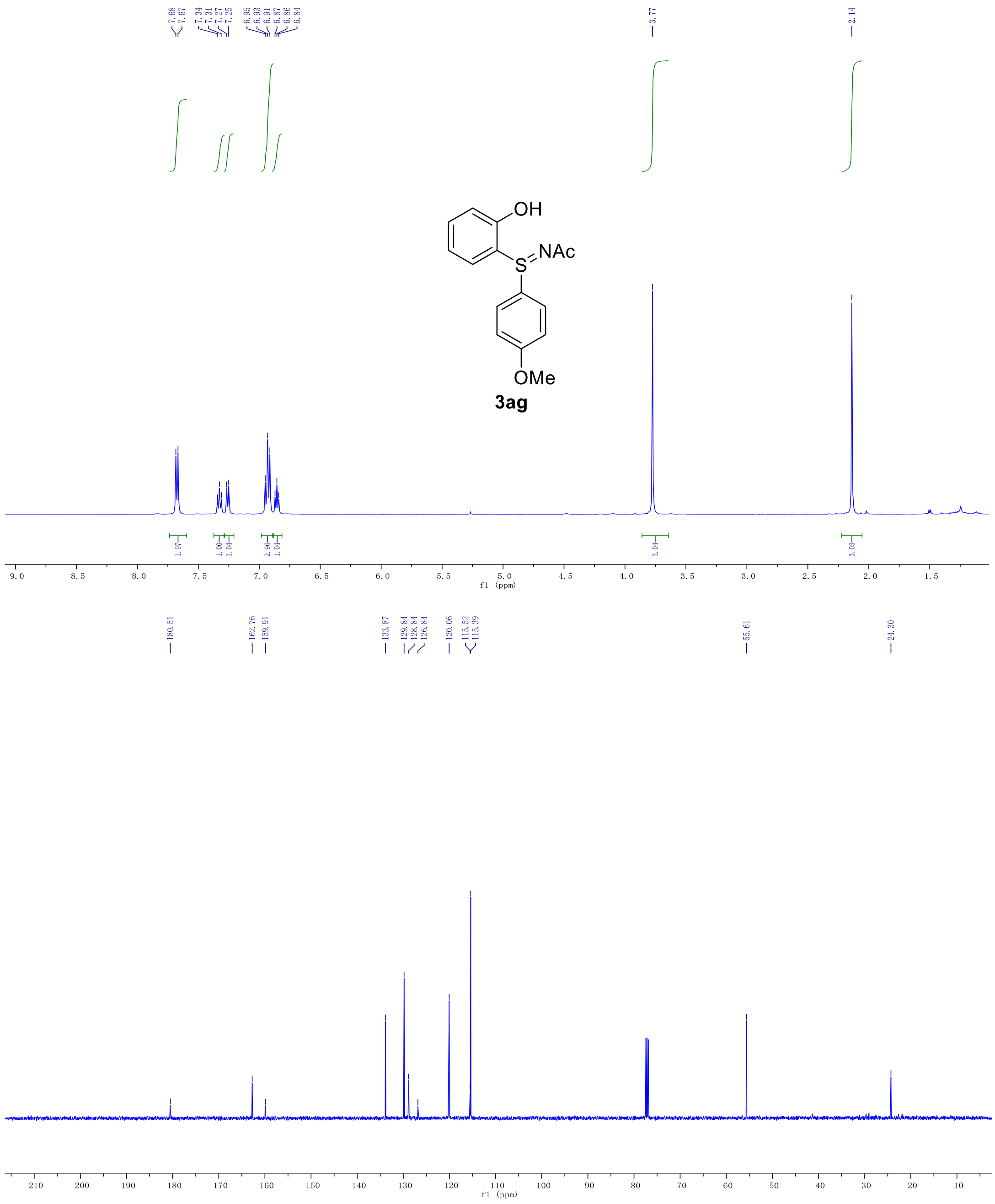

Supplementary Figure 26. ^{1}H and ^{13}C NMR spectra for compound **3ag**

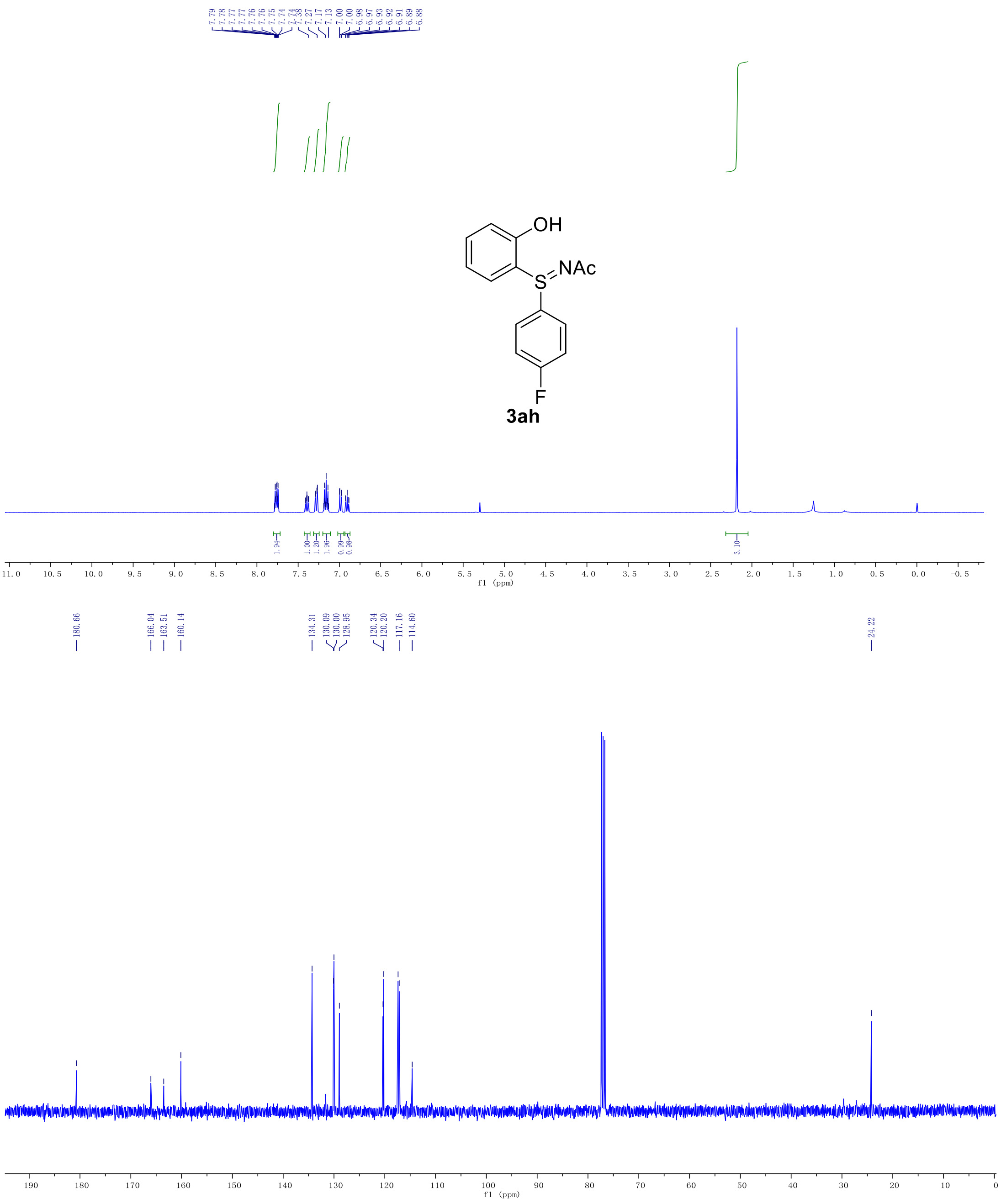

Supplementary Figure 27. ^{1}H and ^{13}C NMR spectra for compound **3ah**

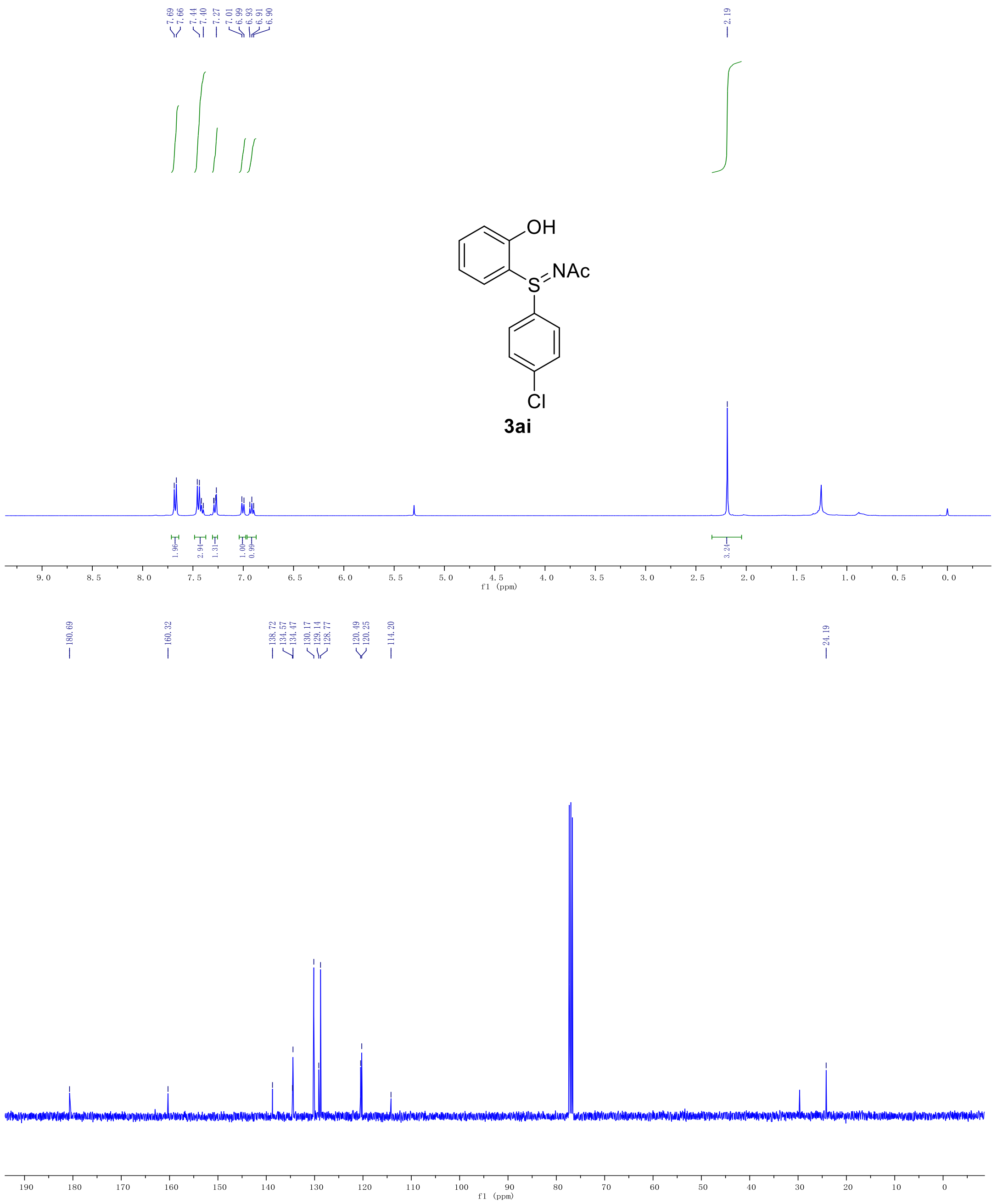

Supplementary Figure 28. ^{1}H and ^{13}C NMR spectra for compound **3ai**

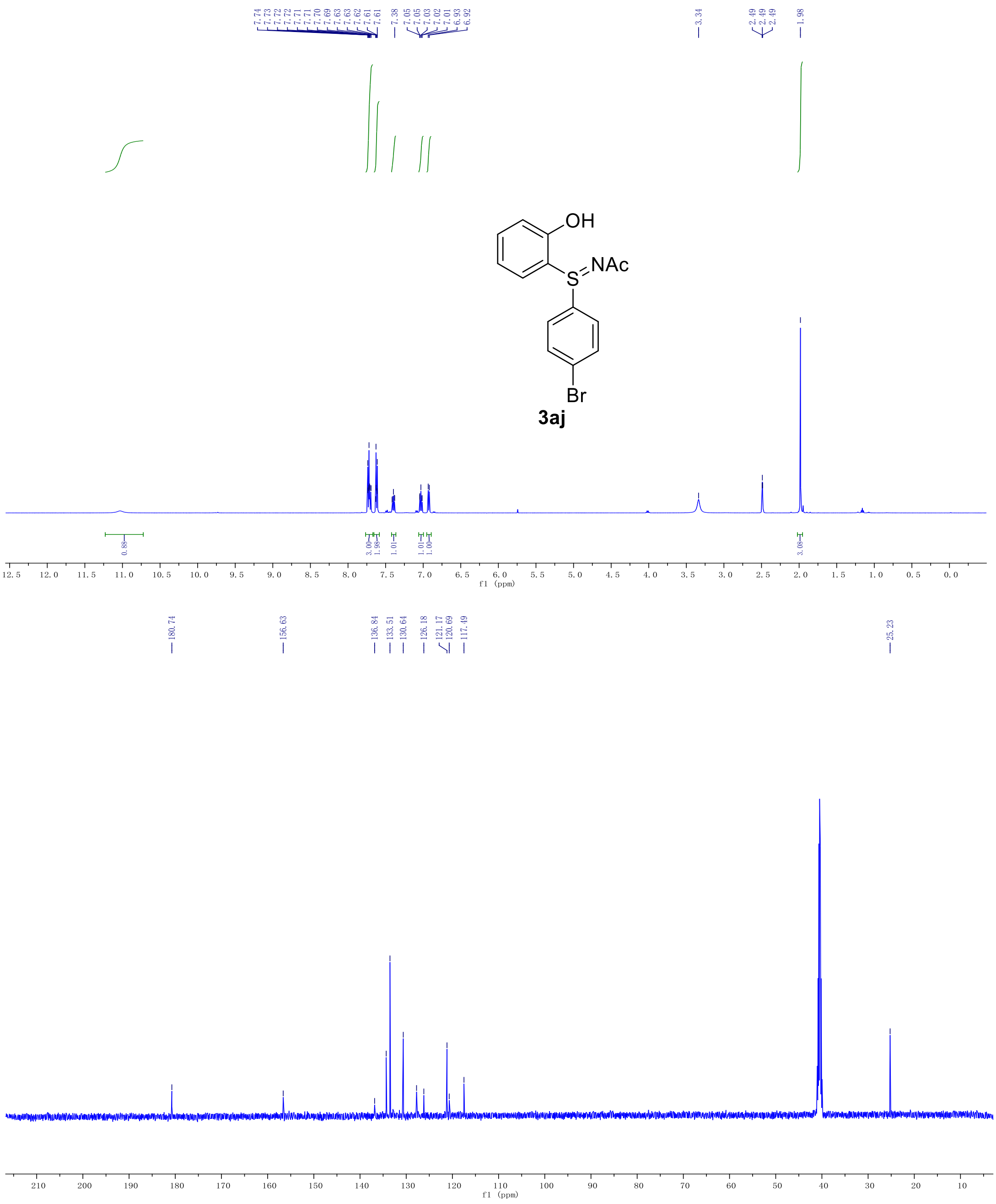

Supplementary Figure 29. ^{1}H and ^{13}C NMR spectra for compound **3aj**

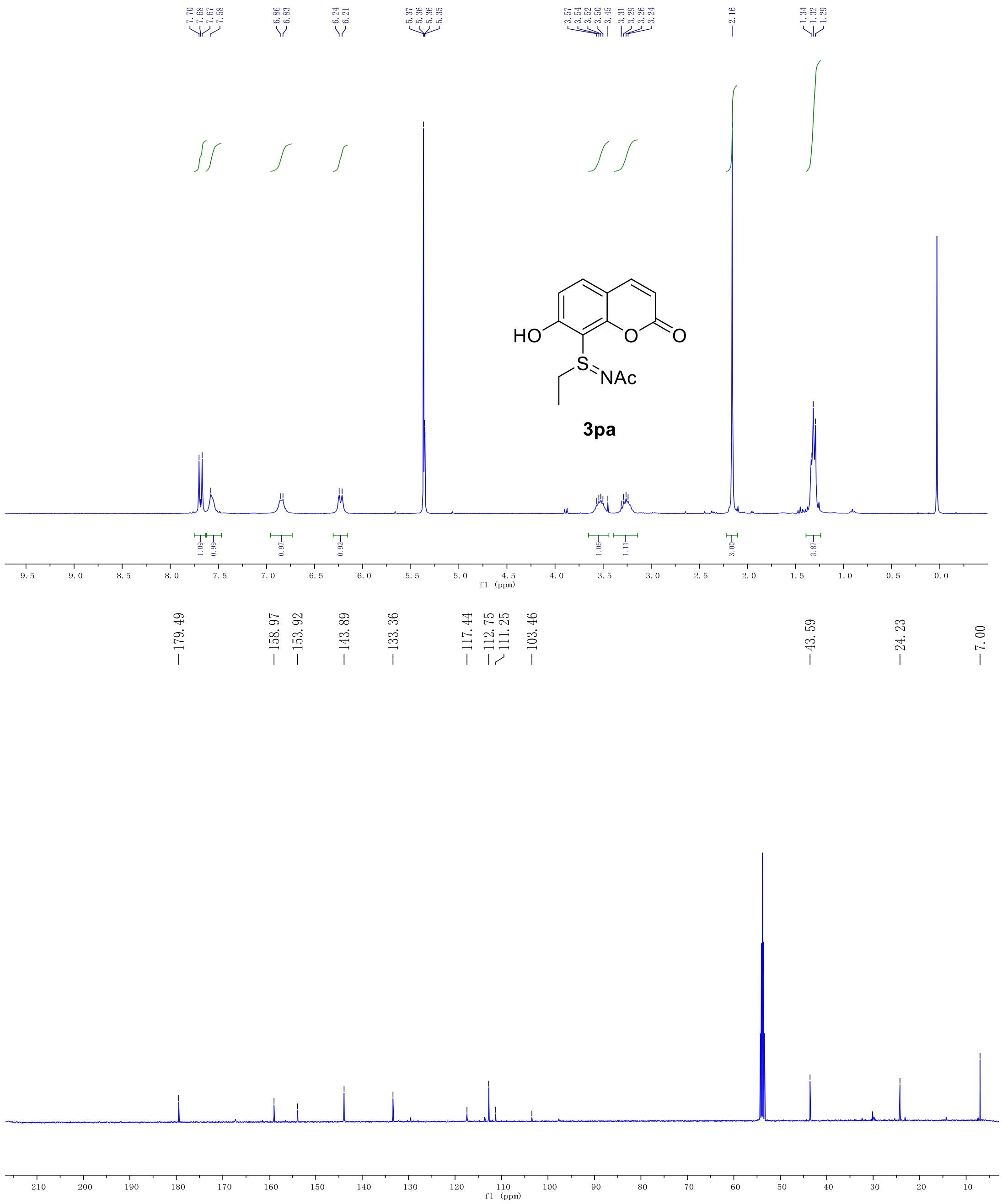

Supplementary Figure 30. ^{1}H and ^{13}C NMR spectra for compound **3pa**

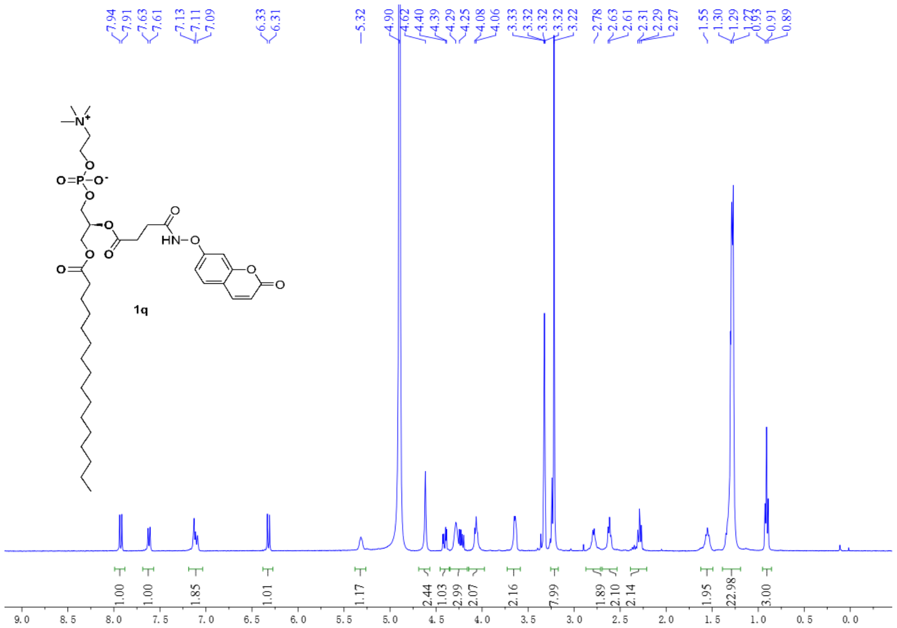

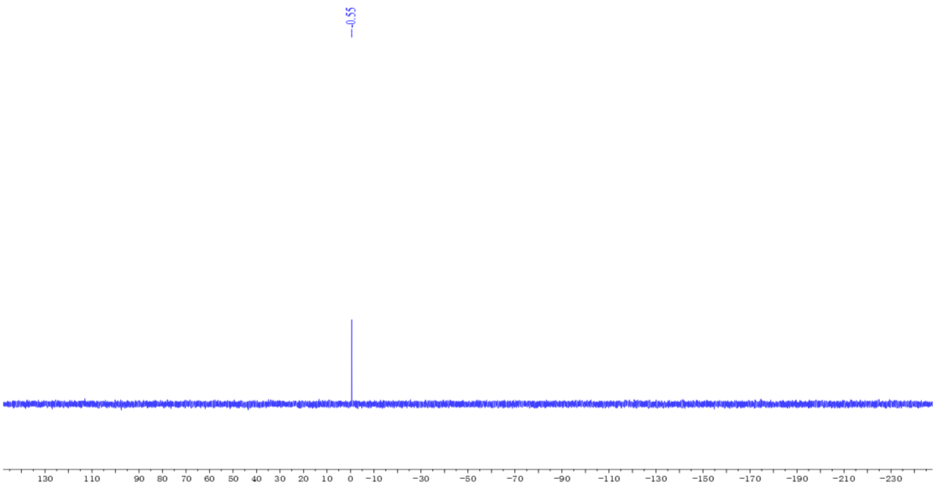

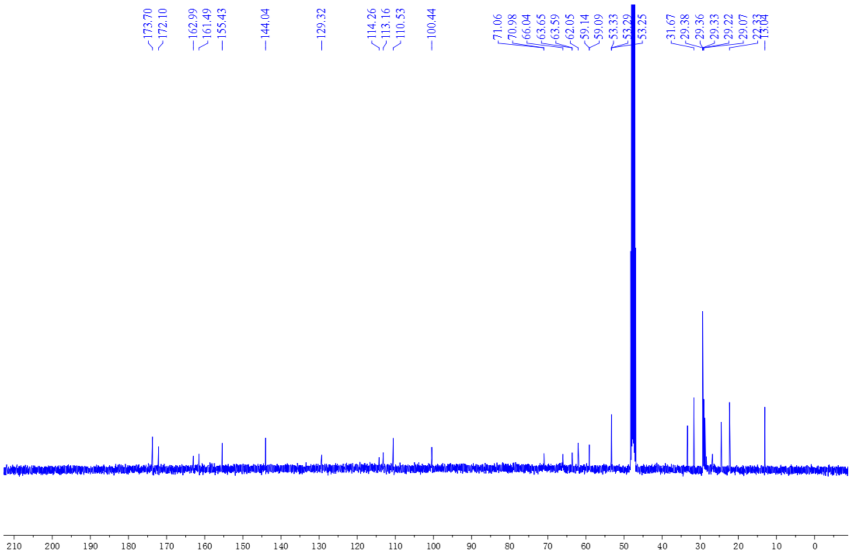

Supplementary Figure 31. ^{1}H, ^{31}P NMR and ^{13}C NMR spectra for compound **1q**

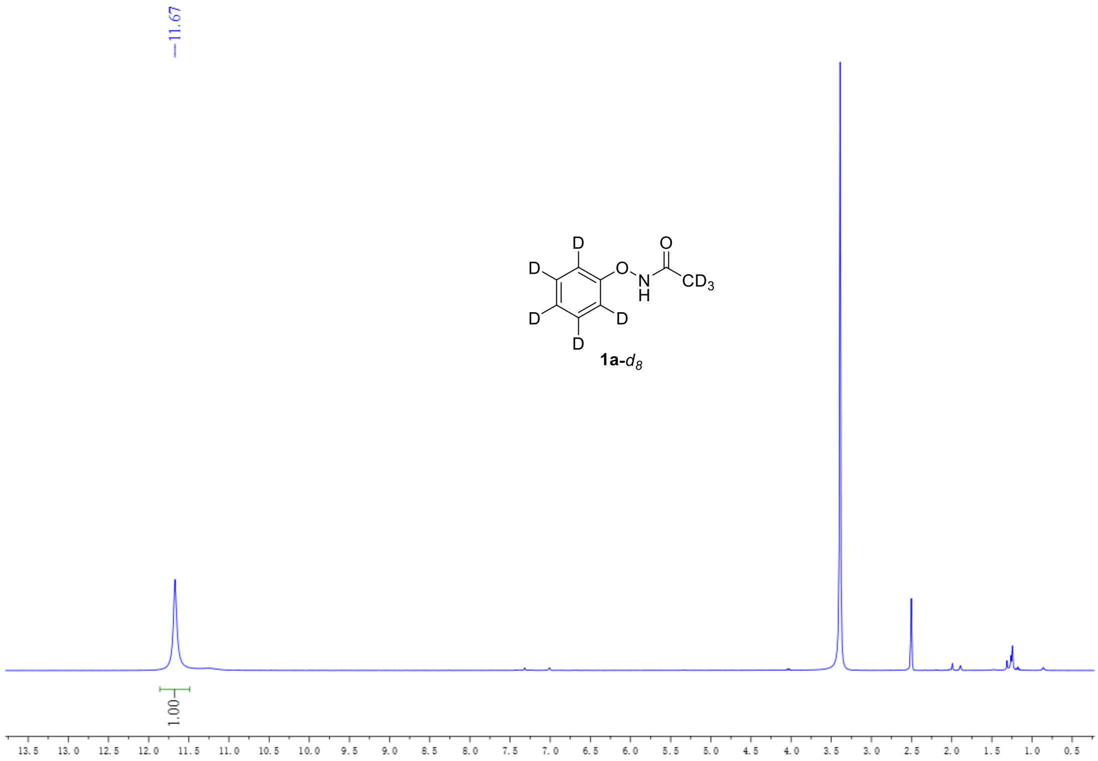

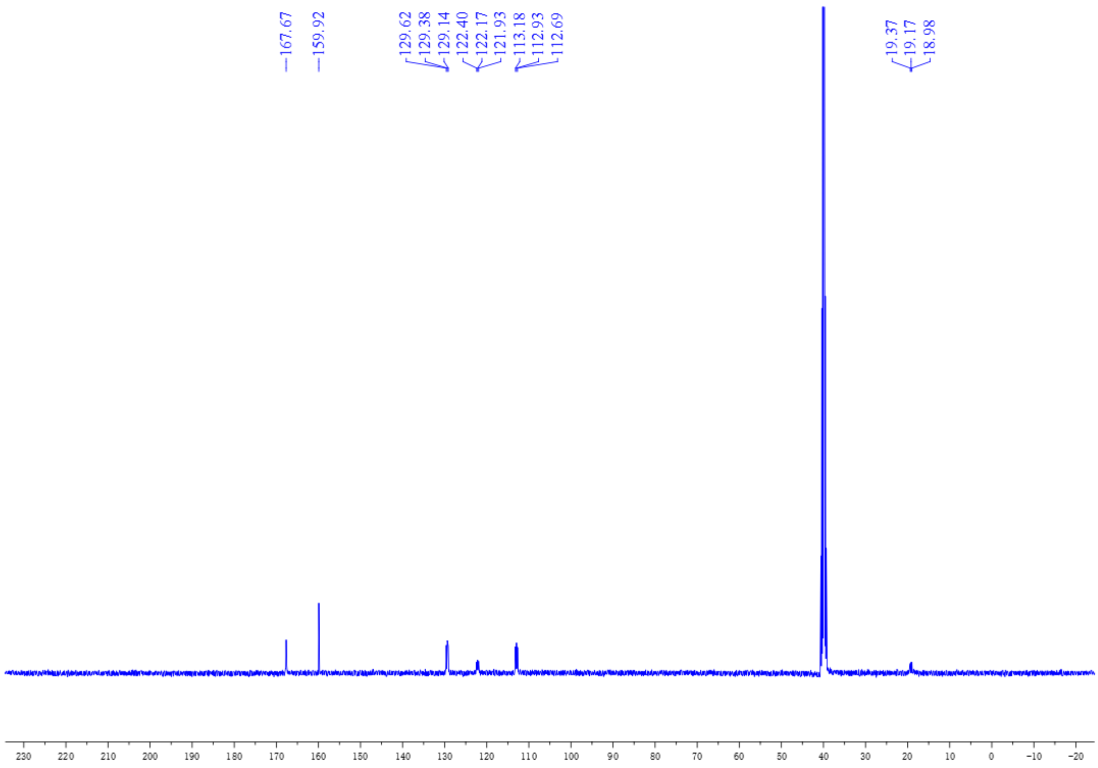

Supplementary Figure 32. ^{1}H NMR and ^{13}C NMR spectra for compound **1a-**d_8

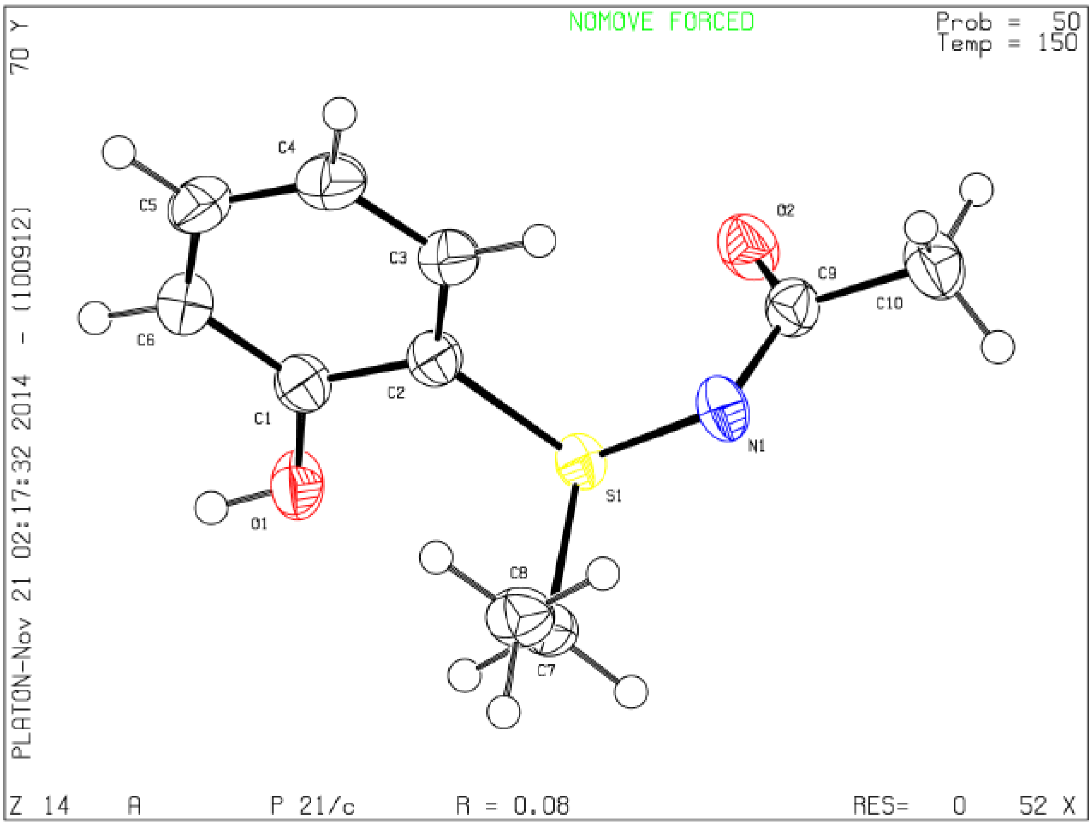

Supplementary Figure 33: X-ray diffraction structure of compound **3aa** (CCDC-1041436)**.**

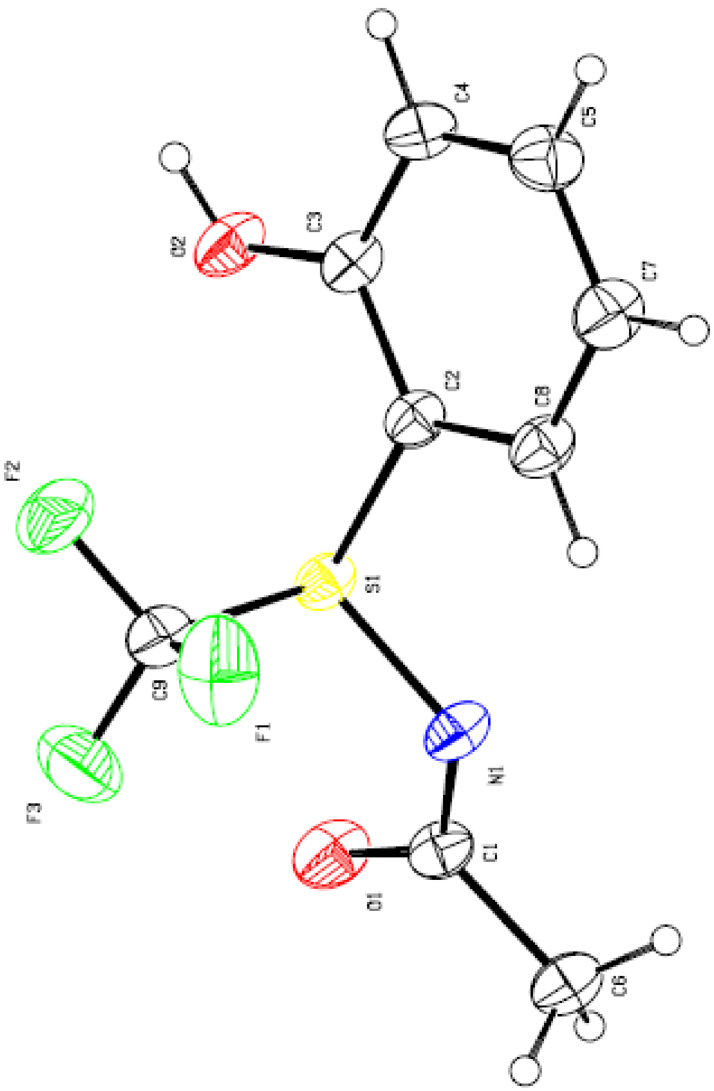

Supplementary Figure 34: X-ray diffraction structure of compound **3ab** (CCDC-983618)**.**

a

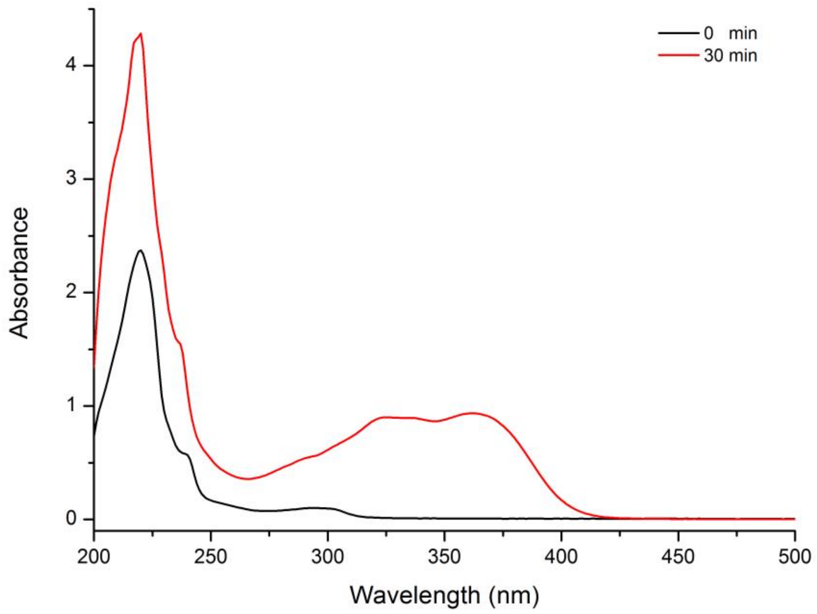

b

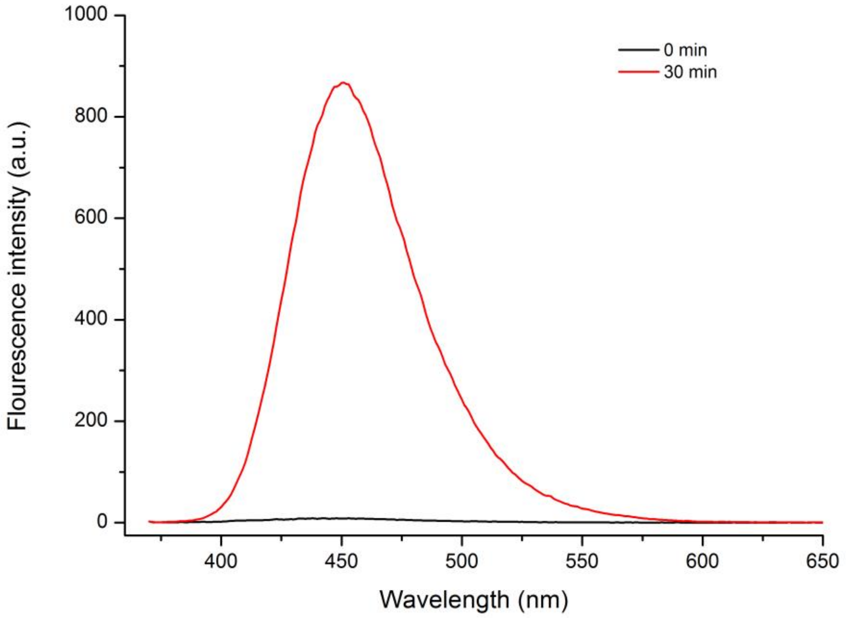

Supplementary Figure 35. **The absorption and fluorescence spectra of reaction of 1q and 2k.** (**a**) The absorption spectra of reaction of **1q** and **2k** in MeOH (2×10^{-5} mol L^{-1}). The maximum excitation wavelength of **3qk** is 362nm; (**b**) The fluorescence spectra of reaction of **1q** and **2k** in MeOH (2×10^{-5} mol L^{-1}, $\lambda_{ex} = 362$ nm). The maximum emission wavelength of **3qk** is 450nm. Under the maximum emission wavelength, the fluorescence intensity is increased 107 times in 30 minites.

Supplementary Table

Supplementary Table 1. Crystal data and structure refinement for 3aa.	
Identification code	**3aa**
Empirical formula	$C_{10}H_{13}NO_2S$
Formula weight	211.29
Temperature/K	150.15
Crystal system	monoclinic
Space group	$P2_1/c$
a/Å	9.659(13)
b/Å	13.696(18)
c/Å	8.211(10)
α/°	90
β/°	100.341(13)
γ/°	90
Volume/Å^3	1069(2)
Z	4
ρ_{calc}g/cm^3	1.3132
μ/mm^{-1}	0.277
F(000)	448.7
Crystal size/mm^3	0.21 × 0.19 × 0.11
Radiation	Mo Kα (λ = 0.71073)
2Θ range for data collection/°	6.7 to 54.9
Index ranges	-12 ≤ h ≤ 11, -17 ≤ k ≤ 15, -10 ≤ l ≤ 10
Reflections collected	8159
Independent reflections	2435 [R_{int} = 0.1291, R_{sigma} = 0.1748]
Data/restraints/parameters	2435/0/126
Goodness-of-fit on F^2	0.891
Final R indexes [I>=2σ (I)]	R_1 = 0.0741, wR_2 = 0.1195
Final R indexes [all data]	R_1 = 0.1049, wR_2 = 0.1273
Largest diff. peak/hole / e Å^{-3}	1.29/-1.35

Supplementary Table 2. Bond Lengths for 3aa.					
**Atom**	**Atom**	**Length/Å**	**Atom**	**Atom**	**Length/Å**
S1	N1	1.671(2)	C1	C6	1.399(4)
S1	C2	1.780(3)	C2	C3	1.390(4)
S1	C7	1.806(3)	C3	C4	1.383(4)
O1	C1	1.354(3)	C4	C5	1.400(4)
O2	C9	1.256(3)	C5	C6	1.384(4)
N1	C9	1.340(3)	C7	C8	1.514(3)
C1	C2	1.401(3)	C9	C10	1.507(3)

Supplementary Table 3. Bond Angles for 3aa.							
**Atom**	**Atom**	**Atom**	**Angle/°**	**Atom**	**Atom**	**Atom**	**Angle/°**
C2	S1	N1	106.29(11)	C3	C2	C1	121.1(2)
C7	S1	N1	98.94(11)	C4	C3	C2	120.1(2)
C7	S1	C2	100.79(11)	C5	C4	C3	119.2(3)
C9	N1	S1	109.71(15)	C6	C5	C4	120.9(3)
C2	C1	O1	117.5(2)	C5	C6	C1	120.1(2)
C6	C1	O1	124.0(2)	C8	C7	S1	115.0(2)
C6	C1	C2	118.5(2)	N1	C9	O2	125.2(2)
C1	C2	S1	116.4(2)	C10	C9	O2	119.5(2)
C3	C2	S1	122.50(17)	C10	C9	N1	115.3(2)

Supplementary Table 4. Crystal data and structure refinement for 3ab.

Identification code	**3ab**	
Empirical formula	$C_9H_8F_3NO_2S$	
Formula weight	251.22	
Temperature	295(2) K	
Wavelength	1.54178 Å	
Crystal system	Orthorhombic	
Space group	P b c a	
Unit cell dimensions	a = 14.0598(7) Å	α= 90 °.
b = 7.5447(2) Å	β= 90 °.	

c = 19.6121(14) Å	γ = 90 °.
Volume	2080.39(19) $Å^3$
Z	8
Density (calculated)	1.604 Mg/m^3
Absorption coefficient	3.099 mm^{-1}
F(000)	1024
Crystal size	0.20 x 0.05 x 0.05 mm^3
Theta range for data collection	7.03 to 68.24 °.
Index ranges	-16<=h<=16, -9<=k<=8, -23<=l<=21
Reflections collected	14332
Independent reflections	1879 [R(int) = 0.0446]
Completeness to theta = 68.24 °	98.9 %
Absorption correction	Semi-empirical from equivalents
Max. and min. transmission	0.856 and 0.690
Refinement method	Full-matrix least-squares on F^2
Data / restraints / parameters	1879 / 1 / 150
Goodness-of-fit on F^2	1.020
Final R indices [I>2sigma(I)]	R1 = 0.0425, wR2 = 0.1242
R indices (all data)	R1 = 0.0490, wR2 = 0.1341
Largest diff. peak and hole	0.300 and -0.332 e.$Å^{-3}$

Supplementary Table 5. Bond lengths [Å] and angles [°] for 3ab.

S(1)-N(1)	1.6375(19)
S(1)-C(2)	1.773(2)
S(1)-C(9)	1.867(3)
F(1)-C(9)	1.298(3)
F(2)-C(9)	1.299(3)
F(3)-C(9)	1.302(3)
N(1)-C(1)	1.350(3)
O(1)-C(1)	1.242(3)
O(2)-C(3)	1.349(3)
O(2)-HO2	0.847(18)
C(1)-C(6)	1.497(3)
C(2)-C(8)	1.382(3)
C(2)-C(3)	1.393(3)
C(3)-C(4)	1.388(3)
C(4)-C(5)	1.375(4)
C(4)-H(4)	0.9300
C(5)-C(7)	1.380(4)
C(5)-H(5)	0.9300
C(6)-H(6A)	0.9600
C(6)-H(6B)	0.9600
C(6)-H(6C)	0.9600
C(7)-C(8)	1.388(3)
C(7)-H(7)	0.9300
C(8)-H(8)	0.9300
N(1)-S(1)-C(2)	102.22(10)
N(1)-S(1)-C(9)	101.75(11)
C(2)-S(1)-C(9)	97.44(11)
C(1)-N(1)-S(1)	111.83(16)
C(3)-O(2)-HO2	112(2)
O(1)-C(1)-N(1)	125.1(2)
O(1)-C(1)-C(6)	121.0(2)
N(1)-C(1)-C(6)	113.9(2)
C(8)-C(2)-C(3)	121.7(2)
C(8)-C(2)-S(1)	121.91(17)
C(3)-C(2)-S(1)	116.36(17)

O(2)-C(3)-C(4)	123.3(2)
O(2)-C(3)-C(2)	118.3(2)
C(4)-C(3)-C(2)	118.4(2)
C(5)-C(4)-C(3)	119.9(2)
C(5)-C(4)-H(4)	120.1
C(3)-C(4)-H(4)	120.1
C(4)-C(5)-C(7)	121.6(2)
C(4)-C(5)-H(5)	119.2
C(7)-C(5)-H(5)	119.2
C(1)-C(6)-H(6A)	109.5
C(1)-C(6)-H(6B)	109.5
H(6A)-C(6)-H(6B)	109.5
C(1)-C(6)-H(6C)	109.5
H(6A)-C(6)-H(6C)	109.5
H(6B)-C(6)-H(6C)	109.5
C(5)-C(7)-C(8)	119.3(2)
C(5)-C(7)-H(7)	120.4
C(8)-C(7)-H(7)	120.4
C(2)-C(8)-C(7)	119.1(2)
C(2)-C(8)-H(8)	120.4
C(7)-C(8)-H(8)	120.4
F(1)-C(9)-F(2)	108.4(2)
F(1)-C(9)-F(3)	109.3(2)
F(2)-C(9)-F(3)	107.7(2)
F(1)-C(9)-S(1)	112.00(19)
F(2)-C(9)-S(1)	110.48(18)
F(3)-C(9)-S(1)	108.85(19)

Symmetry transformations used to generate equivalent atoms:

Supplementary Table 6: Condition Screening for C-S bond coupling reaction.

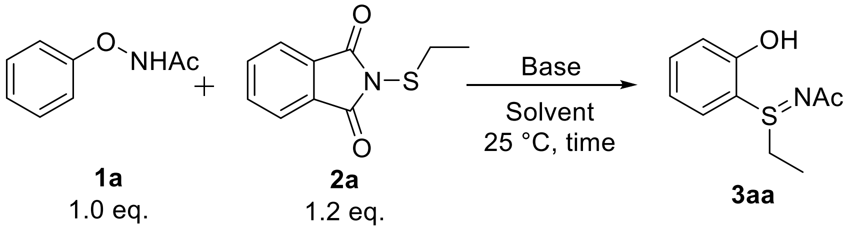

Entry	Solvent	time	Additive(eq.)	Yield(%)[b]
1[c]	MeOH	15h	CsOAc(0.3)	89[a]
2[c]	MeOH	15h	CsOAc(0.3)	92
3	MeOH	15h	CsOAc(0.3)	92
4	MeOH	1h	CsOAc(0.3)	73
5	MeOH	2h	CsOAc(0.3)	87
6	MeOH	3h	CsOAc(0.3)	91
7	MeOH	10h	CsOAc(0.3)	92
8	*i*-PrOH	3h	CsOAc(0.3)	76
9	CH_2Cl_2	3h	CsOAc(0.3)	87
10	CH_3CN	3h	CsOAc(0.3)	85
11	*t*-AmOH	3h	CsOAc(0.3)	74
12	THF	3h	CsOAc(0.3)	81
13	DCE	3h	CsOAc(0.3)	75
14	MeOH	3h	CsOAc(0.3)	88[a]
15	MeOH	10h	-	trace
16	MeOH	3h	CsOAc(0.5)	95
17	MeOH	3h	CsOAc(1.0)	94
18	MeOH	3h	CsOAc(1.5)	95
19	MeOH	3h	CsOAc(0.5)	92[a]
20	PBS buffer	10h	CsOAc(0.3)	72
21	DMSO:PBS=1:20	10h	CsOAc(0.5)	81

[a] Yields of isolated products.

[b] Crude yield, as determined by ^{1}H NMR spectroscopy using 1,4-Dimethoxybenzene as an internal standard.

[c] Under N_2.

Supplementary Methods

General

Commercially available chemicals were obtained from Sigma-Aldrich, Alfa Aesar, TCI and Aladdin and used as received unless otherwise stated. Dichloro(η^5-pentamethylcyclopentadienyl)rhodium(III) dimer (99%) was purchased from Sinocompound Technology Co., Ltd.

Reactions were monitored with analytical thin-layer chromatography (TLC) on silica. ^{1}H NMR and ^{13}C NMR data were recorded on Bruker nuclear resonance (300 MHz, 400 MHz and 500MHz) spectrometers unless otherwise specified, respectively. Chemical shifts (δ) are given in ppm relative to TMS. The residual solvent signals were used as references and the chemical shifts converted to the TMS scale ($CDCl_3$: δ_H=7.26 ppm, δ_C=77.16 ppm; CD_2Cl_2: δ_H=5.32 ppm, δ_C=54.00 ppm; DMSO-d_6: δ_H=2.50 ppm, δ_C=39.52 ppm; MeOD-d_4: δ_H=3.31 ppm, δ_C=49.00 ppm; Acetone-d_6: δ_H=2.05 ppm, δ_C=29.84 ppm, 206.26 ppm). HRMS (ESI) analysis was performed by The Analytical Instrumentation Center at Peking University, Shenzhen Graduate School and (HRMS) data were reported with ion mass/charge (m/z) ratios as values in atomic mass units.

Preparation of starting materials

1) Synthesis of 1a-1k, 1m-1o

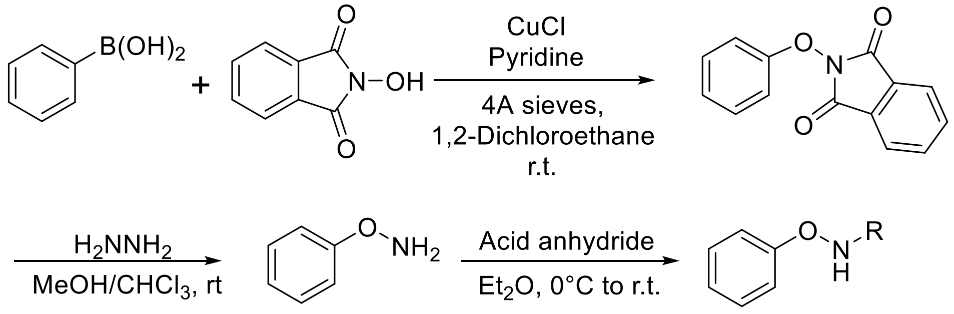

1a-1c

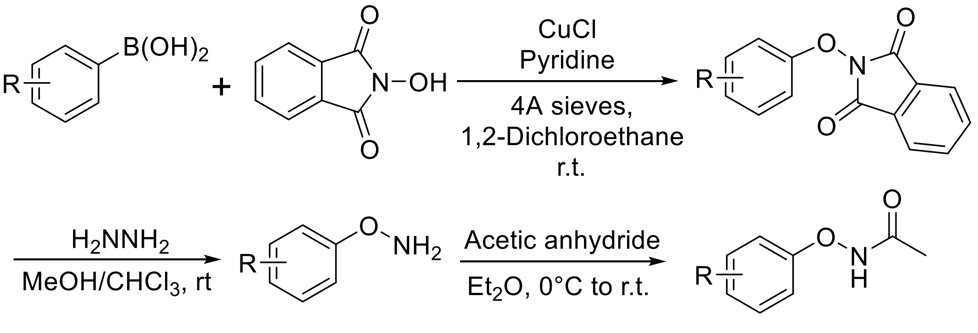

1d-1k, 1m-1o

General procedure:

Following a literature report[1], in a 50mL round-bottom flask,

N-hydroxyphthalimide (1.0 eq.), cooper (I) chloride (1.0 eq.), freshly activated 4 Å molecular sieves (250mg/mmol), and phenylboronic acid (2.0 eq.) were combined in 1,2-dichloroethane(0.2 M). The pyridine (1.1 eq.) was then added to the suspension. The reaction mixture was open to the atmosphere and stirred at room temperature over 24-48h.Upon completion, silica gel was added to the flask and the solvent was removed under vacuum. The desired *N*-aryloxyphthalimides were obtained by flash column chromatography on silica gel.

Hydrazine monohydrate (3.0 eq.) was added to the solution of *N*-aryloxyphthalimide (1.0eq.) in 10% MeOH in $CHCl_3$ (0.1 M). The reaction was allowed to stir at room temperature over 12h. Upon completion, the reaction mixture was filtered off and washed with CH_2Cl_2.The filtrate was concentrated under reduced pressure, and purified by flash silica gel column chromatography to give the corresponding *N*-aryloxyamine.

In a 20 mL round-bottom flask, *N*-aryloxyamine (1.0 eq.) was dissolved in ether (0.2 M). The flask was cooled in an ice bath, to which acid anhydride (2.0 eq.) was slowly added. The ice bath was allowed to warm to room temperature and the mixture was stirred for 3 h at room temperature. The reaction mixture was concentrated under reduced pressure and purified by flash silica gel column chromatography to give the corresponding *N*-acetyl aryloxyamine.

2) Synthesis of 2b

Following a literature report[2]**,** In a glovebox, a 100 ml round bottom flask with 2-chloroisoindoline-1,3-dione (1.81 g, 10.0 mmol), $AgSCF_3$ (2.70g, 13.0 mmol) was added 20 ml dried CH_3CN, the mixture was stirred 3 h at room temperature. Then the solvent was evacuated under rotary evaporator. The residue was added DCM, then filtered through a layer of celite. The filter was evacuated again under reduced pressure.

3) Synthesis of 2a, 2c-2l

Following a literature report[3], sulfuryl chloride (1.0 equiv; ca. 5 M in CH_2Cl_2) was added dropwise via a dropping funnel to a solution of thiol (0.05 mol; ca. 1 M in CH_2Cl_2) and Et_3N (0.1 mL) at 0 ℃. After stirring for 15 min, the mixture was warmed to r.t. for 30 min and then cooled to 0 ℃. The resulting solution was transferred dropwise via cannula to a solution of phthalimide (1.0 equiv; ca. 1 M in CH_2Cl_2) and Et_3N (1.3 equiv) at 0 ℃ and the mixture was then warmed to r.t. over 1 h. The solution was diluted with H_2O, extracted with CH_2Cl_2 (3×) before being dried over Na_2SO_4, and then concentrated to give crude product that was purified using recrystallization. For samples with appreciable amounts of phthalimide present, the crude was dissolved with CH_2Cl_2, diluted with 1 M NaOH, extracted with CH_2Cl_2 (3×) before being dried over Na_2SO_4, then concentrated before being purified by recrystallization.

4) Synthesis of 1l, 1p

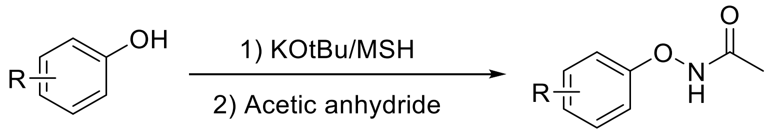

Following a literature report[4], NaH (1.5 eq.) was added to a solution of 7-hydroxycoumarin (1.0 eq.) in anhydrous DMF (0.3 M). The mixture was allowed to stir at room temperature over 1h, followed by the addition of freshly prepared *O*-(mesitylsulfonyl)hydroxylamine(1.2 eq.). The reaction mixture was further stirred at room temperature for 12h. Upon completion, the mixture was cooled to 0 ℃, followed by quenching with water and extracted with ethyl acetate. The combined organic layer was dried over Na_2SO_4, concentrated under reduced pressure to give the crude product *N*-aryloxyamine.

In a 20 mL round-bottom flask, the crude product *N*-aryloxyamine (1.0 eq.) was dissolved in dichloromethane (0.2 M). The flask was cooled in an ice bath, to which acetic anhydride (2.0 eq.) was slowly added. The ice bath was allowed to warm to room temperature and the mixture was stirred for 3 h at room temperature. The reaction mixture was concentrated under reduced pressure and purified by flash silica gel column chromatography to give the corresponding *N*-acetyl aryloxyamine.

5) Synthesis of 1q

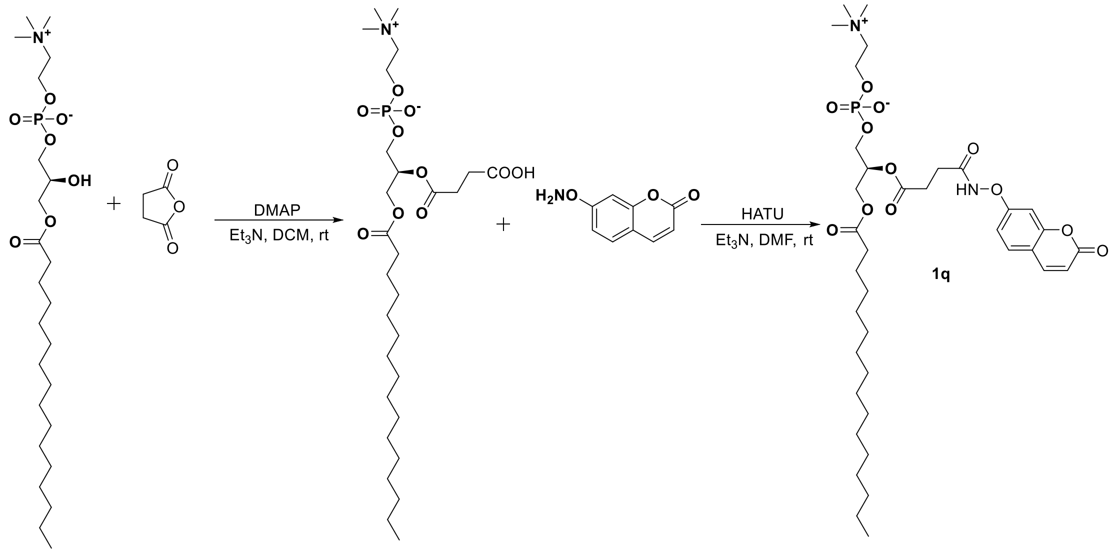

1-palmitoyl-2-hydroxy-*sn*-glycero-3-phosphocholine (50mg), Succinic anhydride (20mg) and 4-(dimethylamino)pyridine (5mg) were dissolved in 2 mL methylene chloride. To this, 15μL Et_3N was added. The solution was stirred overnight at rt, After the reaction was finished, removed the solvent by rotary evaporation. Then 7-(aminooxy)-2H-chromen-2-one (35mg), *O*-(7-Azabenzotriazol-1-yl)-N, N, N', N'-tetramethyluronium hexafluorophosphate (50mg), Et_3N (15μL) and DMF (1mL) were added. The solution was stirred overnight at room temperature. After the reaction, 5 mL water was added and extracted with DCM. After drying with $MgSO_4$, the solvent was removed by rotary evaporation and the product isolated by column

chromatography as a white solid.
Yield of two steps: 45%.

Characterization Data

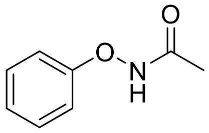

***N*-phenoxyacetamide, 1a, white solid, yield: 60%**

^{1}H NMR (400 MHz, DMSO-d_6): δ 11.67 (s, 1H), 7.32 (t, *J* = 7.9 Hz, 2H), 7.01 (t, *J* = 7.6 Hz, 3H), 1.92 (s, 3H); ^{13}C NMR (101 MHz, DMSO-d_6): δ 167.22, 159.55, 129.45, 122.25, 112.86, 19.43.

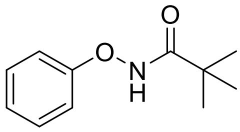

***N*-phenoxypivalamide, 1b, white solid, yield: 48%**

^{1}H NMR (300 MHz, DMSO-d_6): δ 11.62 (s, 1H), 7.32 (t, *J* = 7.8 Hz, 2H), 7.00 (dd, *J* = 14.3, 7.6 Hz, 3H), 1.20 (s, 9H); ^{13}C NMR (75 MHz, DMSO-d_6): δ 175.65, 160.23, 129.88, 122.54, 113.17, 38.04, 27.48.

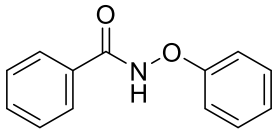

***N*-phenoxybenzamide, 1c, white solid, yield: 48%**

^{1}H NMR (300 MHz, DMSO-d_6): 12.49 (s, 1H), 7.94 – 7.81 (m, 2H), 7.62 (t, *J* = 7.2 Hz, 1H), 7.53 (t, *J* = 7.3 Hz, 2H), 7.41 – 7.28 (m, 2H), 7.06 (dd, *J* = 16.8, 7.9 Hz, 3H); ^{13}C NMR (75 MHz, DMSO-d_6): δ 160.07, 132.57, 131.94, 130.00, 129.13, 127.76, 122.83, 113.45.

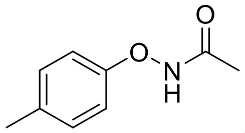

***N*-(p-tolyloxy)acetamide, 1d, white solid, yield: 45%**

^{1}H NMR (400 MHz, DMSO-d_6): δ 11.61 (s, 1H), 7.10 (d, *J* = 8.0 Hz, 2H), 6.90 (d, *J* = 7.9 Hz, 2H), 2.24 (s, 3H), 1.90 (s, 3H); ^{13}C NMR (101 MHz, DMSO-d_6): δ 167.58, 158.00, 131.51, 130.14, 113.28, 20.52, 19.84.

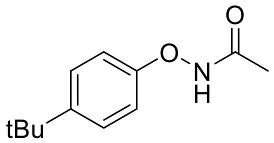

***N*-(4-(tert-butyl)phenoxy)acetamide, 1e, white solid, yield: 45%**

^{1}H NMR (400 MHz, Acetone-d_6): δ 10.89 (s, 1H), 7.33 (d, *J* = 7.9 Hz, 2H), 6.97 (d, *J*

= 7.7 Hz, 2H), 1.95 (s, 3H), 1.29 (s, 9H); ^{13}C NMR (101 MHz, Acetone-d_6): δ 167.83, 157.81, 144.89, 125.98, 112.64, 33.79, 30.95, 18.80.

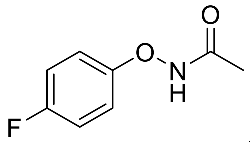

***N*-(4-fluorophenoxy)acetamide, 1f, white solid, yield: 50%**

^{1}H NMR (400 MHz, DMSO-d_6): δ 11.72 (s, 1H), 7.14 (t, *J* = 8.7 Hz, 2H), 7.03 (dd, *J* = 8.4, 4.2 Hz, 2H), 1.90 (s, 3H); ^{13}C NMR (101 MHz, DMSO-d_6): δ 167.32, 157.43（*J* = 237.4 Hz）, 155.77, 115.83(*J* = 24.2 Hz), 114.48(*J*=8.1 Hz), 19.41.

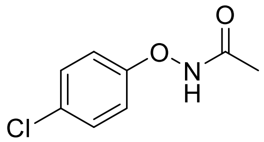

***N*-(4-chlorophenoxy)acetamide, 1g, white solid, yield: 45%**

^{1}H NMR (400 MHz, DMSO-d_6): δ 11.74 (s, 1H), 7.35 (d, *J* = 8.8 Hz, 2H), 7.03 (d, *J* = 8.6 Hz, 2H), 1.91 (s, 3H); ^{13}C NMR (101 MHz, DMSO-d_6): δ 167.88, 158.89, 129.68, 126.34, 115.22, 19.87.

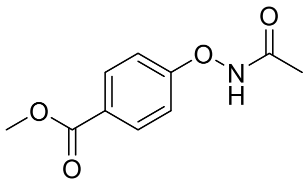

Methyl 4-(acetamidooxy)benzoate, 1h, white solid, yield: 47%

^{1}H NMR (400 MHz, DMSO) δ 11.86 (s, 1H), 7.94 (d, *J* = 8.7 Hz, 1H), 7.13 (d, *J* = 8.5 Hz, 1H), 3.83 (s, 1H), 1.94 (s, 1H); ^{13}C NMR (101 MHz, DMSO) δ 167.97, 166.12, 163.72, 131.65, 124.12, 113.40, 52.37, 19.80.

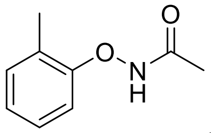

***N*-(o-tolyloxy)acetamide, 1i, white solid, yield: 43%**

^{1}H NMR (400 MHz, DMSO-d_6): δ 11.62 (s, 1H), 7.15 (t, *J* = 7.7 Hz, 2H), 6.99 (d, *J* = 8.0 Hz, 1H), 6.92 (t, *J* = 7.3 Hz, 1H), 2.21 (s, 3H), 1.92 (s, 3H) ; ^{13}C NMR (101 MHz, DMSO-d_6): δ 167.09, 157.46, 130.69, 126.87, 123.50, 121.99, 111.54, 19.44, 15.48.

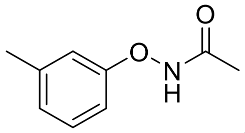

***N*-(m-tolyloxy)acetamide, 1j, white solid, yield: 50%**

^{1}H NMR (400 MHz, Acetone-d$_6$): δ 10.75 (s, 1H), 7.16 (t, *J* = 7.0 Hz, 1H), 6.85 (d, *J* = 14.0 Hz, 3H), 2.29 (s, 3H), 1.97 (s, 3H); ^{13}C NMR (101 MHz, Acetone-d$_6$): δ 167.75, 160.08, 139.21, 129.01, 122.99, 113.51, 109.98, 20.54, 18.75.

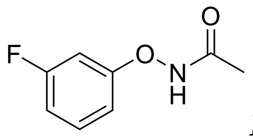

***N*-(3-fluorophenoxy)acetamide, 1k, white solid, yield: 35%**

^{1}H NMR (400 MHz, DMSO-d$_6$): δ 11.76 (s, 1H), 7.34 (dd, *J* = 15.4, 8.0 Hz, 1H), 6.98 – 6.80 (m, 3H), 1.92 (s, 3H); ^{13}C NMR (101MHz, DMSO-d$_6$): δ 167.92, 163.27 (*J* = 244.5 Hz), 161.52 (*J*=10.6 Hz), 131.28 (*J* = 10.2 Hz), 109.56, 109.34 (*J* = 21.4 Hz), 101.12 (*J* = 26.7 Hz), 19.85.

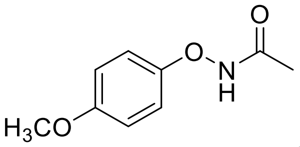

***N*-(4-methoxyphenoxy)acetamide, 1l, brown solid, yield: 45%**

^{1}H NMR (400 MHz, DMSO-d$_6$) δ 11.61 (s, 1H), 6.94 (d, *J* = 8.6 Hz, 1H), 6.87 (d, *J* = 8.7 Hz, 1H), 3.71 (s, 1H), 1.88 (s, 1H); ^{13}C NMR (101 MHz, DMSO-d$_6$) δ 167.53, 155.05, 153.95, 114.87, 114.70, 55.89, 19.89; HRMS (ESI) calculated for $C_9H_{12}NO_3$ $[M+H]^+$: 182.0817; Found: 182.0808.

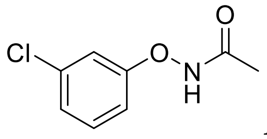

***N*-(3-chlorophenoxy)acetamide, 1m, white solid, yield: 40%**

^{1}H NMR (400 MHz, DMSO-d$_6$): δ 11.77 (s, 1H), 7.33 (t, *J* = 8.3 Hz, 1H), 7.08 (m, 2H), 6.99 (d, *J* = 8.1 Hz, 1H), 1.92 (s, 3H); ^{13}C NMR (101 MHz, DMSO-d$_6$): δ 168.01, 160.94, 134.19, 131.43, 122.72, 113.44, 112.42, 19.89.

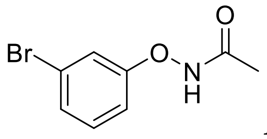

***N*-(3-bromophenoxy)acetamide, 1n, white solid, yield: 30%**

^{1}H NMR (400 MHz, DMSO-d$_6$): δ 11.76 (s, 1H), 7.27 (t, *J* = 8.2 Hz, 1H), 7.21 (d, *J* = 5.4 Hz, 2H), 7.03 (d, *J* = 8.1 Hz, 1H), 1.92 (s, 3H); ^{13}C NMR (101 MHz, DMSO-d$_6$): δ 168.02, 160.93, 131.76, 125.65, 122.41, 116.21, 112.83, 19.90.

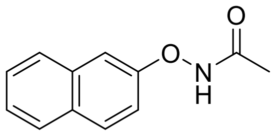

***N*-(naphthalen-2-yloxy)acetamide, 1o, white solid, yield: 52%**

^{1}H NMR (400 MHz, DMSO-d$_6$) : δ 11.82 (s, 1H), 7.87 (dd, *J* = 17.2, 9.1 Hz, 3H), 7.48 (t, *J* = 7.4 Hz, 1H), 7.40 (dd, *J* = 13.9, 5.8 Hz, 2H), 7.27 (d, *J* = 8.9 Hz, 1H), 1.98 (s, 3H); ^{13}C NMR (101 MHz, DMSO-d$_6$) : δ 167.31, 157.32, 133.75, 129.51, 129.38, 127.62, 126.90, 126.70, 124.22, 115.40, 106.84, 19.56.

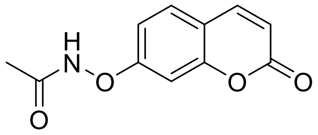

***N*-((2-oxo-2H-chromen-7-yl)oxy)acetamide, 1p, white solid, yield: 30%**

^{1}H NMR (500 MHz, MeOD) : δ 7.90 (d, *J* = 9.5 Hz, 1H), 7.60 (d, *J* = 8.5 Hz, 1H), 7.11 – 6.97 (m, 2H), 6.31 (d, *J* = 9.5 Hz, 1H), 2.07 (s, 3H); ^{13}C NMR (126 MHz, MeOD) : δ 164.19, 162.86, 156.78, 145.36, 130.72, 115.77, 114.63, 111.80, 101.77, 19.30; HRMS (ESI) calculated for $C_{11}H_{10}NO_4$ [M+H]$^+$: 220.0610; Found: 220.0612.

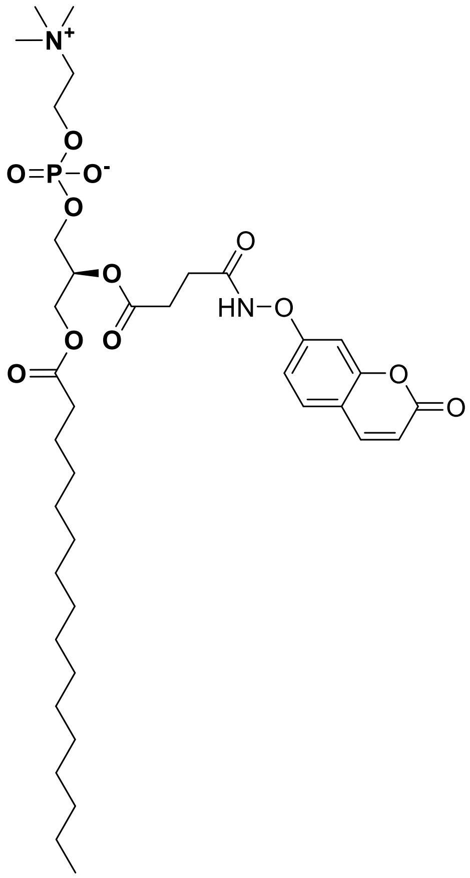

1q, white solid, yield: 45%.

(*S*)-2-((4-oxo-4-(((2-oxo-2H-chromen-7-yl)oxy)amino)butanoyl)oxy)-3-(palmitoyloxy)propyl (2-(trimethylammonio)ethyl) phosphate

^{1}H NMR (400 MHz, MeOD): δ 7.92 (d, *J* = 9.5 Hz, 1H), 7.62 (d, *J* = 8.5 Hz, 1H), 7.19 – 7.03 (m, 2H), 6.32 (d, *J* = 9.5 Hz, 1H), 5.32 (s, 1H), 4.62 (s, 2H), 4.41 (dd, *J* = 12.0, 3.6 Hz, 1H), 4.35 – 4.16 (m, 3H), 4.07 (t, *J* = 5.1 Hz, 2H), 3.73 – 3.58 (m, 2H), 3.23 (d, *J* = 8.9 Hz, 8H), 2.79 (d, *J* = 5.8 Hz, 2H), 2.62 (t, *J* = 6.2 Hz, 2H), 2.39 – 2.21 (m, 2H), 1.62 – 1.49 (m, 2H), 1.30 (dd, *J* = 18.5, 13.7 Hz, 23H), 0.91 (t, *J* = 6.8

Hz, 3H); ^{13}C NMR (101 MHz, MeOD): δ 173.70, 172.10, 162.99, 161.49, 155.43, 144.04, 129.32, 114.26, 113.16, 110.53, 100.44, 71.06, 70.98, 66.04, 63.65, 63.59, 62.05, 59.14, 59.09, 53.33, 53.29, 53.25, 33.35, 31.67, 29.38, 29.36, 29.33, 29.22, 29.07, 29.00, 28.77, 26.80, 24.49, 22.33, 13.04; ^{31}P NMR (162 MHz, MeOD): δ -0.55; HRMS (ESI) calculated for $C_{37}H_{60}N_2O_{12}P$ $[M+H]^+$:755.3884; Found: 755.3879.

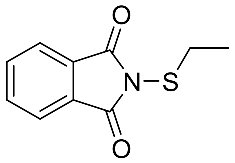

2-(ethylthio)isoindoline-1,3-dione, 2a, white solid, yield: 80%

^1H NMR (500 MHz, $CDCl_3$): δ 7.94 (dd, *J* = 5.5, 3.0 Hz, 2H), 7.79 (dd, *J* = 5.5, 3 Hz, 2H), 2.92 (q, *J* = 7 Hz, 2H), 1.29 (t, *J* = 7 Hz, 3H); ^{13}C NMR (126 MHz, $CDCl_3$): δ 168.8, 134.8, 132.3, 124.1, 32.9, 13.4; HRMS (ESI) calculated for $C_{10}H_{10}NO_2S$ $[M+H]^+$: 208.0432; Found: 208.0430.

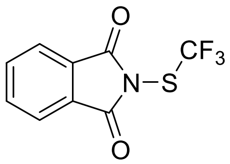

2-((trifluoromethyl)thio)isoindoline-1,3-dione, 2b, white solid, yield: 70%

^1H NMR (400 MHz, $CDCl_3$): δ 8.03 (dd, *J* = 5.5, 3.1 Hz, 2H), 7.88 (dd, *J* = 5.6, 3.1 Hz, 2H); ^{13}C NMR (101 MHz, $CDCl_3$) δ 165.78, 135.43, 131.46, 127.90 (q, *J* =314.8 Hz), 124.70; ^{19}F NMR (376 MHz, $CDCl_3$): δ -48.90 (s, 3F); HRMS (ESI) calculated for $C_9H_5F_3NO_2S$ $[M+H]^+$: 247.9993; Found: 247.9989.

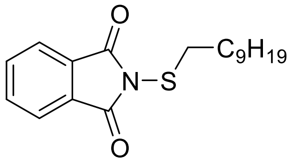

2-(decylthio)isoindoline-1,3-dione, 2c, white solid, yield: 76%

^1H NMR (500 MHz, $CDCl_3$): δ 7.92 (dd, *J* = 5.5, 3.0 Hz, 2H), 7.79 (dd, *J* = 5.5, 3 Hz, 2H), 2.89 (t, *J* = 7.5 Hz, 2H), 1.62-1.52 (m, 2H), 1.43-1.39 (m, 2H), 1.30-1.20 (m, 12H), 0.87 (t, *J* = 7 Hz, 3H); ^{13}C NMR (126 MHz, $CDCl_3$): δ 168.6, 134.7, 132.3, 124.0, 38.8, 32.0, 29.63, 29.57, 29.4, 9.2, 28.6, 28.3, 22.8, 14.2; HRMS (ESI) calculated for $C_{18}H_{26}NO_2S$ $[M+H]^+$: 320.1684; Found: 320.1687.

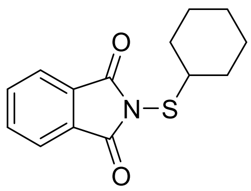

2-(cyclohexylthio)isoindoline-1,3-dione, 2d, white solid, yield: 75%

^1H NMR (500 MHz, $CDCl_3$): δ 7.93 (dd, *J* = 5.5, 3.1 Hz, 2H), 7.78 (dd, *J* = 5.5, 3.0 Hz, 2H), 3.14 (tt, *J* = 10.9, 3.6 Hz, 1H), 1.91 – 1.88 (m, 2H), 1.79 – 1.70 (m, 2H), 1.65 – 1.57 (m, 1H), 1.39 – 1.29 (m, 2H), 1.28 – 1.13 (m, 3H); ^{13}C NMR (126 MHz, $CDCl_3$): δ 168.8, 134.5, 132.0, 123.8, 49.4, 31.1, 25.5, 25.4; HRMS (ESI) calculated for $C_{14}H_{16}NO_2S$ $[M+H]^+$: 262.0902; Found: 262.0899.

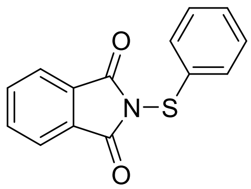

2-(phenylthio)isoindoline-1,3-dione, 2e, yellow solid, yield: 82%

^1H NMR (500 MHz, $CDCl_3$): δ 7.93 (dd, *J* = 5.5, 3.1 Hz, 1H), 7.78 (dd, *J* = 5.5, 3.0 Hz, 1H), 7.65 – 7.56 (m, 2H), 7.37 – 7.28 (m, 3H); ^{13}C NMR (126 MHz, $CDCl_3$): δ 167.7, 135.1, 134.7, 132.1, 130.9, 124.0; HRMS (ESI) calculated for $C_{14}H_{10}NO_2S$ $[M+H]^+$: 256.0432; Found: 256.0430.

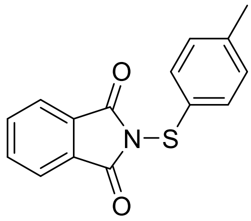

2-(phenylthio)isoindoline-1,3-dione, 2f, yellow solid, yield: 84%

^1H NMR (400 MHz, $CDCl_3$):δ 7.91 (dd, *J* = 5.5, 3.1 Hz, 1H), 7.76 (dd, *J* = 5.5, 3.1 Hz, 1H), 7.62 – 7.58 (d, *J* = 7.9 Hz, 2H), 7.14 (d, *J* = 7.9 Hz, 2H), 2.32 (s, 3H); ^{13}C NMR (101 MHz, $CDCl_3$): δ 168.0, 140.5, 134.7, 132.8, 132.2, 131.6, 130.2, 124.1, 21.4; HRMS (ESI) calculated for $C_{15}H_{12}NO_2S$ $[M+H]^+$: 270.0589; Found: 270.0591.

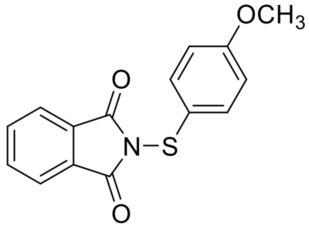

2-((4-methoxyphenyl)thio)isoindoline-1,3-dione, 2g, white solid, yield: 71%

^1H NMR (500 MHz, $CDCl_3$): δ = 7.87 (dd, *J*=5.5, 3.0 Hz, 2H), 7.76 (d, *J* = 9.0 Hz, 2H), 7.73 (dd, *J* = 5.5, 3 Hz, 2H), 6.84 (d, *J* = 9.0 Hz, 2H), 3.78 (s, 3H); ^{13}C NMR

(126 MHz, $CDCl_3$): δ 168.0, 161.6, 136.8, 134.7, 132.2, 125.7, 124.0, 114.9, 55.6; HRMS (ESI) calculated for $C_{15}H_{12}NO_3S$ $[M+H]^+$: 286.0538; Found: 286.0534.

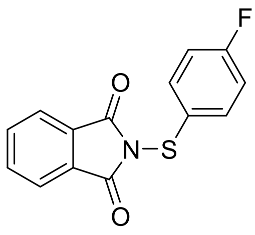

2-((4-fluorophenyl)thio)isoindoline-1,3-dione, 2h, yellow solid, yield: 80%

^{1}H NMR (400 MHz, $CDCl_3$):δ 7.91 (dd, *J* = 5.5, 3.1 Hz, 2H), 7.77 (dd, *J* = 5.5, 3.0 Hz, 2H), 7.75 – 7.72 (m, 2H), 7.05 – 6.99 (m, 2H); ^{13}C NMR (101 MHz, $CDCl_3$):δ 167.6, 165.0, 162.5, 135.6(*J* = 20 Hz), 134.7, 131.9, 124.0, 116.5(*J* =44 Hz); HRMS (ESI) calculated for $C_{14}H_9FNO_2S$ $[M+H]^+$: 274.0338; Found: 274.0336.

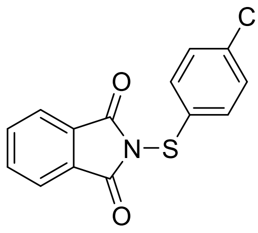

2-((4-chlorophenyl)thio)isoindoline-1,3-dione, 2i, yellow solid, yield: 82%

^{1}H NMR (300 MHz, $CDCl_3$):δ 7.93 (dd, *J* = 5.5, 3.1 Hz, 2H), 7.79 (dd, *J* = 5.5, 3.1 Hz, 2H), 7.57 (d, *J* = 8.8 Hz, 2H), 7.30 (d, *J* = 8.8 Hz, 2H); ^{13}C NMR (75 MHz, $CDCl_3$):δ 167.5, 135.8, 134.8, 133.3, 132.8, 131.8, 129.5, 124.1; HRMS (ESI) calculated for $C_{14}H_9ClNO_2S$ $[M+H]^+$: 290.0043; Found: 290.0039.

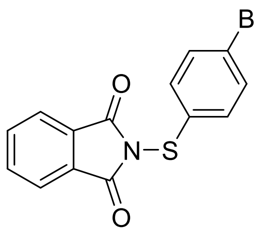

2-((4-bromophenyl)thio)isoindoline-1,3-dione, 2j, yellow solid, yield: 81%

^{1}H NMR (400 MHz, $CDCl_3$): δ 7.93 (dd, *J* = 5.5, 3.1 Hz, 2H), 7.79 (dd, *J* = 5.5, 3.1 Hz, 2H), 7.51 – 7.42 (m, 4H); ^{13}C NMR (101 MHz, $CDCl_3$): δ 167.5, 134.8, 134.0, 132.7, 132.5, 131.9, 124.1, 123.9; HRMS (ESI) calculated for $C_{14}H_9BrNO_2S$ $[M+H]^+$: 333.9537; Found: 333.9533.

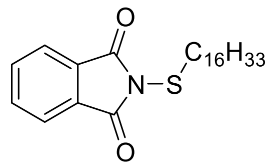

2-(hexadecylthio)isoindoline-1,3-dione, 2k, white solid, yield: 76%

^{1}H NMR (400 MHz, $CDCl_3$) δ 8.01 – 7.89 (m, 2H), 7.87 – 7.75 (m, 2H), 2.97 – 2.84 (m, 2H), 1.64 – 1.56 (m, 3H), 1.41 (dd, *J* = 14.0, 7.2 Hz, 2H), 1.35 – 1.21 (m, 27H), 0.89 (t, *J* = 6.8 Hz, 3H); ^{13}C NMR (101 MHz, $CDCl_3$) δ 168.56, 134.55, 132.09, 123.86, 38.66, 31.93, 29.68, 29.66, 29.62, 29.55, 29.44, 29.36, 29.12, 28.46, 28.14, 22.70, 14.13; HRMS (ESI) calculated for $C_{24}H_{38}NO_2S$ $[M+H]^+$: 404.2623; Found: 404.2621.

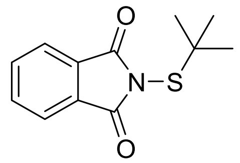

2-(tert-butylthio)isoindoline-1,3-dione, 2l, white solid, yield:30%

^{1}H NMR (500 MHz, $CDCl_3$) δ 7.93 (dd, *J* = 5.5, 3.1 Hz, 2H), 7.78 (dd, *J* = 5.5, 3.1 Hz, 2H), 1.35 (s, 9H); ^{13}C NMR (126 MHz, $CDCl_3$) δ 169.05, 134.58, 131.96, 123.91, 51.03, 29.36; HRMS (ESI) calculated for $C_{12}H_{13}NNaO_2S$ $[M+Na]^+$: 258.0565; Found: 258.0564.

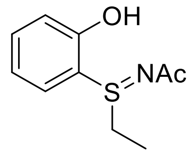

(*E*)-*N*-(ethyl(2-hydroxyphenyl)-λ^4-sulfanylidene)acetamide, 3aa, white solid, yield: 92%

^{1}H NMR (500 MHz, $CDCl_3$): δ 7.45 – 7.41 (m, 1H), 7.24 (dd, *J* = 7.9, 1.6 Hz, 1H), 6.99 (dd, *J* = 8.4, 0.9 Hz, 1H), 6.94 – 6.91 (m, 1H), 3.35 – 3.16 (m, 2H), 2.14 (s, 3H), 1.25 (t, *J* = 7.4 Hz, 3H); ^{13}C NMR (126 MHz, $CDCl_3$): δ 181.27, 159.56, 133.80, 128.06, 119.73, 119.05, 113.18, 42.79, 24.39, 7.78; HRMS (ESI) calculated for $C_{10}H_{12}NO_2S$ $[M–H]^-$: 210.0594; Found: 210.0592.

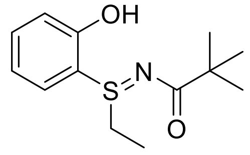

(*E*)-*N*-(ethyl(2-hydroxyphenyl)-λ^4-sulfanylidene)pivalamide, 3ba, white solid, yield: 80%

^{1}H NMR (500 MHz, CD_2Cl_2) δ 7.45 (t, *J* = 7.8 Hz, 1H), 7.29 (dd, *J* = 7.8, 1.3 Hz, 1H), 6.98 (d, *J* = 8.4 Hz, 1H), 6.95 (t, *J* = 7.8 Hz, 1H), 3.33 (tt, *J* = 14.7, 7.4 Hz, 1H),

3.15 (dq, J = 14.8, 7.4 Hz, 1H), 1.25 – 1.19 (m, 12H); ^{13}C NMR (126 MHz, CD_2Cl_2) δ 188.16, 166.51, 133.86, 129.16, 123.20, 119.81, 119.38, 43.76, 39.93, 28.02, 6.93; HRMS (ESI) calculated for $C_{13}H_{20}NO_2S$ $[M+H]^+$: 254.1215; Found: 254.1208.

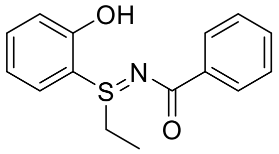

(*E*)-*N*-(ethyl(2-hydroxyphenyl)-λ^4-sulfanylidene)benzamide, 3ca, white solid, yield: 83%

^{1}H NMR (400 MHz, $CDCl_3$) δ 8.16 – 8.14 (m, 2H), 7.51 – 7.49 (m, 1H), 7.46 (t, J = 7.4 Hz, 2H), 7.36 – 7.29 (m, 2H), 6.97 – 6.91 (m, 2H), 3.33 (tdd, J = 12.9, 7.4, 5.6 Hz, 2H), 1.30 (t, J = 7.4 Hz, 3H); ^{13}C NMR (101 MHz, $CDCl_3$) δ175.74, 160.05, 135.44, 134.05, 131.41, 128.75, 128.46, 128.09, 123.50, 119.78, 119.46, 112.62, 43.38, 7.75; HRMS (ESI) calculated for $C_{15}H_{16}NO_2S$ $[M+H]^+$: 274.0902; Found: 274.0894.

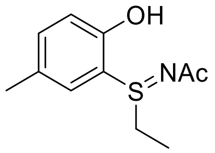

(*E*)-*N*-(ethyl(2-hydroxy-5-methylphenyl)-λ^4-sulfanylidene)acetamide, 3da, white solid, yield: 90%

^{1}H NMR (500 MHz, CD_2Cl_2) δ 7.26 (dd, J = 8.3, 1.8 Hz, 1H), 7.09 (d, J = 1.4 Hz, 1H), 6.88 (d, J = 8.4 Hz, 1H), 3.30 – 3.18 (m, 2H), 2.30 (s, 3H), 2.09 (s, 3H), 1.25 (t, J = 7.4 Hz, 3H); ^{13}C NMR (126 MHz, CD_2Cl_2) δ 180.08, 158.62, 134.81, 129.34, 128.79, 119.56, 111.34, 43.21, 23.79, 19.86, 7.40; HRMS (ESI) calculated for $C_{11}H_{16}NO_2S$ $[M+H]^+$:226.0902; Found: 226.0896.

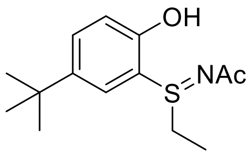

(*E*)-*N*-((5-(tert-butyl)-2-hydroxyphenyl)(ethyl)-λ^4-sulfanylidene)acetamide, 3ea, white solid, yield: 90%

^{1}H NMR (500 MHz, $CDCl_3$) δ 7.35 (dd, J = 8.7, 2.4 Hz, 1H), 7.21 (d, J = 2.4 Hz, 1H), 6.80 (d, J = 8.7 Hz, 1H), 3.24 – 3.16 (m, 2H), 2.12 (s, 3H), 1.26 – 1.22 (m, 12H); ^{13}C NMR (126 MHz, $CDCl_3$) δ 180.83, 155.77, 142.80, 131.24, 124.92, 119.12, 111.40, 43.13, 34.15, 31.21, 24.24, 14.10, 7.87; HRMS (ESI) calculated for $C_{14}H_{22}NO_2S$ $[M+H]^+$: 268.1371; Found: 268.1367.

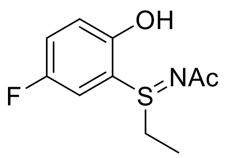

(E)-N-(ethyl(5-fluoro-2-hydroxyphenyl)-λ^4-sulfanylidene)acetamide, 3fa, white solid, yield: 85%

^{1}H NMR (400 MHz, CD_2Cl_2) δ 7.15 – 7.00 (m, 2H), 6.82 (dd, J = 8.6, 4.4 Hz, 1H), 3.31 – 3.07 (m, 2H), 2.10 (s, 3H), 1.25 (t, J = 7.4 Hz, 3H); ^{13}C NMR (126 MHz, CD_2Cl_2) δ 180.92, 155.47(J = 241.5 Hz), 130.72(J = 226.0 Hz), 128.53(J = 284.8 Hz), 120.73(J = 22.7 Hz), 120.27(J = 7.6 Hz) 114.14(J = 23.9 Hz), 42.82, 23.88, 7.41; HRMS (ESI) calculated for $C_{10}H_{13}FNO_2S$ $[M+H]^+$: 230.0651; Found: 230.0648.

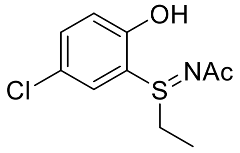

(E)-N-((5-chloro-2-hydroxyphenyl)(ethyl)-λ^4-sulfanylidene)acetamide, 3ga, white solid, yield: 86%

^{1}H NMR (500 MHz, MeOD) δ 7.47 (d, J = 2.5 Hz, 1H), 7.42 (dd, J = 8.7, 2.5 Hz, 1H), 6.96 (d, J = 8.7 Hz, 1H), 3.34 – 3.28 (m, 1H), 3.24 – 3.16 (m, 1H), 2.14 (s, 3H), 1.25 (t, J = 7.3 Hz, 3H); ^{13}C NMR (126 MHz, MeOD) δ 182.65, 155.52, 132.89, 126.03, 124.43, 118.54, 117.80, 40.34, 22.84, 6.44; HRMS (ESI) calculated for $C_{10}H_{13}ClNO_2S$ $[M+H]^+$: 246.0356; Found: 246.0351.

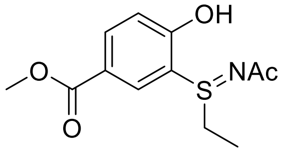

Methyl (E)-3-(N-acetyl-S-ethylsulfinimidoyl)-λ^4-hydroxybenzoate, 3ha, white solid, yield: 89%

^{1}H NMR (500 MHz, $CDCl_3$) δ 8.83 (s, 1H), 7.98 (dd, J = 7.1, 2.0 Hz, 1H), 7.95 – 7.87 (m, 1H), 6.82 (d, J = 8.7 Hz, 1H), 3.86 (s, 3H), 3.23 – 3.20 (m, 2H), 2.16 (s, 3H), 1.23 (t, J = 7.4, 3H); ^{13}C NMR (126 MHz, $CDCl_3$) δ 181.18, 165.42, 163.68, 135.01, 130.23, 121.86, 118.88, 113.24, 52.09, 43.01, 24.20, 7.53; HRMS (ESI) calculated for $C_{12}H_{16}NO_4S$ $[M+H]^+$: 270.0800; Found: 270.0794.

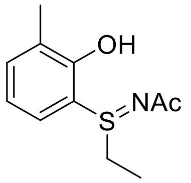

(E)-N-(ethyl(2-hydroxy-3-methylphenyl)-λ^4-sulfanylidene)acetamide, 3ia, white solid, yield: 89%

^{1}H NMR (500 MHz, MeOD) δ 7.35 (dd, *J* = 13.7, 7.7 Hz, 2H), 6.97 (t, *J* = 7.7 Hz, 1H), 3.33 – 3.27 (m, 1H), 3.25 – 3.18 (m, 1H), 2.27 (s, 3H), 2.11 (s, 3H), 1.24 (t, *J* = 7.4 Hz, 3H); ^{13}C NMR (126 MHz, MeOD) δ 181.90, 155.86, 134.69, 127.72, 125.28, 120.07, 114.96, 41.63, 22.76, 14.59, 6.66; HRMS (ESI) calculated for $C_{11}H_{16}NO_2S$ $[M+H]^+$: 226.0902; Found: 226.0899.

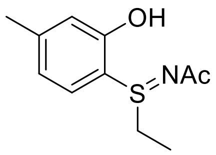

(*E*)-*N*-(ethyl(2-hydroxy-4-methylphenyl)-λ^4-sulfanylidene)acetamide, 3ja, white solid, 3ja:3ja’=1.1:1, yield: 89%

^{1}H NMR (500 MHz, $CDCl_3$) δ 7.12 (d, *J* = 8.2 Hz, 1H), 6.72 (s, 1H), 6.71 (d, *J* = 7.6 Hz, 1H), 3.24-3.14 (m, *J* = 12.8, 7.4, 5.5 Hz, 2H), 2.29 (s, 3H), 2.13 (s, 3H), 1.22 (t, *J* = 7.4 Hz, 3H); ^{13}C NMR (126 MHz, $CDCl_3$) δ 180.67, 160.36, 145.23, 128.53, 120.75, 120.16, 108.59, 43.27, 24.18, 21.44, 7.69; HRMS (ESI) calculated for $C_{11}H_{16}NO_2S$ $[M+H]^+$:226.0902; Found: 226.0893.

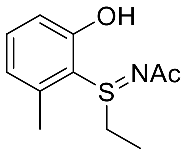

(*E*)-*N*-(ethyl(2-hydroxy-6-methylphenyl)-λ^4-sulfanylidene)acetamide, 3ja’, white solid, 3ja:3ja’=1.1:1, yield: 89%

^{1}H NMR (500 MHz, $CDCl_3$) δ 7.29 (dd, *J* = 15.0, 7.1 Hz, 1H), 6.83 (d, *J* = 8.3 Hz, 1H), 6.74 (d, *J* = 7.5 Hz, 1H), 3.39 – 3.14 (m, 2H), 2.46 (s, 3H), 2.13 (s, 3H), 1.26 (d, *J* = 7.4 Hz, 3H); ^{13}C NMR (126 MHz, $CDCl_3$) δ 180.30, 162.29, 138.85, 134.00, 121.76, 118.09, 42.57, 24.26, 20.03, 7.90; HRMS (ESI) calculated for $C_{11}H_{16}NO_2S$ $[M+H]^+$:226.0902; Found: 226.0898.

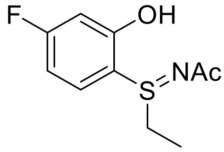

(*E*)-*N*-(ethyl(4-fluoro-2-hydroxyphenyl)-λ^4-sulfanylidene)acetamide, 3ka, white solid, 3ka:3ka’=1.05:1, yield: 84%

^{1}H NMR (400 MHz, CD_2Cl_2) δ 8.99 (s, 1H), 7.27 (dd, *J* = 8.8, 6.1 Hz, 1H), 6.61 (td, *J* = 8.6, 2.5 Hz, 1H), 6.48 (dd, *J* = 10.3, 2.5 Hz, 1H), 3.25 – 3.00 (m, 2H), 2.14 (s, 3H), 1.23 (t, *J* = 7.4 Hz, 3H); ^{13}C NMR (101 MHz, CD_2Cl_2) δ 180.83, 166.24(*J*=353.5 Hz), 161.80(*J* = 13.1 Hz), 129.90(*J* = 12.1 Hz), 128.57(*J* = 225.2 Hz), 107.30(*J* = 23.2 Hz), 106.00(*J* = 25.2 Hz), 42.95, 24.01, 7.43; HRMS (ESI) calculated for $C_{10}H_{13}FNO_2S$ $[M+H]^+$: 230.0651; Found: 230.0646.

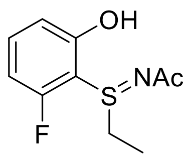

(*E*)-*N*-(ethyl(2-fluoro-6-hydroxyphenyl)-λ^4-sulfanylidene)acetamide, 3ka', white solid, 3ka:3ka'=1.05:1, yield: 84%

^{1}H NMR (400 MHz, CD_2Cl_2) δ 7.43 (dd, *J* = 15.2, 8.3 Hz, 1H), 6.79 (d, *J* = 8.5 Hz, 1H), 6.70 (t, *J* = 9.0 Hz, 1H), 3.41 (dq, *J* = 14.7, 7.4 Hz, 1H), 3.19 (dq, *J* = 14.5, 7.3 Hz, 1H), 2.13 (s, 3H), 1.29 (t, *J* = 7.3 Hz, 3H); ^{13}C NMR (101 MHz, CD_2Cl_2) δ 179.70, 163.60, 161.35, 158.88, 134.84(*J* = 11.1 Hz), 115.78, 105.67(*J* = 21.2 Hz), 43.61, 23.81, 6.63; HRMS (ESI) calculated for $C_{10}H_{13}FNO_2S$ $[M+H]^+$: 230.0651; Found: 230.0646.

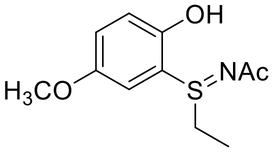

(*E*)-*N*-(ethyl(2-hydroxy-5-methoxyphenyl)-λ4-sulfanylidene)acetamide, colorless oil, 3la, yield: 88%

^{1}H NMR (500 MHz, $CDCl_3$) δ 6.90 (dd, *J* = 9.0, 3.0 Hz, 1H), 6.81 (d, *J* = 9.0 Hz, 1H), 6.78 (d, *J* = 3.0 Hz, 1H), 3.73 (s, 3H), 3.28 – 3.12 (m, 2H), 2.13 (s, 3H), 1.24 (t, *J* = 7.4 Hz, 3H); ^{13}C NMR (126 MHz, $CDCl_3$) δ 181.26, 153.73, 152.70, 120.53, 120.26, 112.75, 112.35, 55.97, 42.81, 24.26, 7.85; HRMS (ESI) calculated for $C_{11}H_{16}NO_3S$ $[M+H]^+$: 242.0851; Found: 242.0850.

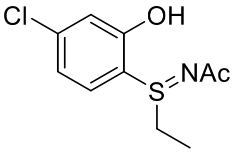

(*E*)-*N*-((4-chloro-2-hydroxyphenyl)(ethyl)-λ^4-sulfanylidene)acetamide, 3ma, white solid, 3ma:3ma'=1:1, yield: 85%

^{1}H NMR (500 MHz, MeOD) δ 7.50 (d, *J* = 8.5 Hz, 1H), 7.07 (dd, *J* = 8.5, 2.0 Hz, 1H), 7.00 (d, *J* = 2.0 Hz, 1H), 3.32 – 3.26 (m, 1H), 3.21 – 3.16 (m, 1H), 2.12 (s, 3H), 1.25 (t, *J* = 7.3 Hz, 3H); ^{13}C NMR (126 MHz, MeOD) δ 182.49, 156.89, 138.84, 128.28, 120.28, 116.16, 115.83, 40.41, 22.79, 6.48; HRMS (ESI) calculated for $C_{10}H_{13}ClNO_2S$ $[M+H]^+$: 246.0356; Found: 246.0350.

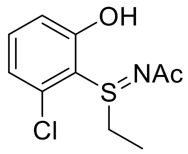

(*E*)-*N*-((2-chloro-6-hydroxyphenyl)(ethyl)-λ^4-sulfanylidene)acetamide, 3ma', white solid, 3ma:3ma'=1:1, yield: 85%

^{1}H NMR (400 MHz, CD_2Cl_2) δ 7.39 (t, *J* = 8.2 Hz, 1H), 6.99 (d, *J* = 8.0 Hz, 1H), 6.90 (d, *J* = 8.5 Hz, 1H), 3.44 (dt, *J* = 14.7, 7.3 Hz, 1H), 3.33 – 3.17 (m, 1H), 2.13 (s, 3H), 1.32 (t, *J* = 7.4 Hz, 3H); ^{13}C NMR (101 MHz, CD_2Cl_2) δ 179.47, 134.31, 133.48, 120.46, 119.09, 43.02, 23.94, 6.89; HRMS (ESI) calculated for $C_{10}H_{13}ClNO_2S$ $[M+H]^+$: 246.0356; Found: 246.0350.

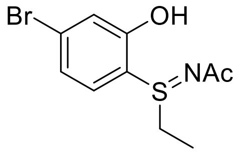

(*E*)-*N*-((4-bromo-2-hydroxyphenyl)(ethyl)-λ^4-sulfanylidene)acetamide, 3na’, white solid, 3na:3na’=1:1, yield: 86%

^{1}H NMR (500 MHz, MeOD) δ 7.43 (d, *J* = 8.5 Hz, 1H), 7.22 (dd, *J* = 8.5, 1.8 Hz, 1H), 7.18 – 7.12 (m, 1H), 3.32 – 3.25 (m, 1H), 3.18 (dd, *J* = 13.1, 7.3 Hz, 1H), 2.12 (s, 3H), 1.25 (t, *J* = 7.3 Hz, 3H); ^{13}C NMR (126 MHz, MeOD) δ 182.50, 156.83, 128.39, 126.81, 123.25, 119.15, 116.43, 40.32, 22.79, 6.48; HRMS (ESI) calculated for $C_{10}H_{13}BrNO_2S$ $[M+H]^+$: 289.9850; Found: 289.9845.

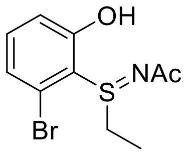

(*E*)-*N*-((2-bromo-6-hydroxyphenyl)(ethyl)-λ^4-sulfanylidene)acetamide, 3na’, white solid, 3na:3na’=1:1, yield: 86%

^{1}H NMR (400 MHz, CD_2Cl_2) δ 7.31 (t, *J* = 8.1 Hz, 1H), 7.16 (d, *J* = 7.8 Hz, 1H), 6.95 (d, *J* = 8.4 Hz, 1H), 3.44 (dq, *J* = 14.7, 7.4 Hz, 1H), 3.27 (dq, *J* = 14.6, 7.4 Hz, 1H), 2.13 (s, 3H), 1.33 (t, *J* = 7.3 Hz, 3H); ^{13}C NMR (101 MHz, CD_2Cl_2) δ 179.41, 163.82, 134.52, 123.84, 122.79, 119.77, 43.09, 23.96, 7.04; HRMS (ESI) calculated for $C_{10}H_{13}BrNO_2S$ $[M+H]^+$: 289.9850; Found: 289.9845.

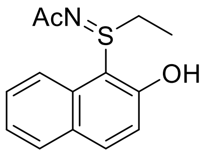

(*E*)-*N*-(ethyl(2-hydroxynaphthalen-1-yl)-λ^4-sulfanylidene)acetamide, 3oa, white solid, yield: 89%

^{1}H NMR (500 MHz, $CDCl_3$) δ 7.97 (d, *J* = 8.5 Hz, 1H), 7.88 (d, *J* = 9.0 Hz, 1H), 7.77 (d, *J* = 8.1 Hz, 1H), 7.57 – 7.50 (m, 1H), 7.38 (dd, *J* = 11.1, 4.0 Hz, 1H), 7.13 (d, *J* = 9.0 Hz, 1H), 3.47 – 3.32 (m, 1H), 3.21 (dd, *J* = 13.0, 7.4 Hz, 1H), 2.17 (s, 3H), 1.22 (t, *J* = 7.4 Hz, 3H); ^{13}C NMR (126 MHz, $CDCl_3$) δ 179.94, 162.82, 134.81, 131.86, 129.00, 128.42, 128.02, 124.03, 121.54, 120.48, 100.90, 42.90, 24.34, 7.51; **HRMS (ESI)** calculated for $C_{14}H_{16}NO_2S$ $[M+H]^+$: 262.0902; Found: 262.0895.

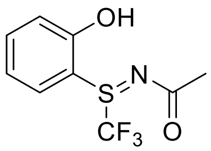

(*Z*)-*N*-((2-hydroxyphenyl)(trifluoromethyl)-λ^4-sulfanylidene)acetamide, 3ab, white solid, yield: 85%

^{1}H NMR (400 MHz, $CDCl_3$)： δ 11.77 (s, 1H), 7.52 (t, *J* = 7.8 Hz, 1H), 7.42 (d, *J* = 8.0 Hz, 1H), 7.01 (dd, *J* = 7.8, 3.8 Hz, 2H), 2.25 (s, 3H); ^{13}C NMR (101 MHz, $CDCl_3$): δ 181.70, 162.29, 136.40, 130.38, 124.33 (*J* = 334.3 Hz), 120.32, 120.20, 105.03, 24.41; ^{19}F NMR (376 MHz, $CDCl_3$)： δ -64.77（s, 3F）; HRMS (ESI) calculated for $C_9H_7F_3NO_2S$ [M–H]$^-$: 250.0156; Found: 250.0155; IR (cm^{-1}): 1541, 1452, 1363, 1315, 1296, 1190, 1097, 1003, 954, 800, 767, 754.

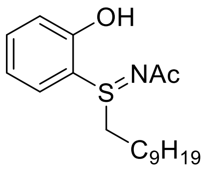

(*E*)-*N*-(decyl(2-hydroxyphenyl)-λ^4-sulfanylidene)acetamide, 3ac, white solid, yield: 85%

^{1}H NMR (500 MHz, $CDCl_3$): δ 7.28 – 7.24 (m, 2H), 6.86 – 6.81 (m, 2H), 3.11 (dd, *J* = 13.5, 7.3 Hz, 2H), 2.13 (s, 3H), 1.60 – 1.57 (m, 2H), 1.32 – 1.26 (m, 2H), 1.24 – 1.18 (m, 12H), 0.83 (t, *J* = 7.0 Hz, 3H); ^{13}C NMR (126 MHz, $CDCl_3$): δ 181.24, 159.50, 133.79, 127.94, 119.74, 119.17, 114.06, 48.63, 31.83, 29.39, 29.25, 29.20, 28.96, 28.29, 24.40, 23.33, 22.63, 14.08; HRMS (ESI) calculated for $C_{18}H_{28}NO_2S$ [M–H]$^-$: 322.1846; Found: 322.1847.

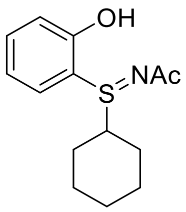

(*E*)-*N*-(cyclohexyl(2-hydroxyphenyl)-λ^4-sulfanylidene)acetamide, 3ad, white solid, yield: 76%

^{1}H NMR (500 MHz, $CDCl_3$): δ 7.37 – 7.34 (m, 1H), 7.18 (dd, *J* = 7.8, 1.5 Hz, 1H), 6.90 (d, *J* = 8.1 Hz, 1H), 6.88 – 6.85 (m, 1H), 3.32 (tt, *J* = 11.4, 3.5 Hz, 1H), 2.12 (s, 3H), 2.09 – 2.01 (m, 1H), 1.91 – 1.82 (m, 2H), 1.79 (dd, *J* = 7.9, 3.8 Hz, 1H), 1.66 – 1.58 (m, 1H), 1.39 (ddd, *J* = 23.9, 12.1, 3.5 Hz, 1H), 1.34 – 1.22 (m, 2H), 1.17 (ddd, *J* = 15.1, 8.8, 2.9 Hz, 2H). ^{13}C NMR (126 MHz, $CDCl_3$): δ 180.64, 161.39, 134.04, 129.87, 119.88, 119.30, 110.42, 60.05, 27.11, 26.53, 25.44, 25.33, 25.20, 24.63; HRMS (ESI) calculated for $C_{14}H_{18}NO_2S$ [M–H]$^-$: 264.1064; Found: 264.1063

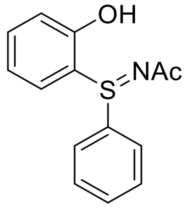

(*E*)-*N*-((2-hydroxyphenyl)(phenyl)-λ⁴-sulfanylidene)acetamide, 3ae, white solid, yield: 67%

^{1}H NMR (500 MHz, $CDCl_3$): δ 7.72 – 7.70 (m, 2H), 7.48 – 7.38 (m, 3H), 7.33 (t, *J* = 7.7 Hz, 2H), 6.92 (d, *J* = 8.1 Hz, 1H), 6.88 (t, *J* = 7.6 Hz, 1H), 2.18 (s, 3H). ^{13}C NMR (126 MHz, $CDCl_3$): δ 180.87, 159.87, 135.91, 134.11, 132.09, 129.86, 128.84, 127.49, 120.12, 119.90, 115.45, 24.34; HRMS (ESI) calculated for $C_{14}H_{12}NO_2S$ [M–H]$^-$: 258.0594; Found: 258.0596

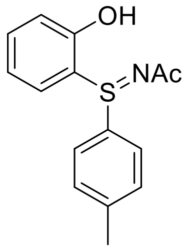

(*E*)-*N*-((2-hydroxyphenyl)(*p*-tolyl)-λ⁴-sulfanylidene)acetamide, 3af, white solid, yield: 85%

^{1}H NMR (400 MHz, $CDCl_3$) : δ 7.62 (d, *J* = 8.3Hz, 2H), 7.40 – 7.33 (m, 1H), 7.29 – 7.23 (m, 3H), 6.97 (dd, *J* = 8.4, 1.1 Hz, 1H), 6.87 (td, *J* = 8.0, 1.2 Hz, 1H), 2.35 (s, 3H), 2.17 (s, 3H). ^{13}C NMR (101 MHz, $CDCl_3$): δ 180.53, 160.15, 143.03, 134.01, 132.69, 130.57, 129.06, 127.49, 120.20, 120.03, 115.08, 24.27, 21.41; HRMS (ESI) calculated for $C_{15}H_{14}NO_2S$ [M–H]$^-$: 272.0751; Found: 272.0753; IR (cm^{-1}): 2916, 2846, 1585, 1541, 1521, 1508, 1490, 1448, 1363, 1298, 1280, 1219, 1178, 1157, 1116, 1006, 970, 848, 833.

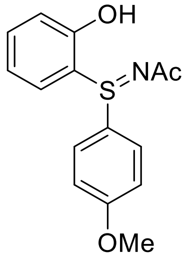

(*E*)-*N*-((2-hydroxyphenyl)(4-methoxyphenyl)-λ⁴-sulfanylidene)acetamide, 3ag, white solid, yield: 80%

^{1}H NMR (500 MHz, $CDCl_3$): δ 7.68 (d, *J* = 8.9 Hz, 1H), 7.36 – 7.29 (m, 1H), 7.28 – 7.22 (m, 1H), 6.93 (m, 3H), 6.86 (t, *J* = 7.5 Hz, 1H), 3.77 (s, 3H), 2.14 (s, 3H). ^{13}C NMR (126 MHz, $CDCl_3$): δ 180.51, 162.76, 159.91, 133.87, 129.84, 128.84, 126.84, 120.06, 115.52, 115.39, 55.61, 24.30; HRMS (ESI) calculated for $C_{15}H_{14}NO_3S$ [M–

H]$^-$: 288.0700; Found: 288.0698

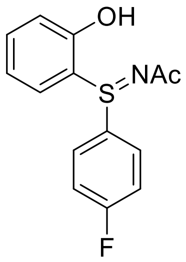

(*E*)-*N*-((4-fluorophenyl)(2-hydroxyphenyl)-λ^4-sulfanylidene)acetamide, 3ah, white solid, yield: 75%

^{1}H NMR (400 MHz, $CDCl_3$): δ 7.81 – 7.72 (m, 2H), 7.39 (ddd, *J* = 8.6, 7.3, 1.6 Hz, 1H), 7.30 – 7.25 (m, 1H), 7.21 – 7.10 (m, 2H), 6.99 (dd, *J* = 8.4, 1.0 Hz, 1H), 6.95 – 6.87 (m, 1H), 2.18 (s, 3H). ^{13}C NMR (101 MHz, $CDCl_3$): δ 180.66, 166.04, 163.51, 160.14, 134.31, 130.05 (*J*=4.5 Hz), 128.95, 120.27 (*J*=7.1 Hz), 117.38, 117.16, 114.60, 24.22; HRMS (ESI) calculated for $C_{14}H_{11}FNO_2S$ [M–H]$^-$: 276.0500; Found: 276.0502; IR (cm^{-1}): 2922, 2850, 1654, 1587, 1558, 1541, 1521, 1490, 1448, 1365, 1321, 1300, 1230, 1155, 1010, 831, 754.

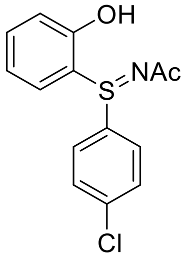

(*E*)-*N*-((4-chlorophenyl)(2-hydroxyphenyl)-λ^4-sulfanylidene)acetamide, 3ai, white solid, yield: 70%

^{1}H NMR (400 MHz, $CDCl_3$): δ 7.68 (d, *J* = 8.7 Hz, 2H), 7.48 – 7.38 (m, 3H), 7.28 (dd, *J* = 8.5, 2.0 Hz, 1H), 7.00 (d, *J* = 8.3 Hz, 1H), 6.92 (t, *J* = 7.6 Hz, 1H), 2.19 (s, 3H); ^{13}C NMR (101 MHz, $CDCl_3$): δ 180.69, 160.32, 138.72, 134.57, 134.47, 130.17, 129.14, 128.77, 120.49, 120.25, 114.20, 24.19; HRMS (ESI) calculated for $C_{14}H_{11}ClNO_2S$ [M–H]$^-$:292.0205; Found: 292.0204; IR (cm^{-1}): 2920, 2850, 1734, 1653, 1635, 1577, 1558, 1471, 1450, 1363, 1298, 1273, 1155, 1089, 1008, 833,812, 792, 752.

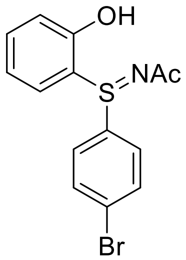

(*E*)-*N*-((4-bromophenyl)(2-hydroxyphenyl)-λ^4-sulfanylidene)acetamide, 3aj,

white solid, yield: 68%

^{1}H NMR (500 MHz, DMSO): δ 7.72 (m, 3H), 7.64 – 7.60 (m, 2H), 7.42 – 7.36 (m, 1H), 7.06 – 7.00 (m, 1H), 6.93 (d, *J* = 7.6 Hz, 1H), 1.98 (s, 3H); ^{13}C NMR (126 MHz, DMSO): δ 180.74, 156.63, 136.84, 134.33, 133.51, 130.64, 127.76, 126.18, 121.17, 120.69, 117.49, 25.23; HRMS (ESI) calculated for $C_{14}H_{11}BrNO_2S$ $[M–H]^-$: 335.9699; Found: 335.9697.

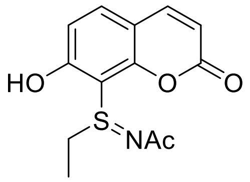

(*E*)-*N*-(ethyl(7-hydroxy-2-oxo-2*H*-chromen-8-yl)-λ^4-sulfanylidene)acetamide, 3pa, white solid, yield: 80%

^{1}H NMR (300 MHz, CD_2Cl_2):δ 7.70 – 7.67 (m, 1H), 7.58 (d, *J* = 19.0 Hz, 1H), 6.84 (d, *J* = 8.3 Hz, 1H), 6.23 (d, *J* = 9.2 Hz, 1H), 3.53 (dd, *J* = 12.7, 6.5 Hz, 1H), 3.27 (dd, *J* = 12.7, 6.5 Hz, 1H), 2.16 (s, 3H), 1.34 – 1.29 (m, 3H); ^{13}C NMR (126 MHz, CD_2Cl_2): δ 179.49, 158.97, 153.92, 143.89, 133.36, 117.44, 112.75, 111.25, 103.46, 43.59, 24.23, 7.00; HRMS (ESI) calculated for $C_{13}H_{14}NO_4S$ $[M+H]^+$: 280.0644; Found: 280.0640.

Supplementary discussion

Synthesis of deuterated substrate 1a-*d₈*

Following a literature report[1], in a 50mL round-bottom flask, *N*-hydroxyphthalimide (1.0 eq.), cooper (I) chloride (1.0 eq.), freshly activated 4 Å molecular sieves (250mg/mmol), and D5-phenylboronic acid (2.0 eq.) were combined in 1,2-dichloroethane(0.2 M). The pyridine (1.1 eq.) was then added to the suspension. The reaction mixture was open to the atmosphere and stirred at room temperature over 24 h. Upon completion, filtered through a celite pad, the filtrate was concentrated to give the crude deuterated *N*-aryloxyphthalimides.

Hydrazine monohydrate (3.0 eq.) was added to the solution of the crude deuterated *N*-aryloxyphthalimide (1.0eq.) in 10% MeOH in $CHCl_3$ (0.1 M). The reaction was allowed to stir at room temperature over 12 h. Upon completion, the reaction mixture was filtered off and washed with CH_2Cl_2. The filtrate was concentrated under reduced pressure, and purified by flash silica gel column chromatography to give the corresponding deuterated *N*-aryloxyamine.

To a solution of deuterated *N*-aryloxyamine and D4-acetic acid (1 eq.) in CH_2Cl_2 (0.4 M) at 0℃ were added HOBt (1.1 eq.) and EDCI (1.1 eq.). The reaction mixture was stirred at room temperature for 10 h, then washed with 5% aqueous HCl (3×15.0 ml), 5% aqueous $NaHCO_3$ (20 .0 ml), H_2O (20.0 ml), and brine (20.0 ml), and dried (Na_2SO_4). Purification by flash chromatography afforded the corresponding deuterated *N*-phenoxyamide (**1a**-*d₈*) as a white solid. ^{1}H NMR (400 MHz, DMSO) δ 11.67 (s, 1H); ^{13}C NMR (101 MHz, DMSO) δ 167.67, 159.92, 129.38 (t, J = 24.3 Hz), 122.17 (t, J = 24.2 Hz), 112.93 (t, J = 24.1 Hz), 19.17 (t, J = 19.4 Hz); HRMS (ESI) calculated for $C_8H_2D_8NO_2$ $[M+H]^+$: 160.1214; Found: 160.1208.

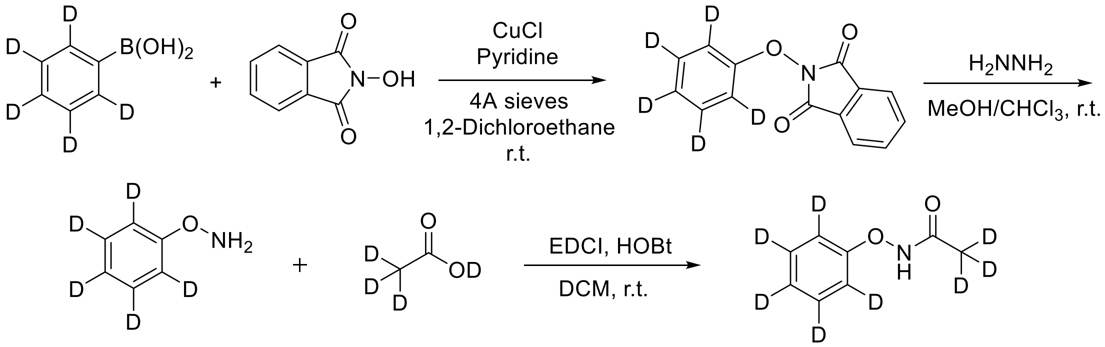

1a-*d₈*

1. Cross-over Experiment

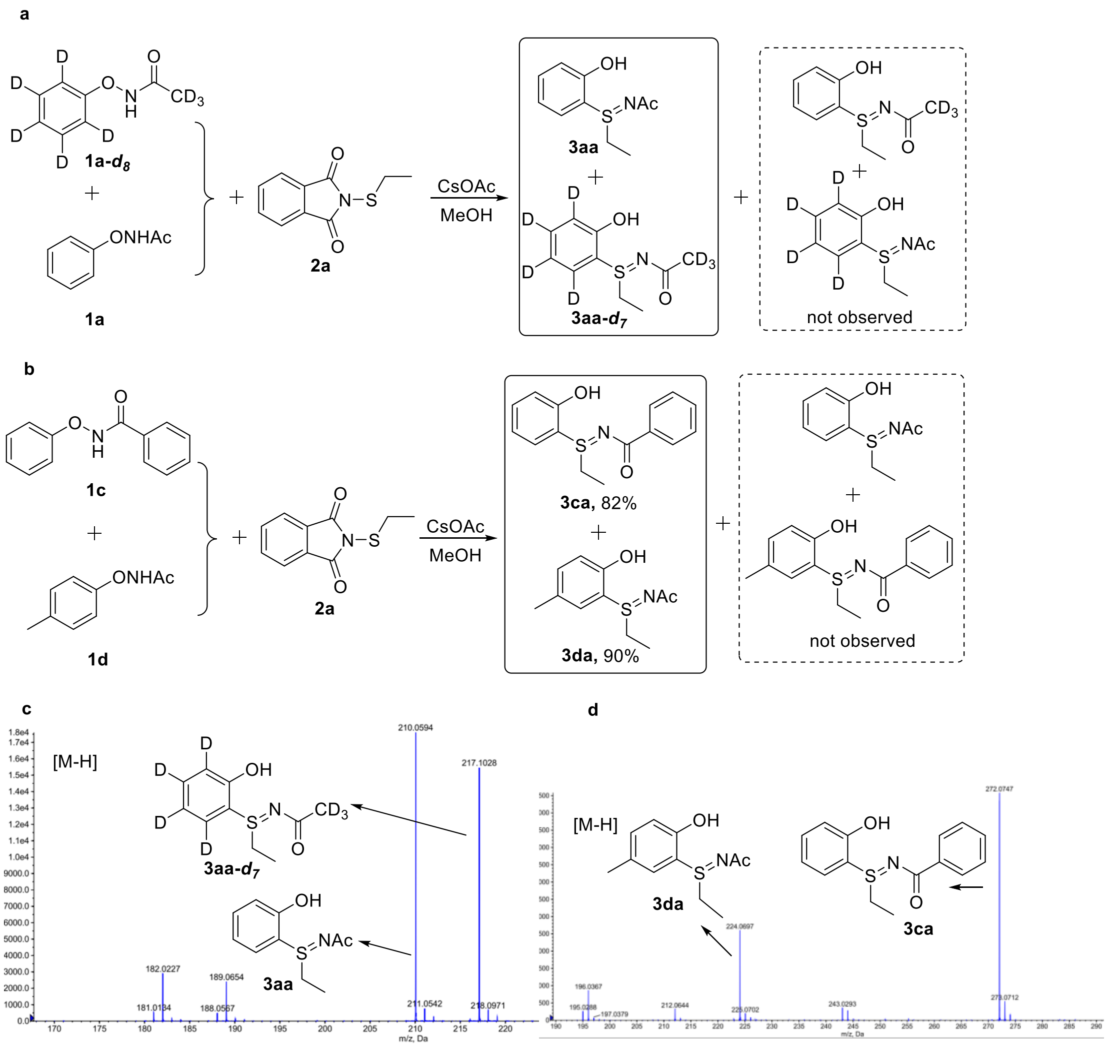

Supplementary Figure 36. Cross-over experiments. (**a**) Reaction conditions: **1a** (0.02 mmol), **1a-*d*$_8$** (1 eq.), **2a** (2.4 eq.) and CsOAc (1 eq.) in MeOH at room temperature for 3 h. (**b**) Reaction conditions: **1c** (0.1 mmol), **1d** (1 eq.), **2a** (2.4 eq.) and CsOAc (1 eq.) in MeOH at room temperature for 3 h. (**c**) HR-MS spectrum of (a). (**d**) HR-MS spectrum of (b).

we carried out the cross-over experiments using a 1:1 mixture of *N*-phenoxyacetamide **1a** and its analogue **1a**-d_8 under the standard condition, only the intramolecular rearrangement products **3aa** and **3aa**-d_7 were obtained (Supplementary Fig. 36a), suggesting an intramolecular process. The cross-over experiment between **1c**, **1d** and **2a** confirmed this conclusion (Supplementary Fig. 36b).

DFT Calculations

COMPUTATIONAL DETAILS

All the calculations were performed with the Gaussian09 suite of programs.[5] Geometry optimization and energy calculations were conducted with B3LYP.[6] LANL2DZ + d (0.289) basis set with ECP was used for I and 6-31G (d) basis set was used for atoms.[7-9] To verify the stationary points as minima or transition states, vibration frequency calculation at the same level of theory was performed for each structure. The zero-point energy, thermal energy, entropy, and free energy were also derived from vibration frequency. Single point energies were calculated at the B3LYP-D[10]/SDD[11]-6-311++G (d, p) level. A solvent correction for methanol at 298 K was calculated by using SMD solvation model.[12]

As shown in Fig. 2a, for the internal oxidants containing the X–N bond (X=N, S, O), only when X is O, the desired phenol product can be obtained. When N–H was replaced by N–Me, no reaction occurred. Furthermore, base (such as CsOAc) was an indispensable component for this reaction (Supplementary Table 6). These results indicated that the acidity of the N–H is important for this reaction and the N–H bond might be deprotoned by the base to initiate the following reaction.[13]

The *N*-sulfenylation step (**TS1**) was found to be the rate-determining step (RDS). By examining the structure of **TS1**, we envisioned that the energy barrier of nucleophilic substitution process could be influenced by the steric hindrance of the *N*-substituted thiophthalimides. DFT calculation revealed the activation free energy of the reaction between **INT1** and *N*-t-butylthiophthalimide was indeed 8 kcal/mol higher (**TS1'**) than that of *N*-ethylthiophthalimide, which implied that *N*-sulfenylation was unlikely to occur for the bulky *N*-t-butylthiophthalimide. The experiment was then conducted and confirmed the computational results (supplementary Fig. 37b).

The calculation results speculated that the reaction process containing two main stages: (1) *N*-sulfenylation. (2) [2, 3] sigmatropic rearrangement and aromatization.

First, the amide (NH) of the substrate could be deprotonated under weak basic conditions such as $^{-}$OAc[13] to form the intermediate **INT1**. Next, nucleophilic substitution by the intermediate **INT1** to *N*-ethylthiophthalimide led to the formation of the N–S bond (intermediate **INT2**) via **TS1**, and the computational results indicated that the barrier (25.7 kcal/mol) was accessible under reaction condition. Subsequently, [2,3]-sigmatropic rearrangement occurred through **TS2** to form the intermediate **INT3**. Finally, the intermediate **INT3** would easily tautomerize to the product. (supplementary Fig. 37a)

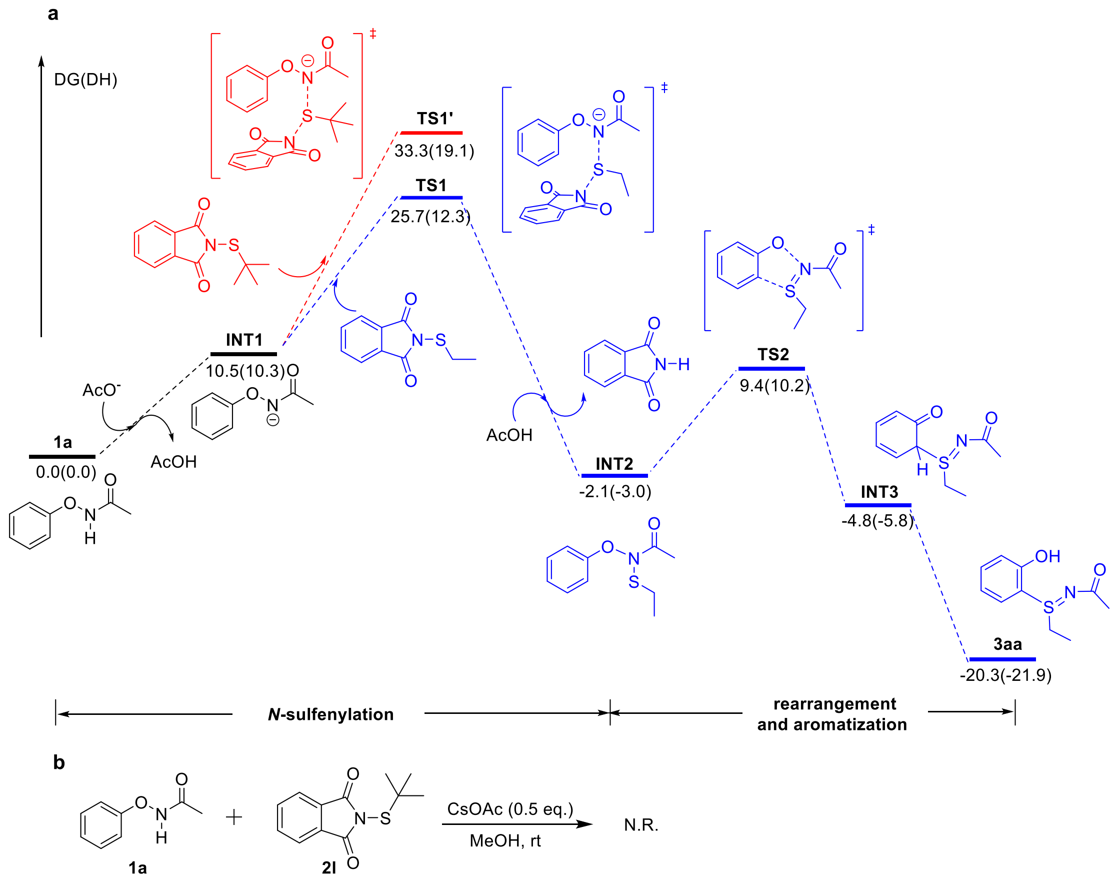

Supplementary Figure 37. **Mechanistic studies**. (**a**) Free energy (enthalpy) profile of the cascade reaction (in kcal/mol). (**b**) The reaction of *N*-phenoxyacetamide **1a** and *N*-tert-butylthiophthalimide **2l** under the standard conditions.

Cartesian coordinates (in Å) of related structures which calculated at the B3LYP/ Lanl2dz + 6-31G(d) level of theory.

substrate

H	1.77840500	0.41768400	-1.73984800
C	-3.31471200	0.44965200	0.34241100
C	-3.00317100	-0.88224900	0.05235000
C	-1.70086900	-1.24661900	-0.28149500
C	-0.70556200	-0.26609600	-0.32259700
C	-0.99520300	1.06701200	-0.03754900
C	-2.30833000	1.41405800	0.29458800
H	-4.33131800	0.73044200	0.60135300
H	-3.77734900	-1.64404600	0.08429000
H	-1.44012300	-2.27496200	-0.51192500
H	-0.21047900	1.81260400	-0.07697300
H	-2.53832600	2.45257000	0.51733500
O	0.55773000	-0.74245500	-0.66094000
N	1.54434800	0.25843400	-0.76241500

C	2.66222600	0.11091400	0.06612700
C	2.43361300	-0.63200600	1.36268300
H	1.55113100	-0.25254600	1.88759000
H	2.26202800	-1.69563200	1.16476800
H	3.32093100	-0.51398000	1.98593700
O	3.71310800	0.62630900	-0.26840200

AcO-

C	0.22053200	0.00178300	-0.00106300
C	-1.35408100	-0.05450700	-0.00033900
H	-1.73363300	-1.08424200	-0.02416900
H	-1.74827500	0.44994200	0.89452100
H	-1.75273600	0.49513200	-0.86587300
O	0.80791000	-1.10901600	0.00025600
O	0.69658200	1.16595400	0.00023600

AcOH

C	1.39762100	-0.10999800	-0.00003500
H	1.68532900	-0.69251800	-0.88155300
H	1.68548200	-0.69079300	0.88258300
H	1.91733100	0.84832500	-0.00096600
C	-0.09247300	0.12550000	-0.00006500
O	-0.77890300	-1.04651900	0.00020700
H	-1.72387800	-0.80241700	0.00027600
O	-0.64549200	1.20206800	-0.00017500

1

C	-3.50291100	-0.25949800	-0.00041500
C	-2.99496400	1.04628100	-0.00045100
C	-1.62348300	1.27754800	-0.00025200
C	-0.71030700	0.19834600	0.00002400
C	-1.22005100	-1.11823300	0.00006400
C	-2.59854100	-1.32665000	-0.00015100
H	-4.57604700	-0.43802800	-0.00059600
H	-3.67720100	1.89583800	-0.00064900
H	-1.22275900	2.28813700	-0.00028700
H	-0.50713900	-1.93152100	0.00026100
H	-2.97424800	-2.34967300	-0.00012000
O	0.58631400	0.50717000	0.00027100
N	1.47777200	-0.69207000	0.00005600
C	2.74405700	-0.25420300	0.00020200
C	3.05444000	1.25660900	0.00061100
H	2.63070300	1.75813500	-0.87823400
H	2.63066500	1.75766600	0.87970200

H	4.14220200	1.37142300	0.00065500
O	3.70368300	-1.06075600	-0.00013500

***N*-ethylthiophthalimide**

C	0.12103600	-1.17931700	-0.25553700
C	0.12122800	1.17948900	-0.25536100
N	-0.66433400	0.00021100	-0.40061700
O	-0.29073600	-2.31589200	-0.29914600
O	-0.29029600	2.31620300	-0.29877000
C	1.51436200	0.69790400	-0.03003200
C	2.68187000	1.42388000	0.16479800
C	3.86541700	0.70079700	0.35952700
C	3.86531600	-0.70123200	0.35942900
H	4.79971000	-1.23348700	0.51345300
C	2.68167100	-1.42409100	0.16465100
H	2.66850700	-2.50952900	0.16335000
C	1.51426800	-0.69793800	-0.03013100
H	4.79988800	1.23288100	0.51373100
H	2.66885500	2.50931600	0.16362600
S	-2.34199900	0.00030600	-0.76485900
C	-3.06899600	-0.00041000	0.92628900
H	-2.71820200	-0.89295900	1.45238300
H	-2.71794500	0.89156400	1.45317900
C	-4.59322800	-0.00014700	0.80101600
H	-4.95152000	-0.88803800	0.26898900
H	-5.04509100	-0.00065900	1.79882600
H	-4.95129400	0.88843300	0.26997500

***N*-*t*-butylthiophthalimide**

C	0.54333100	-1.17868300	-0.38588300
C	0.54333800	1.17867000	-0.38587600
N	-0.24353300	0.00002100	-0.54428500
O	0.14628800	-2.31739200	-0.48219500
O	0.14635000	2.31741200	-0.48207400
C	1.92014300	0.69760800	-0.07684200
C	3.07469400	1.42402100	0.18340300
C	4.24498300	0.70107700	0.44615100
C	4.24496400	-0.70107800	0.44623900
H	5.16919900	-1.23316200	0.65314000
C	3.07468200	-1.42404700	0.18351400
H	3.06183900	-2.50947500	0.17991300
C	1.92013300	-0.69766000	-0.07684800
H	5.16919900	1.23315900	0.65313200
H	3.06180300	2.50944800	0.17972900

S	-1.86676600	0.00001900	-1.11217800
C	-2.90023100	0.00000700	0.45087200
C	-4.33571900	-0.00031500	-0.10430300
H	-4.53396000	-0.89043300	-0.71171300
H	-5.04449600	-0.00027400	0.73225400
H	-4.53427500	0.88949500	-0.71205900
C	-2.63752100	-1.26555400	1.27554200
H	-2.80853500	-2.17071300	0.68622300
H	-1.60830700	-1.29505900	1.64629400
H	-3.30582800	-1.28162700	2.14695100
C	-2.63790500	1.26586100	1.27519300
H	-1.60869600	1.29577800	1.64591200
H	-2.80921700	2.17080600	0.68562800
H	-3.30619300	1.28201400	2.14662700

TS1

C	4.97026100	-3.01097700	-0.21965700
C	4.46719900	-2.76606500	-1.50195700
C	3.68431000	-1.64311000	-1.75347500
C	3.39791700	-0.74137700	-0.71618800
C	3.89612700	-0.97666700	0.57076300
C	4.67500800	-2.11146700	0.80627500
H	5.57630600	-3.89206700	-0.02436700
H	4.68106100	-3.45870600	-2.31333400
H	3.27868000	-1.44332400	-2.74082600
H	3.66018400	-0.27535000	1.36076300
H	5.05394900	-2.29030200	1.81034600
O	2.65951000	0.34887800	-1.07541800
N	2.19750200	1.15094000	0.02563200
C	2.71917200	2.42061200	-0.04105500
C	3.84473700	2.68792400	-1.04043100
H	4.68201800	1.99496800	-0.90393600
H	3.48911600	2.56565700	-2.06913800
H	4.18628600	3.71410300	-0.88683700
O	2.32344600	3.31092500	0.71437700
S	0.19176100	0.79811700	0.14240300
C	0.19589400	1.22354000	1.94242200
C	0.60181000	0.06946100	2.85553100
H	0.58314100	0.39428800	3.90718700
H	-0.08232300	-0.77387400	2.72986700
H	1.61728300	-0.26987100	2.62533800
C	-2.80222400	1.02899400	-0.59558500
C	-2.47346500	-0.79042400	0.73416900
N	-1.90310000	0.36340000	0.22717500

O	-2.62645300	2.06825100	-1.21596600
O	-1.96994500	-1.61341300	1.49206100
C	-3.88774000	-0.87719700	0.20707300
C	-4.88848000	-1.81529900	0.41587000
C	-6.11935700	-1.60439100	-0.22299400
C	-6.32361500	-0.48307500	-1.03874700
H	-7.28855800	-0.34394900	-1.52187300
C	-5.30244300	0.45776100	-1.23858300
H	-5.44507700	1.33238800	-1.86770500
C	-4.09023100	0.23508900	-0.60164700
H	-6.92874700	-2.31843700	-0.08558600
H	-4.71462700	-2.67910800	1.05201200
H	-0.82242200	1.55416900	2.15533700
H	0.86299700	2.08206100	2.03352400

TS1’

C	-4.99991900	3.04311100	0.67916600
C	-4.22693200	3.42543500	-0.42252300
C	-3.36374700	2.51918700	-1.03140500
C	-3.26805300	1.20628700	-0.54404600
C	-4.03623100	0.81294700	0.55829800
C	-4.89274200	1.73713800	1.16035000
H	-5.66822800	3.75477700	1.15734100
H	-4.29027500	4.44114400	-0.80720100
H	-2.74845700	2.80350300	-1.87969500
H	-3.94484800	-0.20032800	0.92723400
H	-5.48331400	1.42524300	2.01935600
O	-2.43090700	0.38249600	-1.23936700
N	-2.15365000	-0.87475200	-0.59425300
C	-2.56967000	-1.92242100	-1.37769300
C	-3.43514100	-1.61725100	-2.59940100
H	-4.32536800	-1.03766700	-2.33244200
H	-2.87530600	-1.02785100	-3.33356300
H	-3.73392100	-2.57069300	-3.04084800
O	-2.29089700	-3.08655200	-1.07880900
S	-0.18868400	-0.68818500	-0.01032200
C	-0.26279800	-1.81672300	1.53506100
C	0.55847400	-1.18497300	2.67290000
H	0.36837900	-1.75025100	3.59749700
H	1.62885100	-1.22122300	2.46641700
H	0.28218100	-0.13916400	2.83552000
C	2.90182600	-0.87538600	-0.45008300
C	2.30565800	1.01220200	0.67678300
N	1.89985000	-0.25323600	0.27690500

O	2.89146100	-2.00169500	-0.93248000
O	1.67823700	1.82323900	1.34568900
C	3.70926900	1.23032800	0.15791200
C	4.57691900	2.30594100	0.28179800
C	5.83965800	2.19430100	-0.31886900
C	6.20585600	1.03332100	-1.01349400
H	7.19195100	0.97347600	-1.46941700
C	5.31957300	-0.04817400	-1.12620600
H	5.58946500	-0.95600100	-1.65916700
C	4.07268700	0.07846200	-0.53099100
H	6.54715500	3.01772800	-0.24624400
H	4.27878600	3.19845500	0.82551800
C	-1.71293100	-1.95830500	2.02455200
H	-1.70142400	-2.56887500	2.93967900
H	-2.15057200	-0.98584800	2.27170100
H	-2.33953300	-2.45991000	1.28660900
C	0.29525800	-3.19507600	1.15029900
H	-0.34464500	-3.65517000	0.39278900
H	1.30633400	-3.10945800	0.74266900
H	0.32309600	-3.84700700	2.03813200

Phthalimide

C	-1.24304600	-1.17403200	-0.00003000
C	-1.24290600	1.17413200	0.00004500
N	-2.01164300	0.00004900	0.00007400
O	-1.67366800	-2.30800000	-0.00019800
O	-1.67344400	2.30810100	0.00013700
C	0.17502900	0.69884400	-0.00004200
C	1.35811000	1.42401400	-0.00020000
C	2.55814000	0.70064300	-0.00018300
C	2.55809800	-0.70075300	0.00012500
H	3.50507100	-1.23306300	0.00031900
C	1.35803300	-1.42410200	0.00019600
H	1.34627200	-2.50963400	0.00038300
C	0.17497900	-0.69891800	-0.00001400
H	3.50513900	1.23289400	0.00006800
H	1.34633300	2.50955200	-0.00026200
H	-3.02303200	0.00013900	0.00006300

2

C	3.79545800	-0.71321400	0.60496400
C	3.75254200	-0.02956800	-0.61376700
C	2.55380600	0.50205700	-1.08473900
C	1.39203900	0.35446600	-0.32217300

C	1.41630500	-0.32129300	0.89814900
C	2.62572800	-0.85610100	1.35154700
H	4.73036100	-1.13157500	0.96584700
H	4.65516800	0.08461700	-1.20781100
H	2.49853300	1.02785800	-2.03261700
H	0.50812100	-0.43061000	1.47922300
H	2.64625100	-1.38450500	2.30095300
O	0.26557700	0.96881600	-0.85731700
N	-0.96777100	0.51960500	-0.31153800
C	-1.67323800	1.50735900	0.41384800
C	-0.87908000	2.74669200	0.77483500
H	0.04489400	2.49305500	1.30376900
H	-0.59567600	3.29885500	-0.12673400
H	-1.50997700	3.37051600	1.40951400
O	-2.82412700	1.31643600	0.74753200
S	-1.78925500	-0.64816000	-1.28227900
C	-1.62669900	-2.17659000	-0.27506100
H	-1.96769700	-2.95699400	-0.96809100
H	-0.56521100	-2.35564700	-0.08231600
C	-2.45416200	-2.18202000	1.00735800
H	-3.51811600	-2.04906400	0.79063000
H	-2.32005300	-3.13422700	1.53590900
H	-2.15191800	-1.37168900	1.67746500

TS2

S	1.07516700	-0.76598100	-0.91529100
N	1.33788400	0.38324800	0.26107200
O	1.05714700	2.03417500	-1.36432400
O	-0.14023900	0.61900700	1.33849400
C	1.32212600	1.73996200	-0.21544300
C	-2.02308100	1.09880500	-0.05359400
C	-1.25047300	0.21072900	0.73518700
C	-1.68601100	-1.12690000	0.88148900
C	-2.88567600	-1.54569100	0.30950900
C	1.75522200	2.73146100	0.83838400
C	-3.64918800	-0.65109200	-0.44413200
C	-3.21344600	0.66912800	-0.62557000
C	1.80393200	-2.20475400	-0.03526000
H	-1.08691800	-1.79818200	1.48928100
H	-3.22424000	-2.56928400	0.44611300
H	1.28106200	2.50916300	1.79544000
H	1.51127400	3.74304700	0.50759700
H	2.84145000	2.65284500	0.97087500
H	-4.58301100	-0.97877700	-0.89286100

H	-3.81012100	1.35936800	-1.21577100
H	-1.67369200	2.11785800	-0.18364400
H	1.52667400	-3.06798100	-0.65156600
H	1.29043000	-2.29840500	0.92562000
C	3.31583700	-2.10231600	0.14822300
H	3.56777600	-1.21046700	0.72879500
H	3.68916100	-2.98135000	0.68723000
H	3.83157500	-2.04632300	-0.81606800

3

S	-0.59054000	0.33333300	-0.57802600
N	-1.50482600	-1.01885200	-0.16099300
O	-3.23215800	0.49246000	-0.40929100
O	0.98127800	-0.85119000	1.89922300
C	-2.81198800	-0.66732800	-0.22456300
C	1.06786000	-0.49150000	-0.49975800
C	1.67453500	-0.67492200	0.91085100
C	3.14353100	-0.64605500	0.98423900
C	3.89834600	-0.21939400	-0.05457300
C	-3.77692800	-1.82741200	-0.03814000
C	3.31261000	0.17710700	-1.32696700
C	1.98754200	0.08204600	-1.53778500
C	-0.46020700	1.56717000	0.81707800
H	3.57351100	-0.89264700	1.95020100
H	4.97734400	-0.14293100	0.05855300
H	-4.41642900	-1.90296800	-0.92435300
H	-4.42855300	-1.61881400	0.81699700
H	-3.25654200	-2.77382500	0.12150200
H	3.96872700	0.54785700	-2.10907100
H	1.54989600	0.36702500	-2.49100300
H	0.77431600	-1.50823000	-0.80978100
H	0.59690000	1.85225600	0.85567000
H	-0.70212700	1.01231700	1.72523200
C	-1.35685600	2.77850300	0.58167800
H	-2.40130100	2.47645200	0.50160600
H	-1.23863300	3.47240400	1.42256000
H	-1.07832300	3.31030100	-0.33456800

pro

N	-1.64450800	-0.35616300	-0.46568000
C	1.29826900	-0.93177300	-0.69265200
C	0.93274400	-0.00269000	0.31276200
C	2.59861700	-1.46592600	-0.64162500
C	1.81751400	0.34105000	1.34390200

C	3.47988400	-1.09980100	0.36866700
H	2.88113900	-2.17744800	-1.41109000
C	3.09818000	-0.20084700	1.37381100
H	1.49356100	1.03454300	2.11593500
H	4.47681300	-1.53209400	0.38396300
H	3.78707500	0.06624500	2.16877200
C	-2.78552600	-0.82387900	0.18165300
C	-3.21131800	-0.24851200	1.53626400
H	-3.34995000	0.83876800	1.50005700
H	-4.15556700	-0.71807700	1.81488500
H	-2.46831500	-0.45752300	2.31558900
O	-3.44994800	-1.69526800	-0.35636700
O	0.48292600	-1.30911100	-1.68668700
S	-0.68643300	0.76177600	0.29637900
C	-0.47832500	2.01652000	-1.04547700
H	-0.47383200	-1.09915400	-1.42347300
H	-0.17529700	1.45373300	-1.93295800
H	-1.49568900	2.38795100	-1.20328200
C	0.49673800	3.12964700	-0.68102100
H	1.50305800	2.73952400	-0.50233900
H	0.55520400	3.84192500	-1.51106700
H	0.17180000	3.67863700	0.20944100

Supplementary References

1. Petrassi, H. M., Sharpless, K. B. & Kelly, J. W. The copper-mediated cross-coupling of phenylboronic acids and *N*-hydroxyphthalimide at room temperature: synthesis of aryloxyamines. *Org. Lett.* **3**, 139-142 (2001).
2. Xu, C. & Shen, Q. Palladium-catalyzed trifluoromethylthiolation of aryl C-H bonds. *Org. Lett.* **16**, 2046-2049 (2014).
3. Gillis, H. M., Greene, L. & Thompson, A. Preparation of sulfenyl pyrroles. *Synlett.* **1**, 112-116 (2009).
4. Castellino, A. J. & Rapoport, H. Synthesis of phenoxyamines. *J. Org. Chem.* **49**, 1348-1352 (1984).
5. M. J. Frisch, et al. *Gaussian 09, Rev. D.01*, Gaussian, Inc.: Wallingford, CT (2010).
6. Becke, A. D. Density-functional thermochemistry. III. The role of exact exchange. *J. Chem. Phys.* **98**, 5648-5652 (1993).
7. Ehlers, A. W., Böhme, M., Dapprich, S., Gobbi, A., Höllwarth, A., Jonas, V., Köhler, K. F., Stegmann, R., Veldkamp, A. & Frenking, G. A set of f-polarization functions for pseudo-potential basis sets of the transition metals Sc-Cu, Y-Ag and La-Au. *Chem. Phys. Lett.* **208**, 111-114 (1993).
8. Hay, P. J. & Wadt, W. R. Ab initio effective core potentials for molecular calculations. Potentials for K to Au including the outermost core orbitals. *J. Chem. Phys.* **82**, 299-310

(1985).
9. Roy, L. E., Hay, P. J. & Martin, R. L. Revised basis sets for the LANL effective core potentials. *J. Chem. Theory Comput.* **4**, 1029-1031 (2008).
10. Grimme, S., Antony, J., Ehrlich, S. & Krieg, H. A consistent and accurate ab initio parametrization of density functional dispersion correction (DFT-D) for the 94 elements H-Pu. *J. Chem. Phys.***132**, 154104 (2010).
11. Andrae, D., Häußermann, U., Dolg, M., Stoll, H. & Preuß, H. Energy-adjusted *ab initio* pseudopotentials for the second and third row transition elements. *Theoret. Chim. Acta.* **77**, 123-141 (1990).
12. Marenich, A. V., Cramer, C. J. & Truhlar, D. G. Universal solvation model based on solute electron density and on a continuum model of the solvent defined by the bulk dielectric constant and atomic surface tensions. *J. Phys. Chem. B* **113**, 6378-6396 (2009).
13. Liu, G., Shen, Y., Zhou, Z. & Lu, X. Rhodium(III)-catalyzed redox-neutral coupling of *N*-phenoxyacetamides and alkynes with tunable selectivity. *Angew. Chem. Int. Ed.* **52**, 6033-6037 (2013).
